# Cooling-induced intensification of ocean anoxia in the mid-Paleozoic

**DOI:** 10.1126/sciadv.aec8573

**Published:** 2026-03-13

**Authors:** Yuxuan Wang, Paul B. Wignall, Benjamin J. W. Mills, Alexander J. Dickson, David K. Loydell, Yijun Xiong, Zhen Xu, Jeffrey Peakall, Simon W. Poulton

**Affiliations:** ^1^ School of Earth and Environment, University of Leeds, Leeds LS2 9JT, UK.; ^2^ Organic and Earth Surface Geochemistry, GFZ Helmholtz Centre for Geosciences, 14473 Potsdam, Germany.; ^3^ Centre of Climate, Ocean and Atmosphere, Department of Earth Sciences, Royal Holloway University of London, Egham TW20 0EX, UK.; ^4^ School of the Environment and Life Sciences, University of Portsmouth, Burnaby Road, Portsmouth PO1 3QL, UK.

## Abstract

Mid-Paleozoic oceanic anoxic events (OAEs) have long posed an enigma, with their drivers and dynamics being markedly distinct from the hyperthermal-related events of later eras. Here, we investigate a prominent mid-Silurian OAE, which was associated with the Ireviken Extinction Event and coincided with a cooling climate. We apply Fe speciation, redox-sensitive trace metals, and elemental weathering proxies, alongside sedimentological records and coupled uranium-molybdenum isotope analyses, to deep shelf and basinal sections from the UK. These data demonstrate a gradual spread of anoxia from basinal to shelfal settings, which we postulate was driven by an enhanced nutrient supply delivered via cooling-induced upwelling. Isotope mass balance modeling supports a major increase in the extent of deeper water ferruginous conditions at this time, while euxinia developed on the continental shelf, stressing the shallower water biota. A subsequent transition to ferruginous anoxia occurred on the shelf during the later stages of the event, as climatic conditions recovered and terrestrial chemical weathering rates increased. These changes, occurring when the ocean was poised at a lower redox state under the prevailing, low atmospheric oxygen levels of the mid-Paleozoic, led to OAE dynamics that were markedly different to those of the Mesozoic.

## Teaser

Cooling-induced upwelling drove a mid-Silurian anoxic event, expanding ferruginous deep waters and euxinic shelves.

## INTRODUCTION

Silurian oceans underwent repeated perturbations that had a major impact on both the global carbon cycle and the biosphere (*1*–*3*). These episodes commonly led to enhanced ocean deoxygenation [termed oceanic anoxic events (OAEs)] characterized by positive carbon isotope excursions (CIEs), with prevalent examples being the early Sheinwoodian [ESCIE; ~432.5 million years ago (Ma)], mid-Homerian (~428.5 Ma), and mid-Ludfordian (~423.5 Ma) events (*4*–*8*). However, unlike Mesozoic OAEs, which are generally associated with hyperthermals, Paleozoic examples have been linked to intervals of cooling (*1*, *5*, *9*). Paleozoic OAEs are also distinct from their Mesozoic counterparts in that they occurred against a backdrop of generally low atmospheric and oceanic oxygenation levels, with near-modern levels of oxygenation only developing during the Late Silurian to Devonian (the Paleozoic Oxygenation Event) (*10*, *11*).

Many models for Paleozoic OAEs nevertheless invoke similar conditions to those proposed for Mesozoic OAEs (*12*, *13*), while others emphasize distinctive Paleozoic features, including the absence of a link to major flood basalt eruptions (*14*), and the general prevalence of a background oceanic state characterized by expanded ferruginous anoxia (*15*). Overall, the causes of Paleozoic OAEs remain poorly understood. Here, we focus on the OAE that spans the latest Telychian to Sheinwoodian stages of the Silurian, which occurred in association with global cooling (*5*, *16*). The interval includes the ESCIE (*17*, *18*) and is associated with the Ireviken Extinction Event (IEE), which saw severe losses among conodonts and graptolites (*19*, *20*), and a major turnover of acritarchs (*21*).

**Fig. 1.**
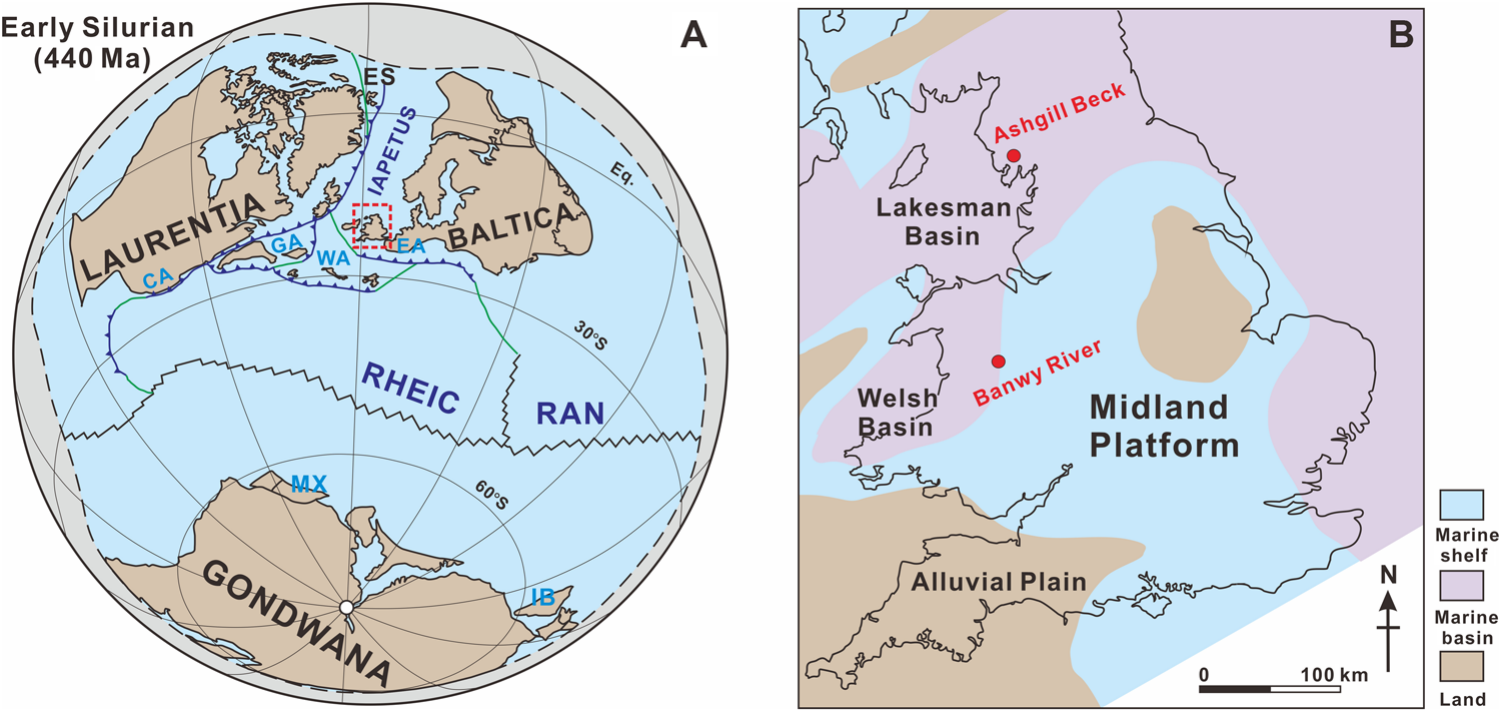
Paleogeographic context of the Silurian study area. (**A**) Global paleogeographic reconstruction during the Llandovery, prepared by the authors on the basis of published paleogeographic frameworks (*67*). CA, Carolinia; GA, Ganderia; WA, West Avalonia; EA, East Avalonia; MX, Mixteca-Oaxaca; IB, Iberia. Solid blue lines represent subduction zones, black lines represent spreading centers, and green lines represent transform plate margins. (**B**) Paleogeography of the UK during the mid-Silurian, reconstructed by the authors on the basis of published geological and paleogeographic frameworks (*67*).

We investigate Llandovery-Wenlock boundary sections from the River Banwy in Wales and Ashgill Beck in England (Fig. 1). These record outer-shelf to basin settings on the Eastern Avalonian margin between Laurentia and Baltica (see the Supplementary Materials) (*22*, *23*). We reconstruct oceanic redox dynamics and climate using Fe speciation, redox-sensitive trace metals, and elemental weathering proxies. Global redox variability is assessed using U and Mo isotope data within the framework of a coupled U-Mo isotope mass balance model. Our approach allows a generic model to be developed for the enhancement of ocean anoxia during cooling intervals in the distinctive oceans of the mid-Paleozoic.

## RESULTS

### Progressive oxygen loss from the basin to shelf

**Fig. 2.**
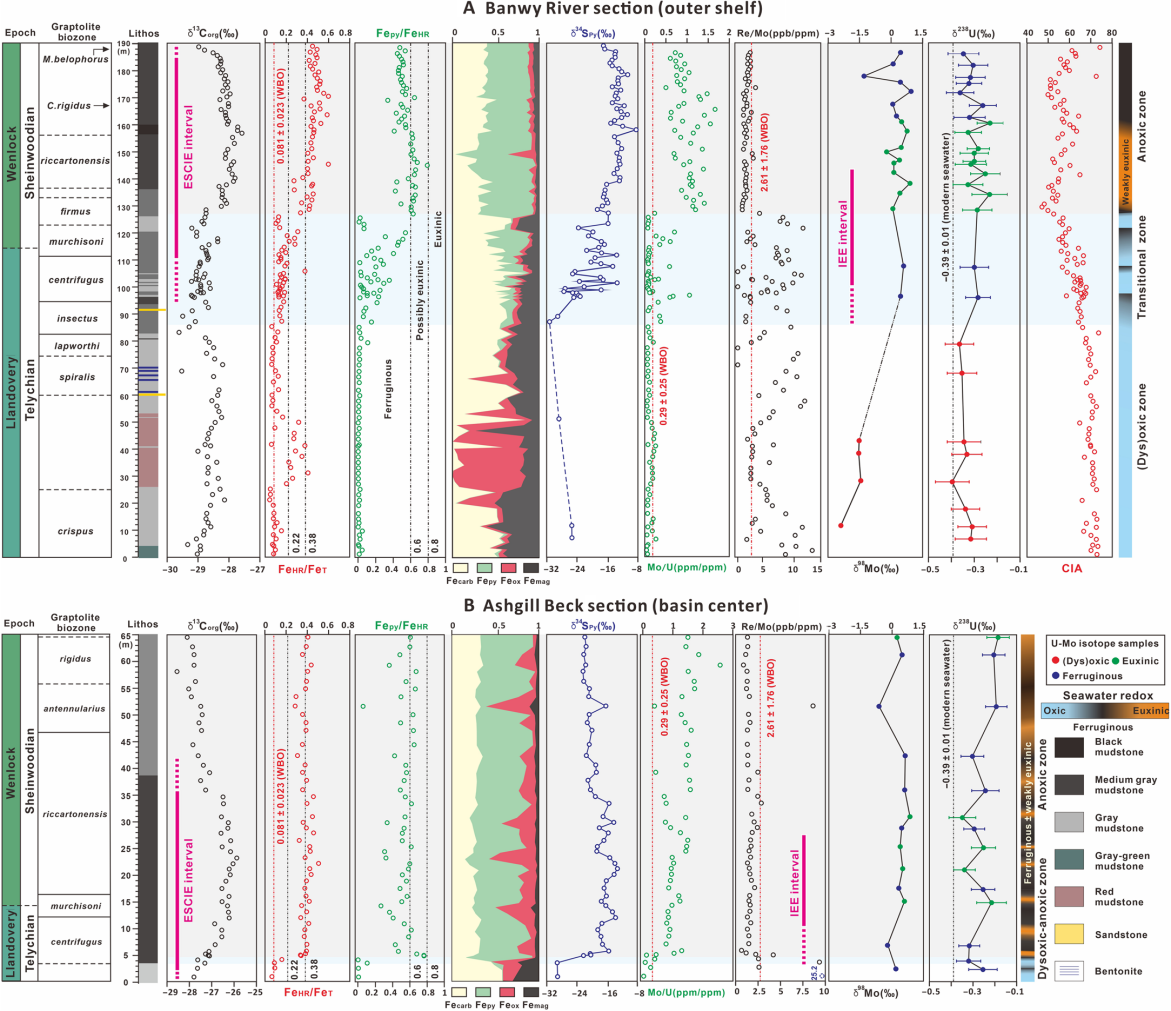
Stratigraphy and redox proxies from the shelf to basin. Stratigraphic and geochemical data for the deep-shelf Banwy River section (**A**) and the basinal Ashgill Beck section (**B**). Graptolite biozones are from published studies (*23*, *51*). The dashed red line on the Fe_HR_/Fe_T_ plot represents the Welsh Basin oxic (WBO) baseline, while dashed lines on the redox-sensitive trace metal plots represent the WBO composition (*3*). On the Fe_HR_/Fe_T_ plot, published thresholds for general identification of oxic (<0.22), possibly anoxic (0.22 to 0.38), and anoxic (>0.38) water column conditions (*24*) are included for context. However, in this case, these thresholds are superseded by our regional oxic baseline calibration (WBO). Dashed lines on the Fe_py_/Fe_HR_ plot represent calibrated thresholds for the identification of ferruginous (<0.6), possibly euxinic (0.6 to 0.8), and euxinic (>0.8) depositional conditions for anoxic samples (*24*, *29*). Pale blue shading indicates the transitional redox zone, while pale gray shading indicates water column anoxia. Uncertainties for Mo-isotope data are smaller than the symbol size. ppb, parts per billion; ppm, parts per million.

Initial insight into the evolution of water column redox conditions is provided by sedimentological constraints. The Banwy River section begins with predominantly light gray mudrocks through much of the Telychian (*crispus* to *lapworthi* biozones), interspersed with a distinct red bed interval (Fig. 2). Dark gray, laminated mudrocks appear in the *insectus* biozone and initially alternate with paler, moderately burrowed mudrocks until a persistent succession of dark, laminated mudrocks develops in the *riccartonensis* biozone (lower Sheinwoodian). This indicates an overall progression to more poorly oxygenated conditions. At Ashgill Beck, where most of the Telychian is not exposed, a transition from pale gray, burrowed mudstones to dark gray, laminated shales occurs abruptly in the upper Telychian around the base of, or within, the *centrifugus* biozone (Fig. 2).

To provide a more nuanced reconstruction of regional water column redox conditions, we use Fe speciation and redox-sensitive trace metal systematics (see Materials and Methods for analytical techniques and the Supplementary Materials for details of the redox proxy framework, as well as all data). Oxic baseline values for Fe speciation and redox-sensitive trace metals have previously been defined for the Welsh Basin [Welsh Basin oxic (WBO)] (*3*), allowing a particularly refined reconstruction of the evolution of ocean redox conditions (see the Supplementary Materials). With the exception of the red bed interval, highly reactive iron–to–total iron (Fe_HR_/Fe_T_) ratios are low throughout most of the Telychian (*crispus* to *insectus* biozones) in the Banwy River section (Fig. 2), suggesting that the deep outer shelf was not anoxic (*24*). This inference is supported by low concentrations (around the WBO value) of redox-sensitive trace elements (U and Mo) (figs. S2 and S3). However, Re/Mo ratios (Fig. 2) are commonly elevated through this interval (again with the exception of the red beds), suggesting that bottom waters may have been at least intermittently dysoxic, rather than fully oxic (see the Supplementary Materials) (*25*).

The red marine mudstones at Banwy River are the local manifestation of a global red bed interval found in deep water to outer-shelf settings during the middle Telychian (*26*–*28*). The red mudstones have higher Fe_HR_/Fe_T_ ratios compared to the interbedded mudrocks because of enrichment in Fe oxides [particularly hematite (Fe_ox_); Fig. 2]. Persistently low U/Al and Mo/Al ratios (fig. S2), coupled with a drop in Re/Mo ratios to WBO values (Fig. 2), imply well-oxygenated bottom water conditions during red bed formation (*25*).

Above this transitional zone, the occurrence of highly elevated Fe_HR_/Fe_T_ and Fe_py_/Fe_HR_ ratios, which persist up until the peak of the ESCIE, suggests the initial development of euxinic water column conditions on the deep shelf (Fig. 2). This inference is supported by increased Mo/U and low Re/Mo ratios, alongside heavy δ^34^S_py_ values (Fig. 2). However, Mo/U ratios are only moderately elevated, likely indicating relatively low concentrations of sulfide in the water column (i.e., weakly euxinic conditions) (*25*). While Fe_HR_/Fe_T_ ratios remain elevated during the falling stage of the ESCIE, there is a distinct decline in Fe_py_/Fe_HR_ ratios to values that suggest a recovery to ferruginous anoxia. This is supported by a subtle decline in δ^34^S_py_ values and Mo/U ratios, as well as an increase in Re/Mo ratios, all of which are consistent with lower levels of sulfide production (*25*).

In the deeper water Ashgill Beck setting, Re/Mo values vary at the bottom of the section (Fig. 2), with some highly elevated values indicating the development of dysoxic conditions in gray mudstones, while samples with lower Re/Mo and elevated U/Al (fig. S2) suggest fluctuations to fully anoxic conditions. However, anoxic gray mudstone samples, as well as anoxic black shales at the base of the overlying strata, have low Fe_HR_/Fe_T_ ratios, suggesting that the sediments were a source of Fe^2+^ to the water column [giving low Fe_HR_/Fe_T_ ratios (*29*)].

The transition to persistently anoxic conditions in the *centrifugus* biozone at Ashgill Beck precedes the development of persistent anoxia observed in the shallower water Banwy River section, where the transition occurs in the later *riccartonensis* biozone [a delay of ~1.5 Myr (million years) according to a recent age model (*30*)] (Fig. 2). Persistently elevated Fe_HR_/Fe_T_ ratios, low Re/Mo ratios, and high δ^34^S_py_ values, combined with variable Fe_py_/Fe_HR_ and Mo/U ratios, suggest alternations between ferruginous and euxinic anoxia (*25*, *29*) in deeper waters throughout the ESCIE interval (Fig. 2).

The redox dynamics evident in deeper and shallower waters suggest that before the development of more persistent, expanded anoxia, the water column was dominantly characterized by dysoxic-anoxic conditions. In deeper waters, this background redox state would have allowed Fe^2+^ to build up in the water column (*29*). The elevated Fe_HR_/Fe_T_ ratios that occur during the red bed interval thus likely reflect water column precipitation of dissolved Fe^2+^, whereby a transient episode of more expansive global oxygenation (see below) resulted in a deepening of the oxycline, which impinged on the deeper ferruginous waters (*29*). The alternative, which is that an enhanced continental weathering influx of Fe oxides drove the global deposition of red beds at this time (*27*, *31*), is not supported by the chemical index of alteration (CIA) record in the River Banwy section, which does not change across the red bed interval (Fig. 2). Specifically, a globally enhanced supply of Fe oxides to the marine realm requires a major increase in chemical weathering intensity on land (*32*, *33*), which should be reflected by higher CIA values. Furthermore, while local depositional factors such as hydraulic sorting and sediment provenance may influence Fe enrichment, such factors would not be expected to occur contemporaneously on a global scale. Later in the Telychian, the oxycline shallowed again, resulting in the reexpansion of dysoxic-ferruginous conditions, before the development of more intense and persistent deeper water anoxia coincident with the onset of the ESCIE, with anoxic conditions then expanding into shallower water settings.

### Constraining global redox conditions

Because of their prolonged residence time [>400 kyr (thousand years)] and distinct redox-driven fractionation effects, U and Mo stable isotopes (δ^238^U and δ^98^Mo) can aid reconstruction of past global oceanic redox conditions (*34*–*36*). We first note that there is no correlation between either U and Al or U and total organic carbon (figs. S7 and S8), suggesting negligible detrital or productivity influence on δ^238^U compositions (*37*), and thus, redox variability was likely the dominant control. There is also no systematic difference between the δ^238^U composition of ferruginous (−0.29 ± 0.05) and weakly euxinic (−0.28 ± 0.05) samples, consistent with the persistence of redox conditions amenable to U(VI)-U(IV) reduction across the sediment-water interface [similar to the Fe(III)-Fe(II) redox couple] (*38*).

Only four oxic samples (from the Banwy River section) contained sufficient Mo for isotopic analysis (Fig. 2), including three oxic samples from the red beds, which have negative δ^98^Mo values (−1.50 ± 0.05‰), and an underlying gray mudstone sample that has a particularly low value (−2.39‰). These red bed data suggest the uptake of Mo to Fe (oxyhydr)oxides precipitating under oxic conditions (*39*), while the very low value in the gray mudstone could reflect either repeated cycles of Fe (and potentially Mn) reduction and reoxidation in pore waters (*39*, *40*) under the fluctuating redox conditions proposed for this interval in the Banwy River section, or nonquantitative Mo-sulfide burial under weakly sulfidic conditions within the sediments. For anoxic samples, δ^98^Mo data show no systematic covariation with either total organic carbon or Al contents (figs. S7 and S8), again indicating that local changes in productivity or detrital influx exerted minimal influence on δ^98^Mo variability. However, the δ^98^Mo values for these samples show considerable variability (Fig. 2), suggesting partial drawdown of Mo under both weakly euxinic and/or ferruginous conditions [where Mo may have been sequestered in sulfidic porewaters or via uptake to Fe (oxyhydr) oxides], rather than complete drawdown of Mo, which requires high water column sulfide concentrations coupled with limited seawater renewal (*41*, *42*).

### Simulating mid-Paleozoic OAE dynamics

**Fig. 3.**
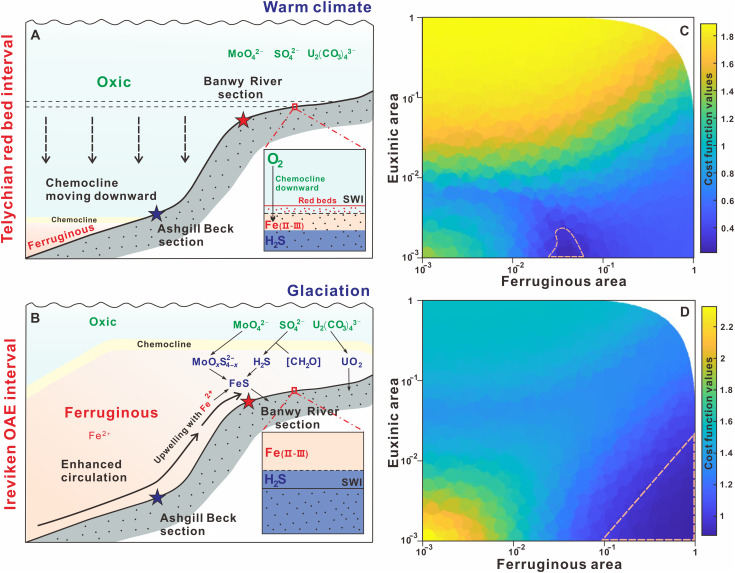
Modeling redox evolution during Silurian anoxia. Schematic showing the evolution of the basinal redox structure and key chemical and physical processes during the Telychian red bed interval (**A**) and the Ireviken OAE interval (**B**). U-Mo isotope mass balance model outputs for the Telychian oxygenation interval (**C**) and the Ireviken OAE interval (**D**). Colors represent the mathematical distance between 100,000 model runs and measured U and Mo isotope values. The areas enclosed by dashed lines represent the most likely redox scenarios.

To provide a quantitative assessment of the seafloor redox landscape during the ESCIE, we applied a modified isotope mass balance model (*43*–*45*) that integrates U and Mo isotope data (Fig. 3 and fig. S10). The model accounts for U and Mo burial under oxic, ferruginous, and euxinic conditions, and in the model runs, we randomly vary the fractions of the seafloor that experience each of these redox regimes to predict the isotopic compositions of U and Mo buried in these areas. We then compare this large suite of model predictions to our isotope data for Mo and U buried under oxic or ferruginous conditions to infer the most likely redox composition of the seafloor throughout the studied time interval. Figure 3 presents these results as a “cost function,” which represents the overall difference between the isotope measurements and the model predictions. Here, the lowest cost function value indicates the most likely seafloor redox landscape and is highlighted by a dashed perimeter line (Fig. 3, C and D; see the Supplementary Materials for further model details).

Because of the lack of Mo isotope data for the non–red bed pre-OAE interval, our focus was on modeling the red bed interval, which appears to represent the maximum extent of pre-OAE oxygenation, as well as the OAE itself (Fig. 3). During the red bed interval, we find that the most plausible scenario to account for the observed U and Mo isotopic compositions involves ~5% of the global seafloor being ferruginous, with less than 1% being euxinic. By contrast, during the onset and aftermath of the ESCIE, the ferruginous seafloor area increased toward 100%, while euxinic areas remained low but may have increased modestly (Fig. 3). This overall degree of deoxygenation substantially exceeds that observed during most Mesozoic OAEs, where anoxia typically bathed less than 10% of the ocean floor (fig. S12) (*15*, *46*).

While we do not specifically use our coupled U-Mo isotope model to quantify the background (i.e., non–red bed) extent of anoxia before the OAE itself, we note that samples deposited in the transitional and anoxic zones (Fig. 2) have similar δ^238^U ranges (−0.28 ± 0.05) relative to the underlying Telychian strata (−0.33 ± 0.04). On a qualitative level, this implies a relatively minor change in the global extent of anoxia (*47*, *48*), as anoxic conditions expanded from the deep ocean into shallower environments, which may be expected given that the deep marine realm (>1000 m) comprises more than 99% of the ocean’s volume and 89% of its seafloor surface (*49*). However, while deoxygenation driven by upwelling ferruginous deep waters may not have significantly expanded the anoxic U sink, the implications of an expansion of anoxia into shallow waters, coupled with the specific development of shallower water euxinia, are particularly profound (see below).

## DISCUSSION

### Cooling-driven mechanism for Paleozoic ocean anoxia

Our interpretation of expansive dysoxic-anoxic conditions in the deep ocean during non–red bed intervals of the mid-Telychian is supported by independent evidence for hypoxic Silurian oceans (*50*). However, the underlying reason for the temporally limited expansion of well-oxygenated conditions during the red bed interval is unclear and requires further study. Nevertheless, our data do provide insight into the factors that controlled the subsequent expansion of anoxia into shallower water environments during the OAE itself.

**Fig. 4.**
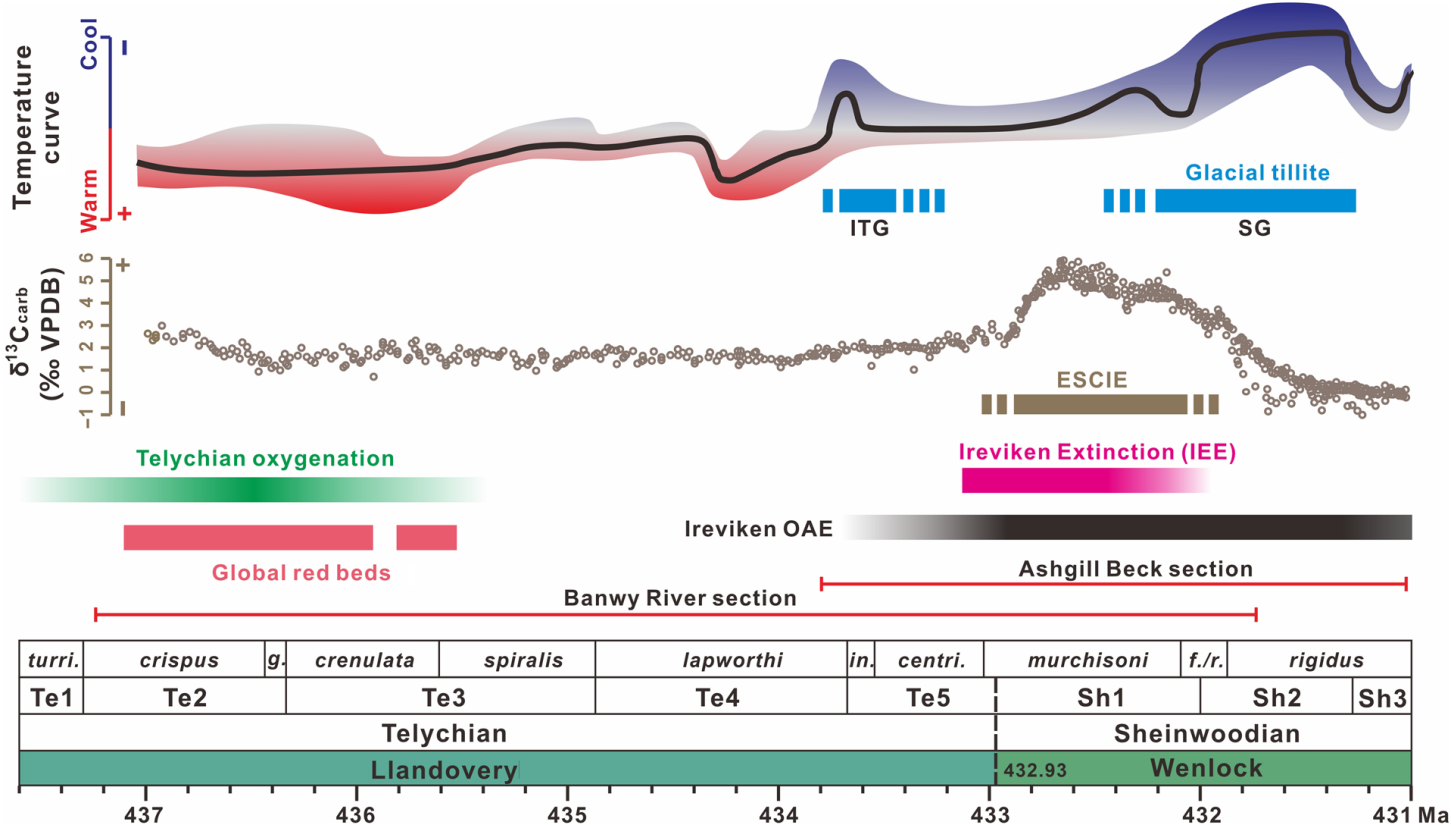
Environmental and redox changes across the Llandovery-Wenlock boundary. Summary of key environmental events and the evolution of ocean redox conditions across the Llandovery-Wenlock boundary in the early Silurian. The δ^13^C_carb_ data are adapted from (*68*), the late Telychian glaciation (lTG) is from (*53*), the mid-Sheinwoodian glaciation (SG) is from (*5*), and the δ^18^O-derived temperature records for Baltica are based on (*16*). All records are calibrated to Geologic Time Scale 2020 (*68*). See the Supplementary Materials for further details of these key environmental records. Abbreviations: *turri.*, *turriculatus*; *g.*, *griestoniensis*; *in.*, *insectus*; *centri.*, *centrifugus*; *f.*, *firmus*; *r.*, *riccartonensis*; VPDB, Vienna Pee Dee belemnite.

Multiple independent lines of evidence (see the Supplementary Materials) support a progressive drop in chemical weathering linked to global cooling, beginning with the late Telychian glaciation (*51*) before the onset of expanded ocean anoxia, which was then followed by the mid-Sheinwoodian glaciation (Fig. 4) (*5*). Consistent with this change, CIA values in the Banwy River section show a decline in chemical weathering intensity, beginning in the late Telychian *spiralis* biozone and reaching a nadir in the Sheinwoodian *firmus* biozone (Fig. 2). This is followed by a return to higher values as chemical weathering increased during the later part of the ESCIE (Fig. 2). In contrast to Mesozoic OAEs (*52*), this suggests that the development of expansive anoxia was linked to a decrease in the chemical weathering influx of key nutrients such as phosphorus, rather than an increase.

We thus propose an OAE mechanism for the mid-Paleozoic in which cooling amplified the temperature contrast between equatorial and high-latitude seawater, intensifying ocean circulation (Fig. 3). Consequent increased downwelling at high latitudes increased the upwelling of ferruginous seawater at lower latitudes, delivering anoxic waters to outer-shelf regions (e.g., the Banwy River section). The upwelling of nutrient-rich deep waters would have stimulated productivity along continental margins, with globally enhanced organic carbon burial occurring as extensive, deoxygenated shelf seas developed (as indicated by peak δ^13^C_org_ values occurring coincident with the development of persistent anoxia on the shelf; Fig. 2). Temperature and sea-level records for the Telychian-Sheinwoodian interval show that the gradual onset of the ESCIE coincides with cooling and glacioeustatic sea-level fall (*20*, *53*). Our model scenario thus suggests that under the relatively low atmospheric oxygen levels of the mid-Paleozoic, a modest increase in the flux of nutrients to the photic zone from upwelling was able to drive expansive shallow water deoxygenation.

These climate-driven weathering dynamics also help to explain the chemistry of anoxic waters during the ESCIE. Relatively enhanced chemical weathering promotes more extensive generation of reactive Fe mineral phases from parent silicate minerals, which ultimately promotes ferruginous anoxia, rather than euxinia (*51*). This is consistent with the development of more widespread euxinia at the peak of the OAE, but as the climatic state recovered (as indicated by an increase in CIA values toward the top of the Banwy River section), more intense weathering promoted more expansive ferruginous anoxia (Fig. 2). This, in turn, would have resulted in more efficient trapping of phosphorus in association with Fe minerals, thereby resulting in a negative productivity feedback that would have limited organic carbon production and burial (*54*, *55*), hence aiding recovery from the expansive OAE and the ESCIE.

Despite the relatively poorly ventilated oceans of the mid-Paleozoic, marine invertebrates thrived and underwent major radiations during both the Ordovician and the recovery interval following the Late Ordovician mass extinction (*56*). By implication, many taxa must have been tolerant of, and able to radiate in, poorly oxygenated oceans (*50*). Despite this innate tolerance, the expansion of anoxic waters into shelf habitats led to the extinction losses of the IEE, and this appears to have been particularly exacerbated by the development of euxinia in shelf settings (*3*). In some regards, the IEE is thus comparable to younger marine extinction crises in which OAEs, and in particular the development of euxinia, are implicated (*57*), but the associated link with transgression and global warming is absent in the Silurian. Instead, global cooling appears to have been the ultimate driver of the major biotic crisis that occurred during the IEE and, by extension, other mid-Paleozoic intervals of anoxia when similar environmental perturbations occurred.

## MATERIALS AND METHODS

### Organic carbon concentrations and isotopes

Field samples were collected from publicly accessible outcrops, and no specific permits were required. For total organic carbon and organic carbon isotope (δ^13^C_org_) analyses, samples were pretreated with 10% hydrochloric acid (HCl) to remove carbonate before analysis in the Cohen Laboratories, University of Leeds. Total organic carbon was determined on a LECO CS-230 analyzer, with replicate analyses of a certified standard (Soil 502–309, *n* = 24) giving a relative standard deviation (RSD) of <3%, with measurements within 2% of certified values.

The δ^13^C_org_ compositions were determined on an Elementar PYRO cube coupled to an IsoPrime continuous flow mass spectrometer. Results are given in δ notation calibrated to the Vienna-Pee Dee Belemnite (VPDB) scale using UREA and sucrose laboratory standards of known isotopic composition [UREA by Merck with δ^13^C = −46.83 ± 0.22‰; Silver Spoon sucrose (commercial) with δ^13^C = −26.19 ± 0.10‰; T&L sucrose (commercial) with δ^13^C = −11.93 ± 0.24‰]. Standard reproducibility given by repeat analyses of the internal sucrose standard was better than 0.1‰ (1 SD).

### Iron speciation and pyrite S isotopes

Unsulfidized iron phases were quantified using a sequential extraction scheme (*58*) in the Cohen Laboratories, University of Leeds. This operationally defined procedure targets Fe present in carbonate phases (Fe_carb_), as Fe (oxyhydr)oxides (Fe_ox_), and in magnetite (Fe_mag_). The Fe_carb_ phase was extracted using Na-acetate solution at pH 4.5 and 50°C for 48 hours. The residue was then treated with Na-dithionate for 2 hours at room temperature to extract Fe_ox_. Last, Fe_mag_ was extracted with ammonium oxalate solution for 6 hours at room temperature. All extractant solutions were then measured for Fe by atomic absorption spectrometry. Sulfide-bound Fe, including acid volatile sulfide Fe (Fe_AVS_, below detection in all cases), and pyrite (Fe_py_) were extracted by the two-step HCl and chromous chloride (CrCl_2_) method (*59*). The released H_2_S was precipitated as Ag_2_S, which was then determined gravimetrically. Replicate analyses (*n* = 8) of the international reference material, WHIT (*60*), gave RSDs of <5% of all Fe phases.

Sulfur isotope (δ^34^S_py_) analyses were performed on the Ag_2_S precipitates using an Elementar PYRO cube coupled to an IsoPrime continuous flow mass spectrometer in the Cohen Laboratories, University of Leeds. Calibration to the Vienna-Canyon Diablo Troilite scale was performed using a barium sulfate standard, SWS-3A (assigned δ^34^S = 20.3‰), and an interlab standard, CP-1 (chalcopyrite; assigned δ^34^S = −4.56‰), validated against internationally recognized reference materials NBS-127 (20.3‰), NBS-123 (17.01‰), IAEA S-1 (−0.30‰), and IAEA S-3 (−32.06‰). Precision was verified through repeat measurements of the CP-1 standard, yielding a value of ±0.15‰ (1 SD).

### Major and trace elements

Samples were initially ashed at 550°C for 8 hours, followed by dissolution with a mixture of HNO_3_, HF, and HClO_4_. After evaporation to dryness, samples were treated with boric acid (H_3_BO_3_) to ensure full solubilization of Al hexafluorates and heated to dryness before being redissolved in hot HNO_3_. Total element concentrations were subsequently determined using inductively coupled plasma optical emission spectrometry (Thermo Fisher Scientific iCAP 7400) for major elements (Al, Fe, and Mn) and inductively coupled plasma mass spectrometry (Thermo Fisher Scientific iCAPQc) for trace elements (U, Mo, and Re). Replicate extractions of international sediment standard SGR-1 yielded RSDs of <5% for all elements of interest, and analyses were within 3% of certified values.

### Uranium and molybdenum isotopes

Uranium and Mo isotope analyses were performed in an ISO6 metal-free clean laboratory at Royal Holloway, University of London. Samples were precisely weighed (to give more than ~300 ng of Mo and ~100 ng of U) and combined with an aliquot of either a ^97^Mo-^100^Mo double spike or an IRMM 3636a ^236^U-^233^U double spike to give a Mo spike/sample ratio of ∼0.3 and a U spike/sample ratio of ∼0.1. Samples were then digested with a concentrated mixture of HNO_3_ and HCl (in a 3:1 ratio) at 150°C to dissolve the nondetrital fraction. Mo and U were purified from the sample matrix following chromatography protocols using AG1-X8 200–400 dry mesh resin (*61*, *62*) and Eichrom UTEVA resin (*63*). Both isotopes were measured on a Thermo-Finnigan Neptune Plus multicollector inductively coupled plasma mass spectrometer equipped with a CETAC Aridus III desolvating nebulizer system for sample introduction. An acid blank was measured before each sample to correct for memory effects during sample washout. Isotope compositions were calculated relative to NIST 3134 for Mo and the CRM112a standard for U.

δ98Mo (‰)=[(98Mosample/95Mosample)/(98Mo/95MoNIST3134)−1]×1000+0.25 (1)

δ238U (‰)=[(238Usample/235Usample)/(238U/235UCRM112a)−1]×1000 (2)

Replicate measurements of the Open University standard relative to NIST 3134 yielded a mean value of −0.36 ± 0.05‰ (2 SD; *n* = 6), with results being within the uncertainty of the reported value of −0.37‰ (*64*). Replicate measurements of certified SDO-1 and SGR-1 standards yielded values of 1.03 ± 0.05‰ (2 SD, *n* = 4) and 0.66 ± 0.10‰ (2 SD, *n* = 4), respectively, which are similar to published values of 1.05 ± 0.14‰ (*64*) and 0.68 ± 0.05‰ (*65*). For δ^238^U, repeat measurements of the SDO-1 and SGR-1 standards yielded values of −0.08 ± 0.08‰ (2 SD, *n* = 4) and −0.22 ± 0.09‰ (2 SD, *n* = 4), respectively, which are similar to previous study values of −0.07 ± 0.03‰ (*66*) and −0.17 ± 0.02‰ (*66*). Total procedural blanks measured by isotope dilution were negligible for both isotopes.

### Joint U-Mo mass balance model

We used an updated dynamic mass balance model (*44*) for U and Mo cycling in the global ocean (see parameters in table S6), where the integrated input and output fluxes control the seawater inventory and isotopic composition of both elements

$$\frac{d{\left[M\right]}_{\text{sw}}}{\text{dt}}=F_{\text{input}}-F_{\text{output}}$$ (3)

$$\frac{d{\left[M\right]}_{\text{sw}}}{\text{dt}}\cdot \delta M_{\text{sw}}=F_{\text{input}}\cdot \delta_{\text{input}}-F_{\text{output}}\cdot \delta_{\text{output}}$$ (4)

The [M]_sw_ and δM_sw_ parameters denote the seawater concentration and isotopic composition of a specific metal (U and Mo), respectively, while “F” represents the flux. Simplifying the input to river sources and the output to euxinic, ferruginous, and (dys)oxic sinks for both elements, we can express the mass balance equations as

$$\frac{d{\left[M\right]}_{\text{sw}}}{\text{dt}}=F_{\text{river}}-\sum F_{i}$$ (5)

$$\frac{d{\left[M\right]}_{\text{sw}}}{\text{dt}}\cdot \delta M_{\text{sw}}=F_{\text{river}}\cdot \delta_{\text{river}}-\sum F_{i}\cdot \delta_{i}$$ (6)

$$\delta_{i}=\delta_{\text{sw}}+\Delta_{i}$$ (7)

where F_i_ represents each redox sink, and the sediment isotope (δ_i_) composition is derived from the seawater isotopic value and the fractionation between sediment and seawater (expressed as Δ) in different redox settings.

Fluxes into sediments can vary in magnitude on the basis of several factors, including the areal seafloor extent of the specific redox environment, oceanic elemental concentrations, and the concept of “offshore scaling.” This scaling factor assumes that euxinic and ferruginous sinks necessitate progressively larger seafloor areas as they expand into regions with lower organic carbon fluxes (*43*)

$$F_{i}=F_{i0}\cdot \left(\frac{A_{i}}{A_{i0}}\right)\cdot \left(\frac{\left[M\right]}{{\left[M\right]}_{0}}\right)\cdot {\text{OSS}}_{i}$$ (8)

where A_i_ represents the areal fraction of each redox sink, with the subscript “0” denoting present-day values, and OSS_i_ indicates the offshore scaling factor for ferruginous and euxinic sinks.

Two sets of average δ^98^Mo and δ^238^U values, obtained from oxic red bed intervals and the Ireviken OAE interval, were input into the model. The model was then run 100,000 times, from present-day initialization under random choices of oxic, reducing, and euxinic areal fractions, to investigate the evolution of the areal extent of different redox conditions at the steady state under the initial conditions outlined in table S6.

Cost function is a mathematical tool used to quantify the discrepancy between model predictions and observed data, which is generally defined as

$$J\left(\theta_{0},\theta_{1}\right)=\frac{1}{2m}\sum_{i=1}^{m}{\left[h_{\theta }\left(x^{i}\right)-y^{i}\right]}^{2}$$ (9)

The $h_{\theta }\left(x^{i}\right)$ and y^i^ parameters are model-predicted values and observed values over *m* training examples. θ_0_ and θ_1_ are model parameters, x^i^ represents the *i*-th data point, and $h_{\theta }\left(x_{i}\right)=\theta_{0}+\theta_{1}x_{i}$ is the hypothesis function. Minimizing $J\left(\theta_{0},\theta_{1}\right)$ ensures that the model achieves the best fit by optimizing the intercept (θ_0_) and slope (θ_1_) to reduce the prediction error. To quantitatively evaluate the agreement between modeled and observed isotopic compositions, we use the cost function here as

$$J\left(\theta_{0},\theta_{1}\right)=\frac{1}{2m}\sum_{i=1}^{m}\left[{\left(\delta^{98}{\text{Mo}}_{\text{model},i}-\delta^{98}{\text{Mo}}_{\text{obs},i}\right)}^{2}+{\left(\delta^{238}U_{\text{model},i}-\delta^{238}U_{\text{obs},i}\right)}^{2}\right]$$ (10)

The cost function J represents the mean squared error between the modeled and observed isotopic compositions. The terms δ^98^Mo_(model,i)_ and δ^238^U_(model,i)_ denote the modeled Mo and U isotopic compositions for the i-th simulation, respectively. Similarly, δ^98^Mo_(obs,i)_ and δ^238^U_(obs,i)_ represent the observed isotopic compositions for the corresponding sample. For this study, δ^98^Mo_(obs,i)_ and δ^238^U_(obs,i)_ were defined as constant values. For the lower part of the Banwy River section, which reflects marine redox conditions during the mid-Telychian oxygenation interval (Fig. 3), these values are set to the averages of the three samples: δ^98^Mo = −1.50‰ and δ^238^U = −0.36‰. For samples from the Sheinwoodian ESCIE interval in both sections (Fig. 3), the average isotopic compositions are δ^98^Mo = 0.35‰ and δ^238^U = −0.28‰. The cost function J was minimized over 100,000 iterations, exploring combinations of redox-sensitive parameters to simulate the isotopic response under steady-state conditions. This approach enables the model to produce plausible outputs related to the spatial extent of different marine redox conditions during the studied interval.

## Supplementary Materials

### The PDF file includes:

Supplementary Text Figs. S1 to S12 Tables S1 to S6 References

### Other Supplementary Material for this manuscript includes the following:

Code S1
