# Supplementary material for: Cooling-induced intensification of ocean anoxia in the mid-Paleozoic
Source: Sci Adv. 2026 Mar 13;12(11):eaec8573. doi: 10.1126/sciadv.aec8573 (PMC12985696; doi:10.1126/sciadv.aec8573)
Supplement: Supplementary file 1 — Supplementary Text Figs. S1 to S12 Tables S1 to S6 References [file sciadv.aec8573_sm.pdf]

Science Advances
AAAS

# Supplementary Materials for

## Cooling-induced intensification of ocean anoxia in the mid-Paleozoic

Yuxuan Wang *et al.*

Corresponding author: Yuxuan Wang, wyxleeds@gmail.com; Paul B. Wignall, p.b.wignall@leeds.ac.uk; Simon W. Poulton, s.poulton@leeds.ac.uk

*Sci. Adv.* **12**, eaec8573 (2026)
DOI: 10.1126/sciadv.aec8573

**The PDF file includes:**

Supplementary Text
Figs. S1 to S12
Tables S1 to S6
References

**Other Supplementary Material for this manuscript includes the following:**

Code S1

**Supplementary Text**

**Geological background and sample description**

*The Ireviken extinction event (IEE)*

The mid-Silurian Ireviken Extinction Event (IEE) straddles the Telychian and Sheinwoodian Stage boundary, and lasted more than 0.7 Ma (Fig. 4). A significant conodont extinction in the late Telychian *(5,69)* saw the loss of 48 out of 60 pandemic species, representing an extinction rate of 80%, and conodont diversity did not fully recover for the remainder of the Silurian *(70)*. A major phase of graptolite decline, with species diversity declining from 21 to 4, occurred in the early Sheinwoodian *(2,71)*. Other marine taxa, including chitinozoans, ostracods, corals and trilobites, also experienced significant crises to varying extents at various times *(1)*.

*The early Sheinwoodian carbon isotope (ESCIE)*

Although the magnitude of carbon isotope excursions varies and may be influenced by local or regional depositional conditions, the Early Sheinwoodian Carbon Isotope Excursion (ESCIE) has been globally documented across diverse sedimentary facies from different paleocontinents *(5,72)* (Fig. S1A). Most ESCIE records are derived from shallow-water carbonate platforms, with the highest $\delta^{13}C_{carb}$ values of 6.6‰ being reported from Norway *(73)*, with ~5.5‰ peaks in New York State and Ontario *(74)*, 5‰ in Sweden *(17)* (Gotland), 4‰ in Oklahoma, and 3‰ in Nevada *(75)*. In stratigraphic sections with well-defined graptolite biostratigraphy, such as the Baltic cores *(25,76)* and the Aizpute-41 section in Latvia *(18)*, the onset of the ESCIE occurs in the late *murchisoni* Biozone in the later stages or slightly after the IEE *(5)*. Peak $\delta^{13}C_{carb}$ values are observed within the *riccartonensis* Biozone or slightly higher. In deeper-water graptolitic facies of the Baltoscandian Basin, the $\delta^{13}C$ excursion begins in the upper *murchisoni* Biozone and concludes in the Middle

Wenlock *(77)*. Notably, the peak interval of the ESCIE shows a significant temporal offset from the main phase of the IEE.

In the UK, the Early Sheinwoodian Carbon Isotope Excursion (ESCIE) is well-documented across different depositional settings, from the inner shelf Midland carbonate platform (Eastnor Park and Lower Hill Farm boreholes *(77)*) to the mid and outer clastic shelf (Buttington section *(79)* and Banwy River section *(25)*, respectively) and into the deep basin at the Ashgill Beck section (Fig. S1B). On the shallow Midland carbonate platform, the EP and LHM boreholes record a maximum $\delta^{13}C_{carb}$ increase of over 4‰, initiating in the early Sheinwoodian (Fig. S1C).

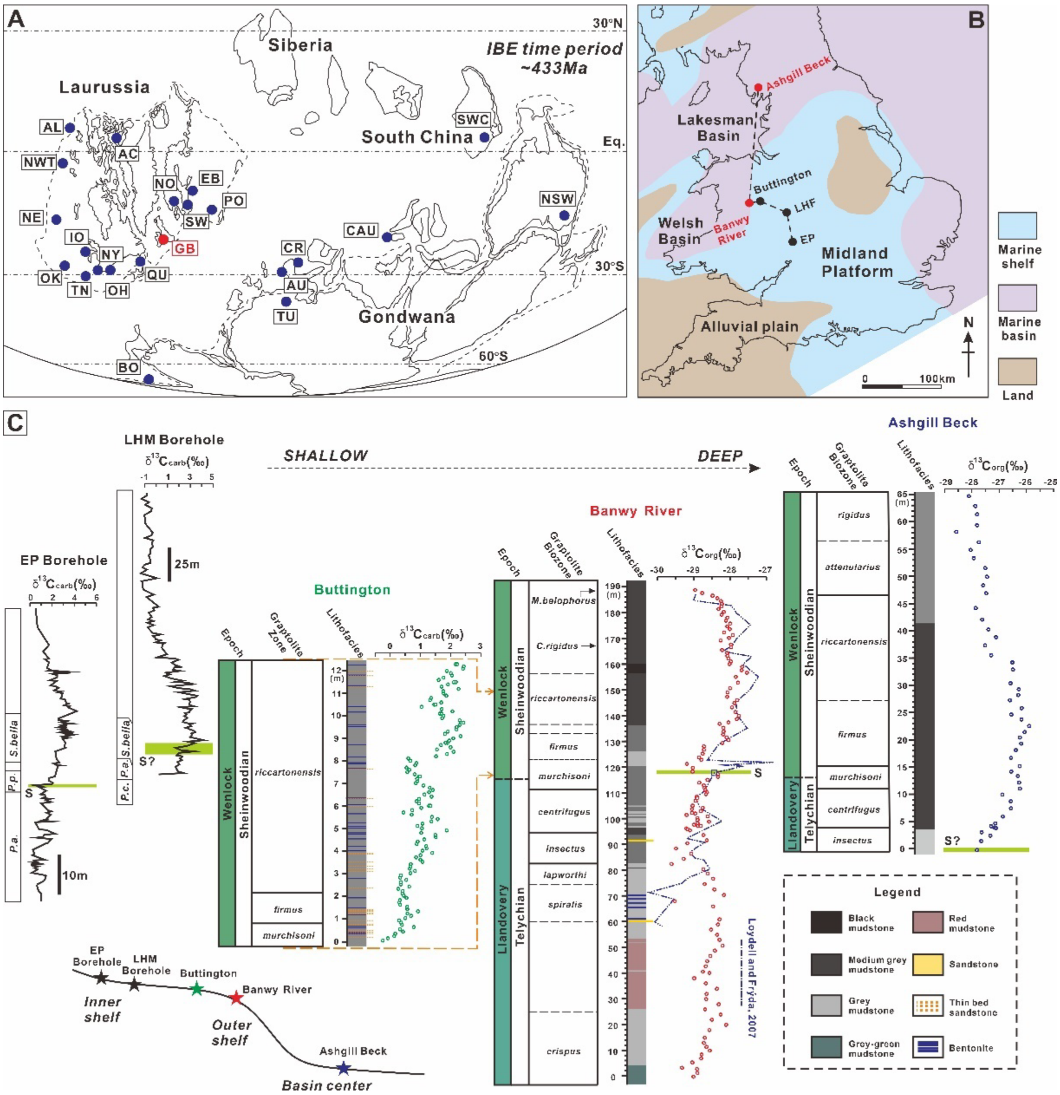


**Figure S1:** A. Global paleogeography of the late Llandovery, Silurian, showing locations where the Ireviken Extinction Event (IEE) or Early Sheinwoodian Carbon Isotope Excursion (ESCIE) have been documented (after *(5)*). Abbreviations: Alaska (AL), Arctic Canada (AC), Austria (AU), Bolivia (BO), Greater Caucasus region (CAU), Czech Republic (CR), East Baltic (EB), Iowa (IO), Nevada (NE), New South Wales (NSW), New York & Ontario (NY), North West Territories (NWT), Norway (NO), Ohio (OH), Oklahoma (OK), Podolia (PO), Quebec (QU), South West China (SWC), Sweden (SW), Tennessee (TN), Tunisia (TU). Our study area (Great Britain) is highlighted in red. B. Sites where the Ireviken event is recorded in England and Wales, and palaeogeographic reconstruction of the depositional environment during the mid-Silurian, prepared by the authors based on published geological and palaeogeographic frameworks. Carbonate platform boreholes (LHF = Lower Hill Farm, EP = Eastnor Park) are from (77). C. Carbon isotope stratigraphy and biozones from the Welsh Basin, Lakesman Basin, and the adjacent Midland Platform. Carbonate carbon isotope ($\delta^{13}C_{carb}$) data for the inner-shelf LHF and EP boreholes are from *(77)*. The $\delta^{13}C_{carb}$ data for the mid-shelf Buttington section are from *(78)*. Organic carbon isotope ($\delta^{13}C_{org}$) data for the outer-shelf Banwy River section and the basin-center Ashgill Beck section are from this study, and the blue dashed line represents the lower resolution data of ref.[77]. Abbreviations: S - start of the ESCIE; *P.a.* - *Pterospathodus amorphognathoides* Biozone; *P.c.* - *Pterospathodus celloni* Biozone; *P.p.* - *Pterospathodus procerus* Biozone *(77)*.

Banwy River Section

The Banwy River section in Wales comprises a continuous, mudstone-dominated succession spanning much of the Llandovery to lower Wenlock interval, from green mudstones in the Telychian *crispus* Biozone to black mudstones in the Sheinwoodian *rigidus* Biozone *(22)* (Fig. S2). Bioturbated layers are present in the grey and medium-grey mudstones of the Telychian and lowermost Sheinwoodian strata, but are absent in the upper Sheinwoodian, which consists solely of finely laminated black mudstones (Fig. S2). Red mudstones occur in the lower section between the *crispus* and *spiralis* graptolite biozones. Thin bentonites occur within the *spiralis* Biozone. We logged the ~190 m section and collected 139 mudstone samples for geochemical analysis (see Tables S1 and S2).

The Banwy River section has a well-constrained graptolite biostratigraphy *(22)* and existing $\delta^{13}C_{carb}$ and $\delta^{13}C_{org}$ records *(4,25)*. Previously sampled at moderate stratigraphic resolution, the $\delta^{13}C_{carb}$ record has previously been utilized *(4)* (Fig. S1C, blue dashed line) to identify the ESCIE onset. Our high-resolution $\delta^{13}C_{org}$ data align closely previous results *(25)*, but provide a more detailed and smoother trend, capturing a maximum $\delta^{13}C_{org}$ increase of ~2‰. Notably, a small negative $\delta^{13}C_{org}$ excursion (~0.7‰) occurs in the *insectus* Biozone, predating the ESCIE (Fig. S1C). This feature corresponds closely with another similar, high-resolution, negative $\delta^{13}C_{org}$ excursion recorded in the Altajme borehole, Gotland, Sweden, immediately before the onset of ESCIE *(13)*. This consistent decoupling of $\delta^{13}C_{org}$ and $\delta^{13}C_{carb}$ in different basins may indicate a perturbation to the global carbon cycle, potentially associated with elevated productivity (*13,82)*. At Banwy River, the ESCIE terminates in the mid-Sheinwoodian *belophorus* Biozone *(25)*.

*Ashgill Beck Section*

The Ashgill Beck section, located in the southern Lake District, UK, consists of deep-water mudstone facies that transition from the Browgill Formation of the Stockdale Group to the Brathay Formation of the Tranearth Group *(83)* (Figs. S1, S2). Graptolite biostratigraphy in this section *(23)* begins with the *centrifugus* Biozone and ranges up to the upper Sheinwoodian. We logged 65 m of strata from the uppermost Telychian and into the lower Sheinwoodian, and collected 50 mudstone samples for geochemical analysis (Tables S3 and S4). In this section, a positive $\delta^{13}C_{org}$ excursion occurs, which exceeds 2‰, beginning at the base of the section before declining in the upper *riccartonensis* Biozone.

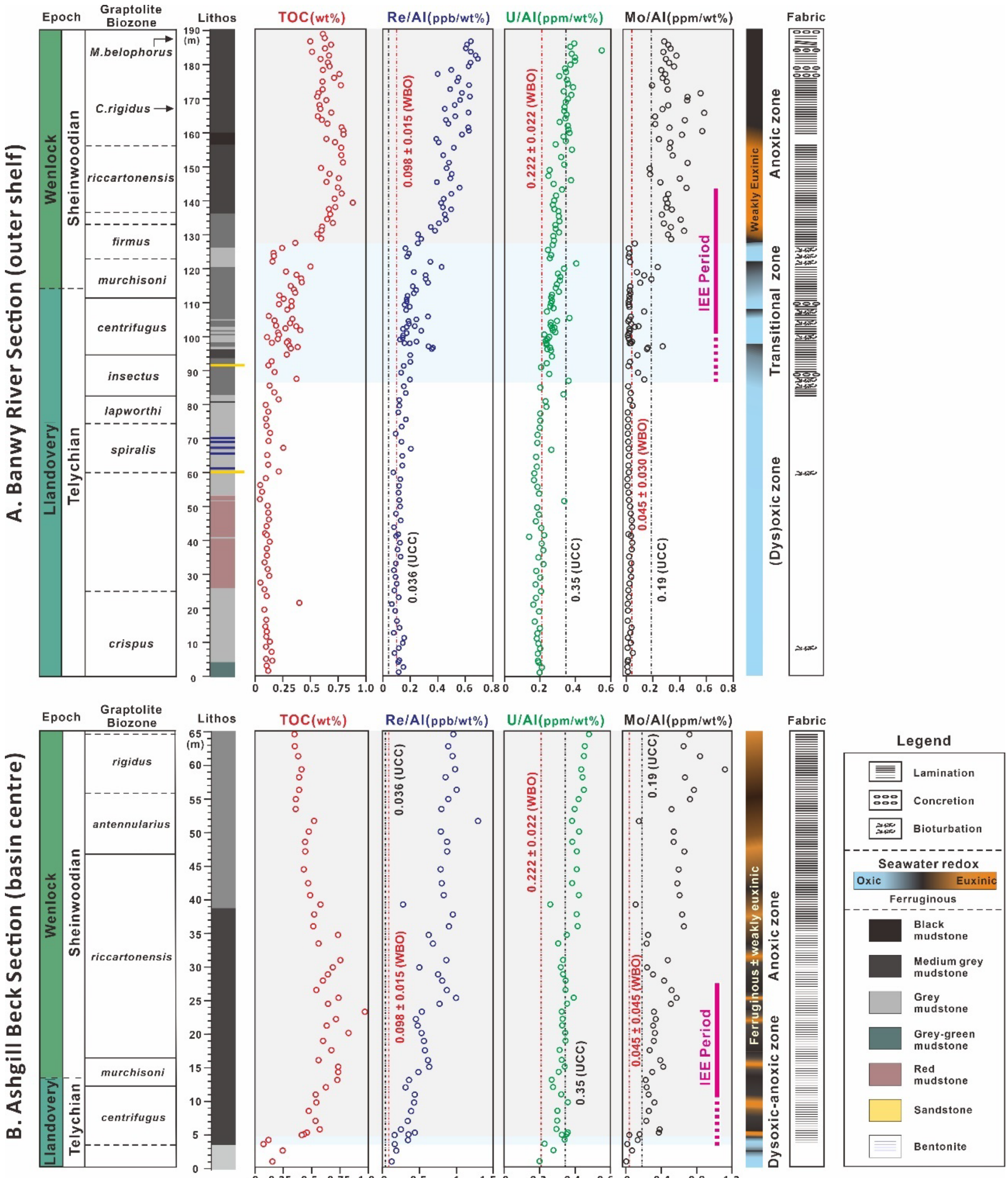


**Figure S2:** Summary of graptolite biostratigraphy, sedimentological fabrics and geochemical data (TOC, Re/Al, U/Al and Mo/Al) from the deep-shelf Banwy River section (A) and the basinal Ashgill Beck section (B). Graptolite zones are from *(22,23)*. Red dotted lines on the Re/Al, U/Al and Mo/Al plots represent the Welsh Basin oxic baselines (WBO) for the mid-Silurian *(3)*, while the black dotted lines represent average Upper Continental Crust values (Mo and U are from *(80)*; Re is from *(22)*).

**Framework for regional redox interpretation**

To reconstruct water column redox conditions, we employed independent inorganic redox proxies based on Fe speciation and redox-sensitive trace metal (RSTM) concentrations (see Tables S1 to S4). Iron speciation focuses on the ratio of highly reactive Fe ($Fe_{HR}$) to total Fe ($Fe_T$) *(27,31,60)*. Extensive calibration in both modern and ancient settings (which by definition considers the effects of diagenesis *(84)*) has established general thresholds to differentiate oxic and anoxic depositional conditions *(26,35,60,62,85,86,87,88)*. Oxic water column conditions are generally indicated by $Fe_{HR}/Fe_T$ <0.22, while ratios >0.38 suggest anoxic conditions, reflecting additional precipitation of $Fe_{HR}$ phases in the water column *(26)*. Intermediate $Fe_{HR}/Fe_T$ values (0.22-0.38) are considered equivocal and require supplementary evidence to clarify redox conditions *(26)*.

For anoxic samples, the $Fe_{py}/Fe_{HR}$ ratio can be used to distinguish between euxinic and ferruginous conditions. Ratios above 0.6–0.8 typically indicate euxinia *(89-91)*, while ratios <0.6 suggest ferruginous conditions *(26,62)*. Unlike $Fe_{HR}/Fe_T$ ratios, $Fe_{py}/Fe_{HR}$ ratios cannot be calibrated using local oxic samples to establish basin-specific thresholds for anoxic settings *(3)* (see below). Therefore, to support our interpretation of $Fe_{py}/Fe_{HR}$ ratios based on the established thresholds, we incorporate independent evidence from RSTM systematics.

Uranium (U) and molybdenum (Mo) are highly soluble and exhibit limited enrichment in oxic sediments *(92)*. In oxic seawater, U predominantly exists as U(VI) in uranyl carbonate complexes ($UO_2(CO_3)_3^{4-}$) *(93)*, while Mo is stable as Mo(VI) in the molybdate oxyanion ($MoO_4^{2-}$) *(94)*. Under anoxic conditions at the sediment–water interface, soluble U(VI) is reduced to insoluble U(IV), primarily at the Fe(II)–Fe(III) redox boundary *(40)*. By contrast, Mo removal requires the presence of elevated concentrations of $HS^-$ in the water column, forming thiomolybdates ($MoO_xS_{4-x}$) under

euxinic conditions *(95,96)*.

Rhenium (Re), by comparison, is enriched just below the sediment–water interface (~1 cm) even under weakly reducing (dysoxic) conditions, where U and Mo enrichment is absent *(97)*. Thus, combined consideration of Re, U and Mo systematics, alongside Fe speciation and Mo/U and Re/Mo ratios, may allow dysoxic, anoxic non-sulphidic (ferruginous) and euxinic conditions to be distinguished at the basin scale *(27)*.

**Defining regional oxic baseline values**

Recent studies have underscored the importance of calibrating redox interpretations for the specific site of interest, as general redox thresholds are often inappropriate at local or regional scales *(3,31,98-100)*. This is clearly illustrated in the Welsh Basin, where samples from the deepest water section at Borth exhibit $Fe_{HR}/Fe_T$ ratios well below the general oxic baseline value (~0.22; *(26,35)*), while U/Al and Mo/Al ratios are considerably lower than upper continental crust (UCC) values, all supporting oxic depositional conditions *(3)* (Fig. S3). We thus follow a previous approach *(3)* and utilize Borth samples to establish a regional oxic $Fe_{HR}/Fe_T$ range for the Silurian Welsh Basin of 0.081 ± 0.023 (Figs. 2, S3, S4).

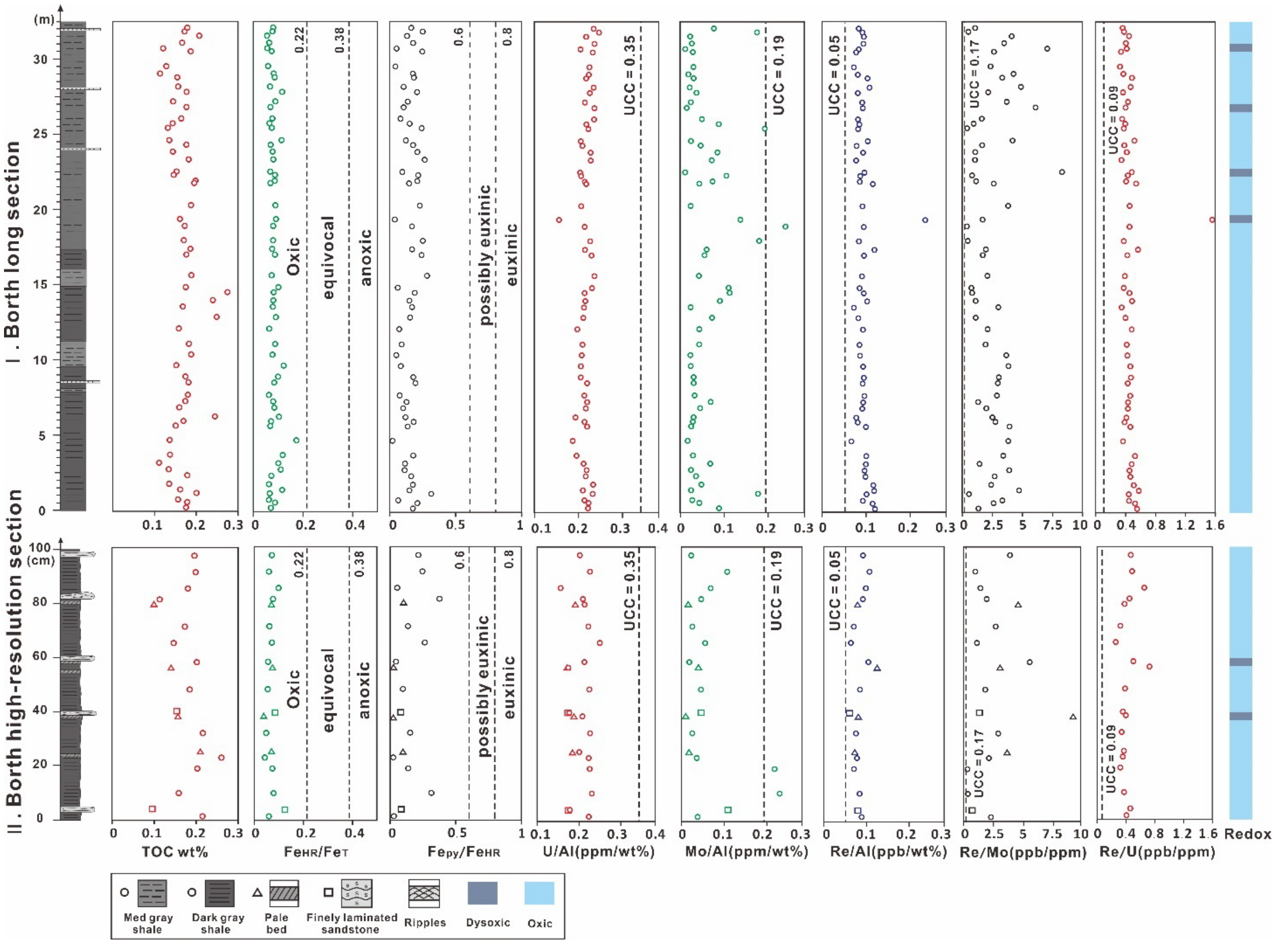


**Figure S3:** Geochemical data for two Borth sections (data from the *(99)*). For plots involving Re, we calculate ratios relative to the revised crustal average value of 0.29 ppb *(81)*, while crustal averages for Mo, U and Al are from the *(80)*. Possible dysoxic intervals are identified by elevated Re/Mo and/or Re/U ratios *(27)*.

Low U and Mo contents support oxic or dysoxic depositional conditions at the sediment–water interface at Borth *(27,100)*, which we then distinguish utilizing Re systematics. Relatively stable Re/Al ratios that are only slightly above UCC values (~0.036) (Fig. S3), suggest limited drawdown of Re. Furthermore, Re/Mo and Re/U ratios generally fall in the range typical for modern sediments deposited under oxic conditions *(27)* (Fig. S4). These characteristics suggest that, with a few minor exceptions (Fig. S3), the dysoxic conditions required for Re drawdown *(27,97,101)* were largely confined to sediment porewaters during diagenesis. Thus, similar to Fe speciation, we use WBO values *(3)* to define oxic thresholds for Re, U and Mo (Fig. 2). Therefore, non-sulphidic (i.e., ferruginous) water column anoxia is indicated by increased U/Al ratios with low Re/Mo ratios, while euxinia is identified by high Mo/U and very low Re/Mo ratios, alongside high U/Al ratios *(27)*.

Figure S4 shows that there is a high degree of consistency between independent redox proxy data. Specifically, low $Fe_{HR}/Fe_T$ ratios coincide with low U/Al ratios (i.e., where both parameters are lower than the WBO value), supporting (dys)oxic depositional conditions. There is then a general trend of increasing $Fe_{HR}/Fe_T$ and U/Al ratios, suggesting a progressive increase in the intensity or persistence of anoxia *(102, 103)*. Similarly, Mo/Al ratios remain low while $Fe_{py}/Fe_{HR}$ ratios initially increase (Fig. S4), supporting diagenetic sulphide production, where $Fe_{HR}$ phases were progressively sulphidized and Mo drawdown was limited due to the lack of water column euxinia or sulphide generation close to the sediment-water interface. However, as sulphide became more abundant at the sediment-water interface and in the overlying water column, Mo drawdown progressively increased, giving elevated Mo/Al and $Fe_{py}/Fe_{HR}$ ratios *(102, 103)*.

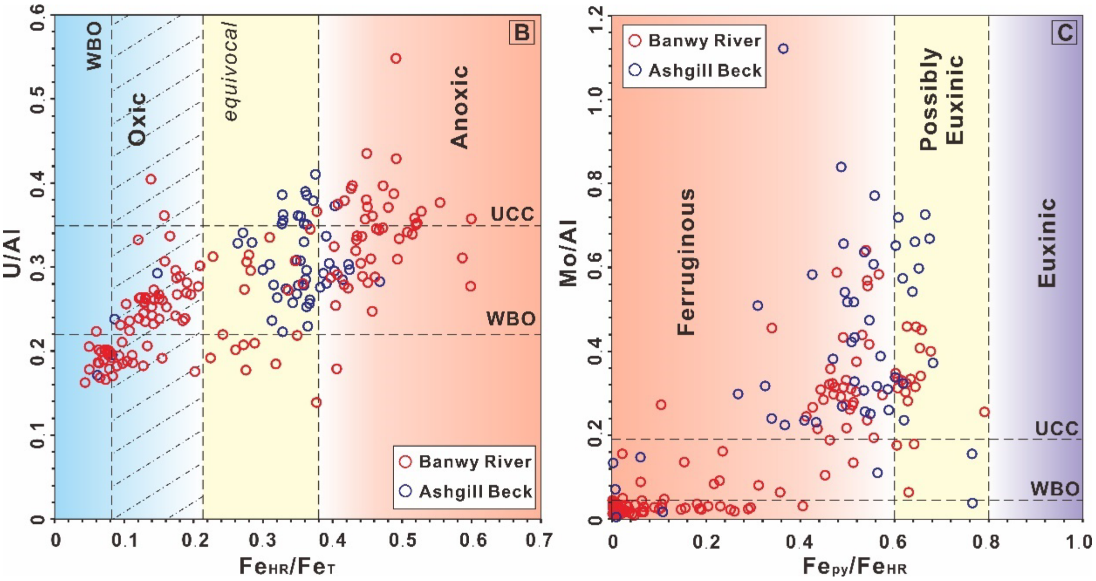


**Figure S4:** Crossplots showing Fe speciation data, and U/Al and Mo/Al ratios in Telychian-Sheinwoodian sedimentary rocks from the Welsh Basin (Banwy River) and Lakesman Basin (Ashgill Beck). A. U/Al versus $Fe_{HR}/Fe_T$. The horizontal dashed lines represent the average WBO U/Al value *(3)* and the UCC value *(80)*. B. Mo/Al versus $Fe_{Py}/Fe_{HR}$. The horizontal dashed lines represent the average WBO Mo/Al value *(3)* and the UCC value *(80)*.

**Redox zones in the study area**

The regional water column redox evolution of the study basins can be delineated into six distinct types (Figs. S5 and S6):

**i)** Oxic samples from Borth:

These samples, serving as the primary reference for the WBO line, were dominantly deposited under oxic water column conditions and are characterized by low $Fe_{HR}/Fe_{T}$ and RSTM ratios (Figs. S3, S5, S6).

**ii)** Red bed samples from the oxic zone of Banwy River:

Collected from 26–53 m height in the section (between the *crispus* and *spiralis* graptolite biozones), these samples exhibit higher $Fe_{HR}/Fe_{T}$ ratios relative to other oxic-dysoxic samples (Fig. S5). This records an environment with a persistent flux of dissolved $Fe^{2+}$, which was oxidized in the water column, resulting in sediment enrichment in $Fe_{HR}$ phases, particularly hematite (Fig. 2; Table S1). Redox sensitive trace metal ratios are persistently very low, due to the well-oxygenated conditions both in the water column and during early diagenesis close to the sediment-water interface (Fig. S6).

**iii)** Samples from the (dys)oxic zone of Banwy River (excluding red bed samples):

These samples have low $Fe_{HR}/Fe_{T}$, U/Al and Mo/Al ratios (Fig. 2), suggesting that conditions were not anoxic. However, the occurrence of elevated Re/Al and some high Re/Mo ratios indicates at least the temporal development of dysoxic conditions, promoting Re drawdown *(97,101)* (Figs. 2, S6).

**iv)** Transitional samples from the Banwy River and Ashgill Beck Sections:

Characterized by somewhat elevated $Fe_{HR}/Fe_{T}$ and $Fe_{py}/Fe_{HR}$ ratios, and high Re/Al and U/Al

ratios, along with fluctuating Re/Mo values, these samples indicate progressive oxygen depletion relative to the samples noted above, and a water column experiencing fluctuating redox conditions between dysoxic and ferruginous (Figs. 2, S5, S6).

**v)** Ferruginous anoxic samples from Banwy River and Ashgill Beck:

These samples show markedly elevated $Fe_{HR}/Fe_T$, Re/Al, U/Al and Mo/Al ratios, accompanied by a substantial decrease in Re/Mo ratios and $Fe_{py}/Fe_{HR}$ values below the threshold of 0.6, indicative of an anoxic ferruginous environment *(49)* (Figs. 2, S5, S6).

**vi)** Weakly euxinic samples from Banwy River and Ashgill Beck:

These samples exhibit significantly elevated $Fe_{HR}/Fe_T$, Re/Al, U/Al and Mo/Al ratios. However, they are distinguished by the lowest Re/Mo ratios, with $Fe_{py}/Fe_{HR}$ values that are above the 0.6 threshold but below 0.8, suggesting a weakly euxinic depositional environment (Figs. S5 and S6).

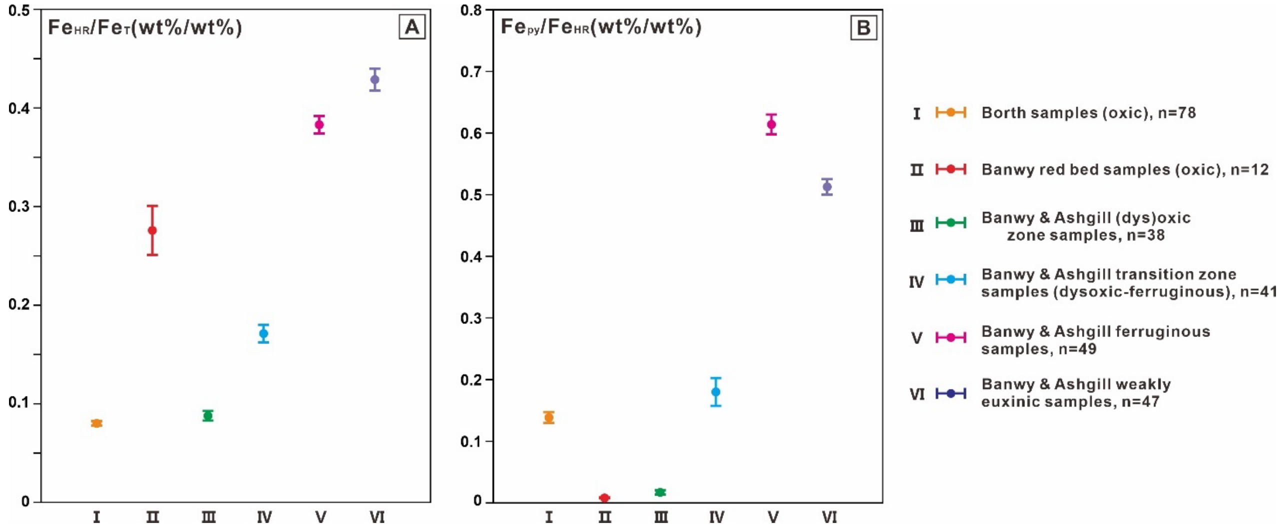


**Figure S5:** Summary of Fe speciation data for the Borth and Banwy River sections (Welsh Basin) and Ashgill Beck section, Lakesman Basin, UK. Oxic baseline samples are included for the Borth section. Circles represent the mean values, and whiskers represent 1σ.

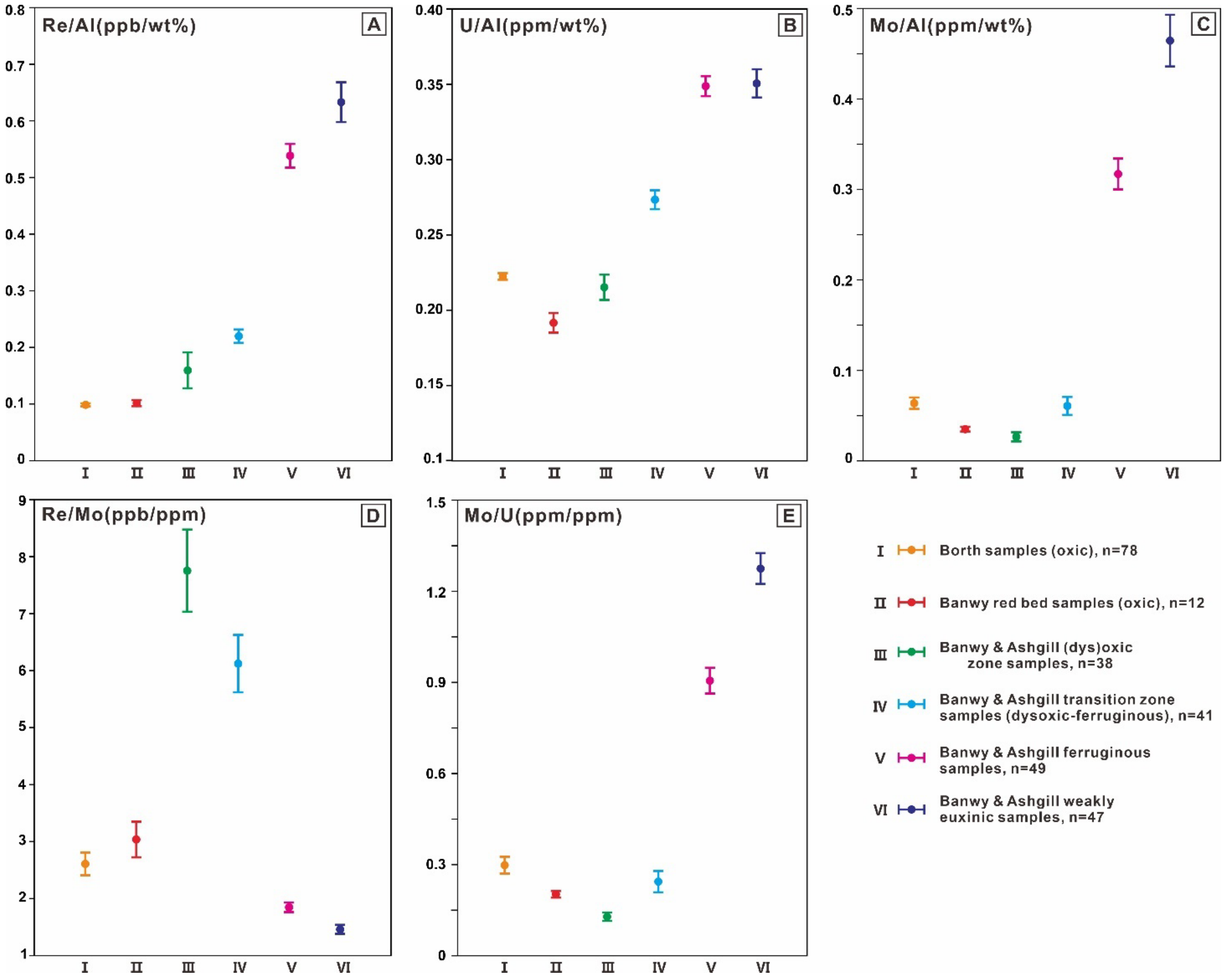


**Figure S6:** Summary of RSTM data for the Borth, Banwy River and Ashgill Beck sections. Circles represent the mean values and whiskers represent 1σ.

## Controls on U and Mo Isotope Compositions

*Evaluation of detrital influence*

To evaluate the potential detrital influence on U and Mo isotope compositions, we first cross-plot U and Mo concentrations against Al, a proxy for terrigenous input (Fig. S7). No significant correlation is observed for samples from Banwy River and Ashgill Beck, with anoxic samples commonly having higher U and Mo contents relative to (dys)oxic samples. This suggests a dominant redox control on U and Mo drawdown into the sediments. Similarly, (dys)oxic samples from both sections tend to exhibit lower U and Mo isotope values compared to samples with ferruginous or weakly euxinic conditions, although there is a degree of overlap in some $\delta^{238}U$ data (Fig. S7). Taken together, these patterns suggest that any detrital influence is negligible for most samples. However, the generally low background concentrations of redox-sensitive elements in our samples precludes the effective application of "excess values" to calculate authigenic U or Mo isotope compositions *(49)*, as this approach results in negative or near-zero "authigenic" values for samples characterized by low U or Mo enrichment. Consequently, we utilize bulk U and Mo isotope values to infer redox conditions.

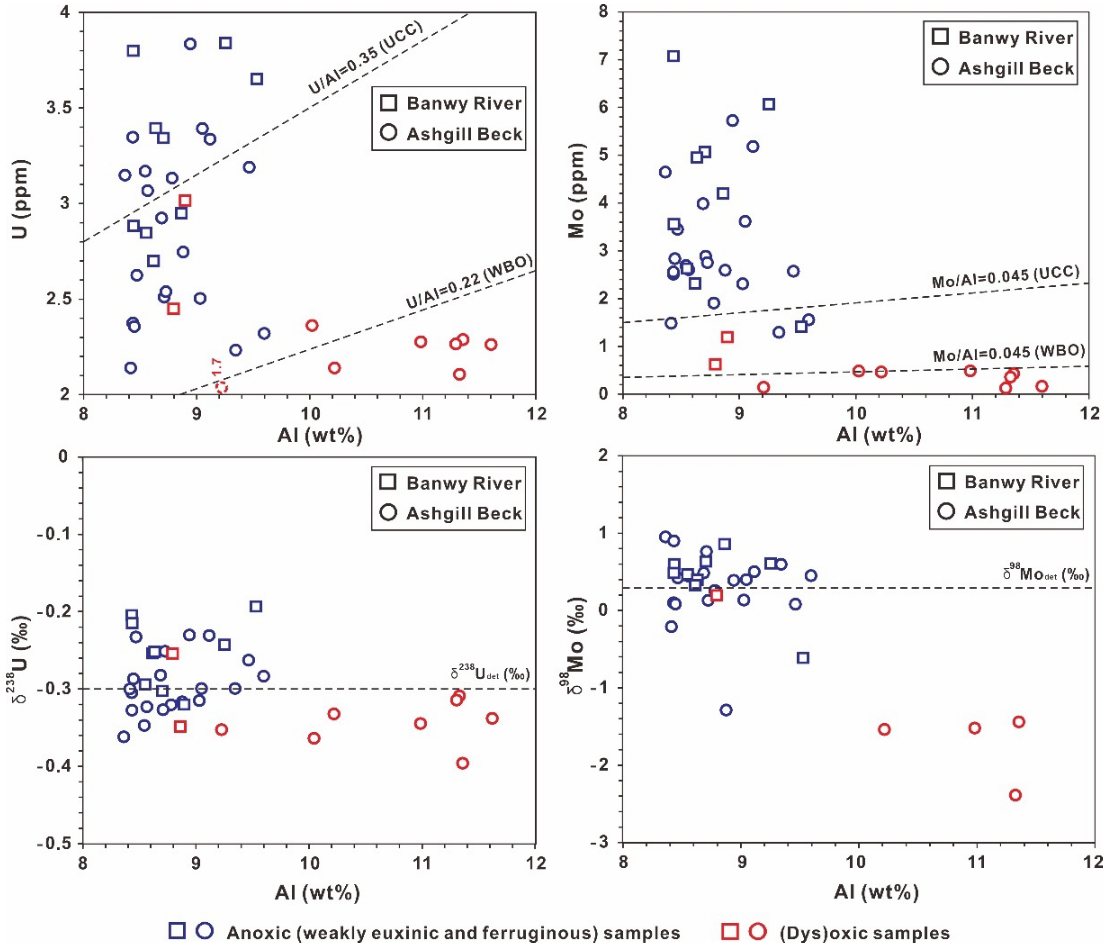


**Figure S7:** Bulk U and Mo concentrations and isotopic compositions plotted against Al content for samples from Banwy River and Ashgill Beck. The $\delta^{238}U_{det}$ and $\delta^{98}Mo_{det}$ values are estimated using average continental crust values of -0.3‰ and 0.3‰, respectively (*49, 104*).

*Local vs global $\delta^{238}U$-$\delta^{98}Mo$ isotope signatures*

Local environmental factors such as organic carbon loading and basin hydrography may influence U isotope fractionations *(48,105)*. In our samples, bulk $\delta^{238}U$ values show no correlation with TOC content (Fig. S8). This may be due to the relatively low TOC content (less than 1 wt%) even in anoxic samples, which could mask the effects of productivity and organic carbon availability on microbial U reduction and isotopic fractionation. Such variability aligns with observations by *(106)*, who reported highly variable $\delta^{238}U$ values in sediments deposited under modern and ancient ferruginous conditions.

Bulk $\delta^{238}U$ values generally covary positively with U/Al, with an increase in both parameters under oxic to anoxic conditions indicating higher authigenic U uptake from seawater under ferruginous or weakly euxinic conditions (Fig. S8). The observed increase in U concentrations and the accompanying shift in $\delta^{238}U$ values from oxic (average −0.33‰) to anoxic samples (average −0.28‰) primarily reflects changes in bottom-water redox conditions. Under increasingly reducing conditions, particularly those that are ferruginous and weakly euxinic, authigenic U uptake becomes more efficient, resulting in both higher U enrichments and more positive $\delta^{238}U$ signatures.

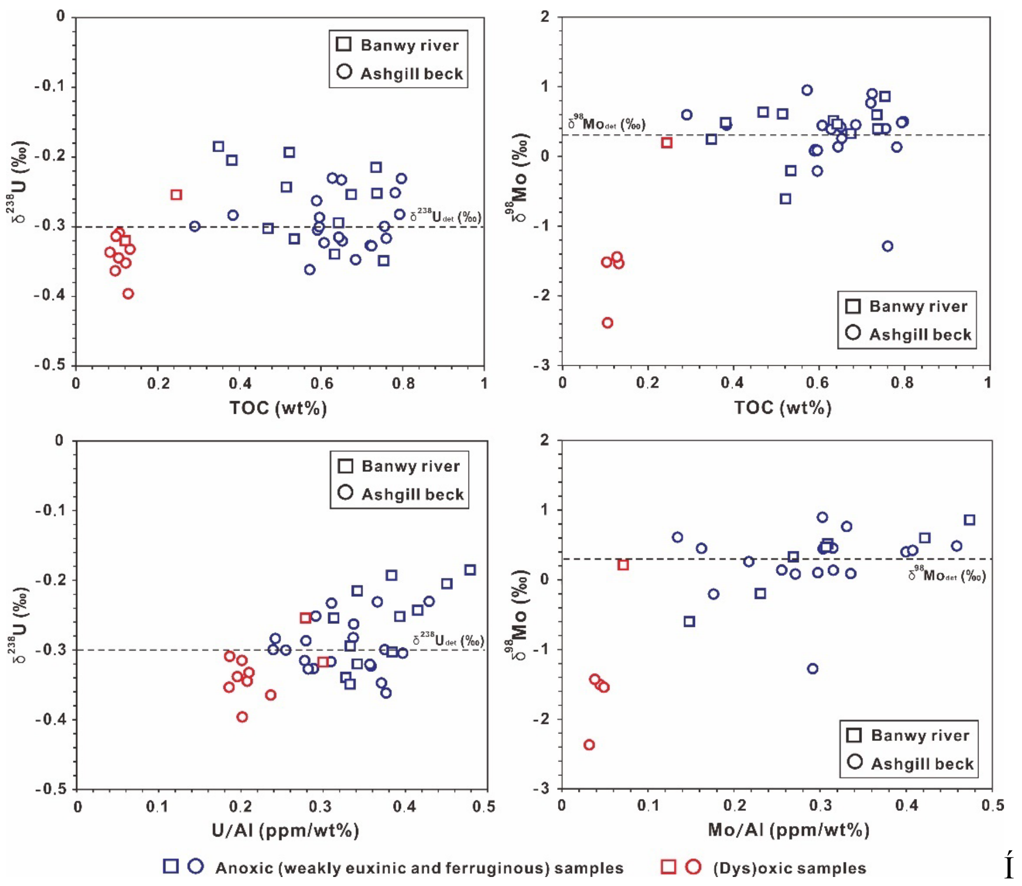


**Figure S8:** Cross-plots of $\delta^{238}$U and $\delta^{98}$Mo versus U and Mo concentrations and total organic carbon (TOC) for samples from the Banwy River and Ashgill Beck sections.

Bulk $\delta^{98}$Mo values show no systematic correlation with TOC or Mo/Al ratios (Fig. S7), suggesting that local organic carbon loading was not the primary control on $\delta^{98}$Mo fractionation *(107)*. In both the Banwy River and Ashgill Beck sections, anoxic samples have higher pyrite contents ($0.6 < Fe_{py}/Fe_{HR} < 0.8$) and elevated Mo/Al ratios (Fig. S3), indicating that enhanced sulfide availability likely drove Mo drawdown into the sediments *(108)*. The data suggest the prevalence of weakly euxinic conditions, which is supported by relatively low Mo/U ratios compared to highly euxinic modern basins (cf. *(27)*), as well as $Fe_{py}/Fe_{HR}$ ratios that generally fall towards the lower end of the 'possibly euxinic' zone (Fig. 2). As a result, Mo was variably and incompletely scavenged from deep waters, leading to highly variable $\delta^{98}$Mo values in ferruginous and weakly euxinic samples (Figs. S7 and S8). The Mo contents of samples for which we have $\delta^{98}$Mo data have an average enrichment of ~7.4 times, increasing from oxic (0.45 ± 0.31 ppm) to anoxic samples (3.32 ± 1.5 ppm) (Table S5). This increase in Mo values from oxic to anoxic samples indicates that, despite incomplete scavenging, a substantial authigenic Mo signal occurs in the anoxic samples. Furthermore, Mo concentrations in oxic red bed samples are likely derived from seawater through adsorption and trapping by Fe-Mn (oxyhydr)oxides, rather than from lithological sources.

Integrating redox constraints from multiple regional proxies (Fe speciation, and Re, U and Mo systematics), particularly when $\delta^{238}$U and $\delta^{98}$Mo are incorporated, provides a robust framework for reconstructing redox conditions from the regional to global scale (Fig. S9; e.g.,*(47,109,110)*). Collectively, the reductive removal of U and Mo, along with their associated isotopic fractionation, is closely tied to redox evolution and sulphide availability in the water column and/or pore waters. On a broader scale, these processes likely reflect global redox variability during the early to mid-Silurian Period.

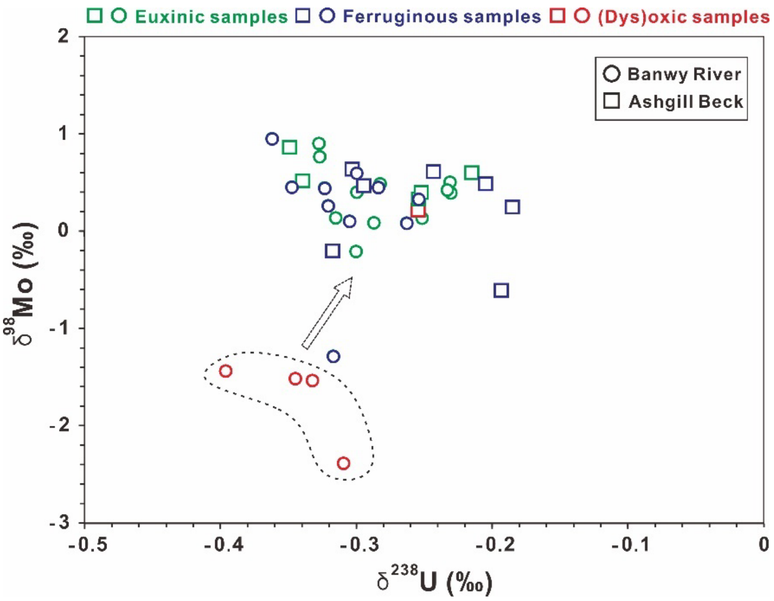


**Figure S9:** Bulk rock cross-plot of $\delta^{98}$Mo versus $\delta^{238}$U for sediments deposited under oxic, ferruginous and weakly euxinic conditions, as determined by Fe speciation and trace metal systematics, in the Banwy River and Ashgill Beck sections. Circled samples (including red bed samples) were collected from the Telychian *crispus* to *spiralis* biozones. The remaining samples were collected from the uppermost Telychian to lower Sheinwoodian (*insectus* to *centrifugus* biozones).

**Constraining marine redox conditions using the U-Mo isotope system**

*Reconstruction of seawater $\delta^{98}$Mo and $\delta^{238}$U*

To reconstruct global seawater redox conditions, we combine Mo and U isotope data from the studied rocks, focusing on their well-characterized redox-sensitive behaviour *(49,108)*. Under oxic conditions, Mo is predominantly removed via adsorption onto, or co-precipitation with, Fe–Mn (oxyhydr)oxides, yielding a strong isotopic fractionation (~3‰) between seawater (~ +2.3‰) and modern oxic sediments (~ -0.7‰) *(111-113)*. Based on this, we estimate that the seawater Mo isotope ($\delta^{98}Mo_{SW}$) value during the Telychian red bed interval (the most oxygenated phase in our record), was approximately +1.5‰. By contrast, anoxic samples deposited during the ESCIE interval yield similar $\delta^{98}$Mo values, consistent with ferruginous to weakly euxinic conditions ($[H_2S]_{aq}$ <11 μM) and incomplete Mo scavenging *(114-116)*. Given that sediments from modern anoxic continental margins typically record $\delta^{98}$Mo ≈ +0.7‰ *(117)*, we infer that $\delta^{98}Mo_{SW}$ declined to ~ +1.0‰ during the ESCIE, suggesting a broadening of anoxic seafloor conditions relative to the red bed interval.

Uranium isotopes provide complementary constraints. In oxic seawater, U behaves conservatively, while under reducing conditions it is removed from the water column, with associated isotopic fractionation dependent on the redox state of the depositional environment *(49,118-120)*. Because U is typically enriched in reduced sediments and preferentially incorporates heavier isotopes, authigenic $\delta^{238}$U values may serve as a maximum estimate for contemporaneous seawater values. For ferruginous and dysoxic samples in this study, we apply a +0.15‰ offset (cf. (*121)*), consistent with sediment $\delta^{238}$U values between –0.4‰ and –0.2‰ *(106, 122)*. By contrast, the three oxic red bed samples are assigned a –0.23‰ offset, reflecting adsorption of U(VI) onto Fe–Mn (oxyhydr)oxides, which consistently show lower $\delta^{238}$U relative to seawater *(123, 124)*.

Accordingly, we estimate a seawater $\delta^{238}U$ value of ~ –0.13‰ during the red bed interval and ~ –0.49‰ during the ESCIE, consistent with a transition to more widespread marine anoxia.

*Combined U-Mo isotope modelling*

Sedimentary Mo and U isotope systems are commonly used to evaluate global marine redox conditions in ancient environments through element mass balance and isotope fractionation models (e.g., *(37,38,46,125-127)*). However, both local environmental conditions and global ocean redox state may influence the Mo and U isotope compositions of sediments, complicating the interpretation of isotopic data, even in euxinic organic-rich mudstones with significant U and Mo enrichment *(64)*. Stockey *et al.(37)* utilized Monte Carlo simulations and cross-validated LOESS models to estimate potential redox conditions by interpreting U and Mo isotopes separately in euxinic black shales from the Rhuddanian Stage of the lowermost Silurian. By contrast, our study sections lack samples deposited under strong, long-term euxinic conditions, introducing greater uncertainty into the U-Mo isotope system. As discussed above, the $\delta^{98}Mo$ and $\delta^{238}U$ values in our dataset are derived from sediments deposited under oxic, through ferruginous, to weakly euxinic conditions, with low U and Mo concentrations (see Table S6). This results in a much wider range of possible seawater $\delta^{238}U$ and $\delta^{98}Mo$ values, due to variable degrees of fractionation during deposition *(106,117)*.

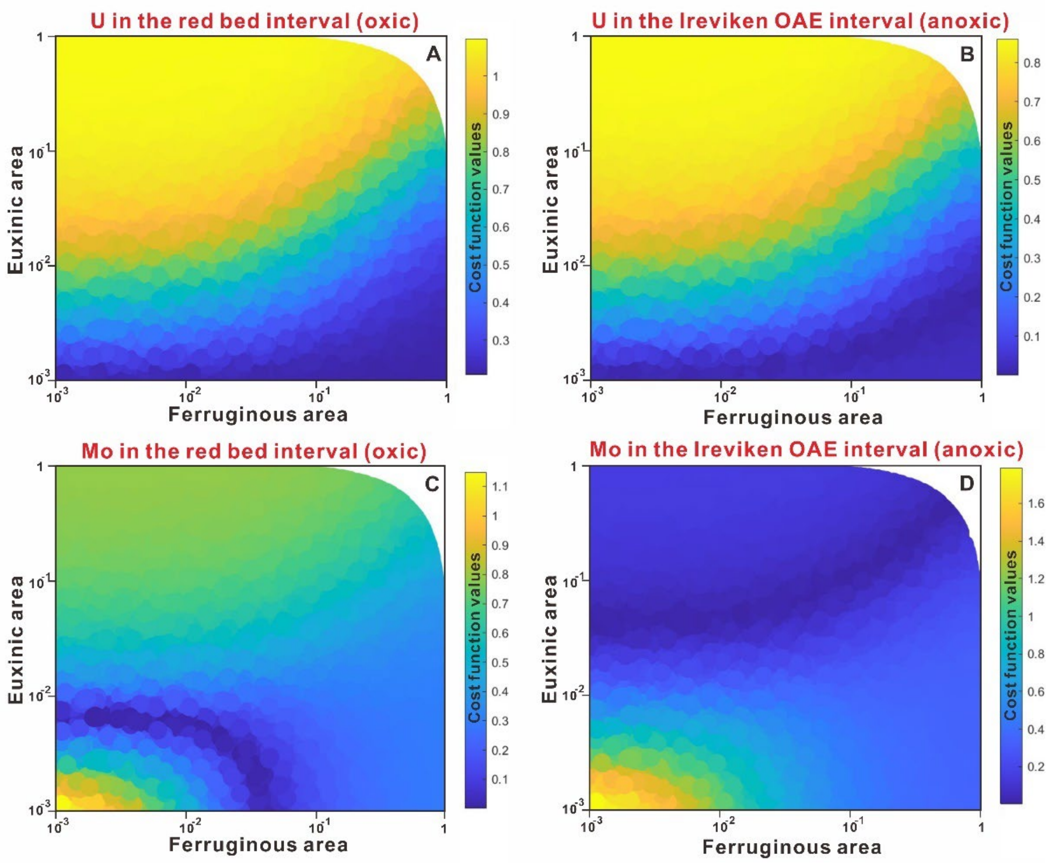


**Figure S10:** Schematic reconstruction of redox evolution using separate isotope mass balance model outputs for uranium (A and B) and molybdenum (C and D) during the mid Telychian red bed interval and the ESCIE interval. Colored dots represent the mathematical distance (cost function) between 100,000 model iterations and the average laboratory-derived U or Mo isotope fractionation values for the respective intervals. The dark blue shaded regions indicate the lowest cost function values, highlighting the most likely marine redox scenarios.

To address these issues, we ran a large ensemble of isotope mass balance models to determine the marine redox landscape that is most consistent with the combined isotope measurements *(47)*. We used a cost function approach to determine the most likely areal extent of ferruginous and euxinic seafloor. Figure S10 shows the cost function for each isotope measurement considered in isolation, which indicates how close the model isotopic value is to the data for the full range of euxinic and ferruginous seafloor areas tested in the model. For $\delta^{238}U$ (Fig. S10 A, B), ferruginous areas from ~0–100% of the seafloor are all reasonably compatible (i.e., they have a low cost function value), with the data for both oxic mid-Telychian and anoxic ESCIE samples providing limited interpretative value. However, the $\delta^{238}U$ values in our samples certainly do not support widespread marine euxinia (<1%; Fig. S10 A, B), which has a much higher cost function. By contrast, $\delta^{98}Mo$ values show a more pronounced shift between the sampled intervals (Fig. S10 C, D). The average $\delta^{98}Mo$ values change significantly from the mid-Telychian red bed samples (-1.50 ± 0.05‰) to the anoxic IBE samples (0.32‰), reflecting distinct marine redox conditions. In the main text we integrate the $\delta^{98}Mo$ and $\delta^{238}U$ comparisons together within a single cost function framework, in order to determine a more robust redox interpretation for both the Telychian red bed depositional interval and the following ESCIE interval (main text; Fig. 3).

**Paleoclimatic controls**

*Sheinwoodian Oxygen Isotope Excursion (SOIE)*

Conodont apatite oxygen isotope ($\delta^{18}O_{phos}$) data from stratigraphic sections in Laurentia (Cornwallis and Anticosti Islands) and Baltica (Estonia and Latvia) reveal a long-term warming trend following the Hirnantian glaciation at the end of the Ordovician. This warming persisted

through the Rhuddanian and Aeronian stages until the early-mid Telychian (~437 Ma, *crispus* Biozone). Subsequently, $\delta^{18}O_{phos}$ values indicate a gradual cooling trend beginning in the latest Telychian (*lapworthi* Biozone), culminating in a major early Wenlock cooling peak *(5, 16)*. In detail, the spread of $\delta^{18}O_{phos}$ values in the Llandovery-Wenlock boundary interval suggests an oscillating cooling trend, with an overall increase of 2‰, corresponding to a potential temperature drop of ~9°C (ref. *(16)*). The cooling coincides with evidence for glaciation, including tillites found in the Amazon and Paraná basins of Brazil, as well as the Andean basins of Argentina, Bolivia and Peru, which indicate two phases, the first in the late Telychian and the second in the early Sheinwoodian *(128-130)* (Fig. 4).

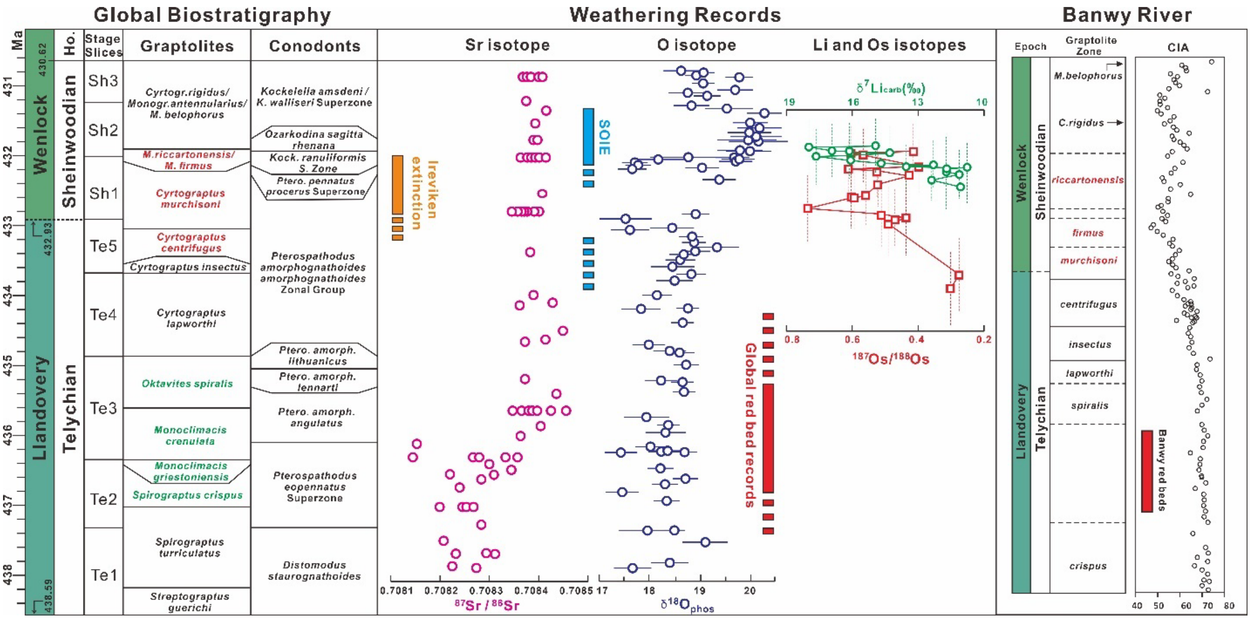


**Figure S11:** Long term Sr, O, Li and Os isotope records, in the early Silurian *(30)* and the Chemical Index of Alteration (CIA) profile at Banwy River. Data from calcitic brachiopod $^{87}Sr/^{86}Sr$ ratios are from *(131,132)*. Oxygen isotope records ($\delta^{18}O_{phos}$) of conodont apatite are from *(16)*. Bulk carbonate lithium isotope ($\delta^{7}Li_{carb}$) and shale osmium isotope ($^{187}Os/^{188}Os$) data are from *(133)*

*Weathering proxies*

A decline in chemical weathering, beginning in the late Telychian, is seen in the chemical index of alteration (CIA) at Banwy River (Fig. S11). The CIA values were calculated as CIA = $Al_2O_3/(Al_2O_3+CaO^*+Na_2O+K_2O) \times 100$, with CaO* representing CaO incorporated into silicate minerals *(134)*. To isolate this component, contributions from carbonate and phosphate minerals were removed following the method of *(135).* All $P_2O_5$ was assumed to occur as apatite, and the corresponding CaO was subtracted stoichiometrically. As Ca is more mobile than Na during chemical weathering *(136)*, CaO was taken to equal the remaining CaO if it was less than $Na_2O$; otherwise, CaO was assumed equivalent to $Na_2O$. A subtle decline in CIA values occurs as early as the upper part of the *spiralis* Biozone (Fig. S11), potentially documenting a decline in weathering intensity driven by the onset of global cooling *(134,135,137)* considerably before the global expansion of anoxia in the latest Telychian, when CIA values reach a minimum (Fig. 2). This is followed by a degree of variability, with higher CIA values occurring in the late *riccartonensis* Biozone as the water column in the River Banwy section transitioned from weakly euxinic to ferruginous, implying a return to more intense chemical weathering as global climatic conditions recovered during the terminal stage of the ESCIE (Fig. 2).

Calcitic brachiopod shell Sr isotope records from Anticosti Island (Canada), Wales, Gotland (Sweden), Podolia (Ukraine), Latvia and Lithuania reveal progressive increases in $^{87}Sr/^{86}Sr$ values from 0.707930 to 0.708792 through the Silurian, reflecting a possible increased riverine flux of radiogenic Sr to the oceans, driven by enhanced weathering of continental sialic rocks under a progressive, long-term warming climate *(131)*. In the mid-Telychian, there is an exceptionally rapid increase in $^{87}Sr/^{86}Sr$ values (~0.0003), which may represent a strong silicate weathering peak under

warm climatic conditions (Fig. S11). Subsequently, the upward trajectory slows and briefly stabilizes from the mid Telychian to the mid Sheinwoodian, with a slight decrease observed in the lower *murchisoni* Biozone (Fig. S11). While the Silurian long-term $^{87}Sr/^{86}Sr$ trend continues to rise following the end-Ordovician Hirnantian glaciation, this short-term deviation from the broader increase may indicate a temporary decline in silicate weathering rates from the late Telychian into the early Sheinwoodian, potentially linked to early Sheinwoodian cooling and glacial conditions, but given high global sea-level at this time *(22)*, this is unlikely.

The carbonate $\delta^7Li$ record from the Lusklint and Lickershamn sections of Gotland exhibits a significant increase in the lower Sheinwoodian, rising from a minimum of 11‰ to a peak of 17.6‰ (Fig. S4; data from *(133)*). Similarly, $^{187}Os/^{188}Os$ isotope ratios in shales and carbonates from the Llandovery to Wenlock interval of the Aizpute-41 core display a general increasing trend, spanning the upper Telychian to lower Sheinwoodian (Fig. S4; data from *(133)*).

The combined evidence of long-term $^{87}Sr/^{86}Sr$ trends through the Telychian and Sheinwoodian stages, with elevated $^{187}Os/^{188}Os$ ratios, and increased $\delta^7Li$ and $\delta^{18}O$ values, indicates a change in weathering from a mid-Telychian greenhouse climate to glacial conditions in the early Sheinwoodian. Elevated $^{187}Os/^{188}Os$ ratios and increased $\delta^7Li$ values imply enhanced physical erosion coupled with subdued but isotopically distinct chemical weathering. Ice sheet activity may have exposed unweathered bedrock, including radiogenic Os-rich lithologies, such as black shales, which contributed high $^{187}Os$ fluxes to the ocean. Simultaneously, freshly-eroded mineral surfaces supported limited chemical alteration under glacial conditions, where fractionation of lithium isotopes occurred as light $^6Li$ was sequestered into secondary clays, leaving heavier $^7Li$ in solution. Enrichment in $\delta^{18}O$ values in phosphate ($\delta^{18}O_{phos}$) during the mid-Sheinwoodian (Fig. S11),

corroborate the suppression of chemical weathering during cooling *(16)*.

*Impact of Ireviken deoxygenation*

A comparison of the icehouse-triggered Ireviken deoxygenation event with greenhouse-triggered carbon perturbation events of the Mesozoic demonstrates clear differences (Fig. S12). Unlike Mesozoic OAEs *(55,139)*, the Ireviken deoxygenation appears to have developed in the absence of major volcanism, and instead coincided with global cooling, glacio-eustatic sea-level fluctuations, and the upwelling of ferruginous deep waters under a low-oxygen atmosphere. The well-oxygenated background state of Mesozoic oceans contrasts with that of mid-Paleozoic oceans, which were dysoxic to ferruginous at depth (this study and cf. *(140, 141))*. The longer duration and muted trace metal isotope signatures of Silurian OAEs *(15)* reflect a redox feedback system in which phosphorus recycling was likely suppressed by iron mineral scavenging under ferruginous conditions (e.g., *(54)*), thereby weakening the productivity–anoxia loop that characterized many Mesozoic events (e.g., *(15,142,143)*).

Paleozoic oceanic anoxic events were frequently accompanied by biotic crises, which is a relationship also seen during younger OAEs. The extent of deoxygenation during the Ireviken Event may have approached that of the Permian–Triassic boundary (Fig. S12). However, the consequences for life were not as severe, an observation that may reflect a somewhat greater resilience of mid-Paleozoic marine life to low oxygen levels when compared to Mesozoic biota.

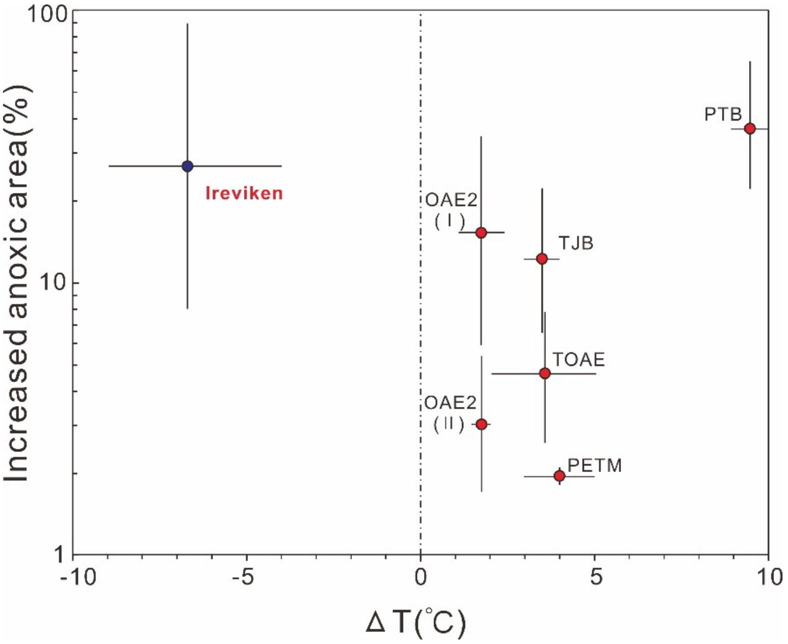


**Figure S12:** Plot showing the mean increase in marine anoxic area versus temperature change, comparing the cooling-driven Ireviken OAE with warming-driven Mesozoic OAEs. Data are from *(55,138)*. Black lines represent the full range of values used to calculate the mean and are not indicative of error ranges.

## Supplementary Tables

***Table S1. Organic C and pyrite S isotopes, and Fe speciation contents, in Banwy River samples, east Wales, UK.***

| Name | Height (m) | Lithofacies Description | $\delta^{13}C_{org}$ (‰) | TOC (wt%) | $\delta^{34}S_{py}$ (‰) | $Fe_T$ (wt%) | $Fe_{carb}$ (wt%) | $Fe_{ox}$ (wt%) | $Fe_{mag}$ (wt%) | $Fe_{py}$ (wt%) | $Fe_{HR}/Fe_T$ | $Fe_{py}/Fe_{HR}$ |
|---|---|---|---|---|---|---|---|---|---|---|---|---|
| BR148 | 188.59 | finely laminated, medium grey mudstone | -27.86 | 0.61 | -17.03 | 4.59 | 0.82 | 0.12 | 0.14 | 0.98 | 0.45 | 0.48 |
| BR147 | 187.39 | finely laminated, medium grey mudstone | -28.45 | 0.63 | -16.69 | 4.71 | 0.82 | 0.11 | 0.13 | 1.25 | 0.49 | 0.54 |
| BR146 | 186.39 | finely laminated, medium grey mudstone | -27.19 | 0.50 | -13.82 | 4.42 | 0.78 | 0.11 | 0.15 | 0.85 | 0.43 | 0.45 |
| BR145 | 185.39 | finely laminated, medium grey mudstone | -27.19 | 0.69 | -14.00 | 4.31 | 0.84 | 0.11 | 0.14 | 0.98 | 0.48 | 0.47 |
| BR144 | 184.29 | finely laminated, medium grey mudstone | -26.91 | 0.63 | -13.93 | 4.04 | 0.77 | 0.10 | 0.13 | 0.99 | 0.49 | 0.50 |
| BR143 | 183.29 | finely laminated, medium grey mudstone | -27.52 | 0.51 | -14.77 | 4.35 | 0.67 | 0.09 | 0.13 | 0.93 | 0.42 | 0.51 |
| BR142 | 182.19 | finely laminated, medium grey mudstone | -27.36 | 0.67 | -15.31 | 4.57 | 0.78 | 0.11 | 0.15 | 1.12 | 0.47 | 0.52 |
| BR141 | 181.19 | finely laminated, medium grey mudstone | -28.00 | 0.59 | -14.72 | 4.50 | 0.76 | 0.11 | 0.15 | 0.91 | 0.43 | 0.47 |
| BR140 | 180.09 | finely laminated, medium grey mudstone | -26.97 | 0.66 | -12.94 | 4.40 | 0.81 | 0.11 | 0.15 | 0.91 | 0.45 | 0.46 |
| BR139 | 178.99 | finely laminated, medium grey mudstone | -27.01 | 0.67 | -12.21 | 4.53 | 0.94 | 0.14 | 0.15 | 1.07 | 0.51 | 0.46 |
| BR138 | 177.89 | finely laminated, medium grey mudstone | -27.86 | 0.61 | -10.63 | 4.24 | 0.76 | 0.11 | 0.13 | 1.01 | 0.47 | 0.51 |
| BR137 | 176.69 | finely laminated, medium grey mudstone | -27.22 | 0.76 | -13.14 | 4.37 | 0.88 | 0.10 | 0.13 | 1.05 | 0.49 | 0.49 |
| BR136 | 175.59 | finely laminated, medium grey mudstone | -28.00 | 0.71 | -13.23 | 4.49 | 0.93 | 0.11 | 0.13 | 1.16 | 0.52 | 0.50 |
| BR135 | 174.49 | finely laminated, medium grey mudstone | -29.22 | 0.61 | -13.06 | 4.50 | 0.88 | 0.10 | 0.14 | 1.21 | 0.52 | 0.52 |
| BR134 | 173.39 | finely laminated, medium grey mudstone | -29.08 | 0.77 | -14.71 | 4.63 | 0.77 | 0.11 | 0.12 | 1.26 | 0.49 | 0.56 |
| BR133 | 172.19 | finely laminated, medium grey mudstone | -27.79 | 0.61 | -12.46 | 4.40 | 0.84 | 0.10 | 0.14 | 1.11 | 0.50 | 0.51 |
| BR132 | 171.09 | finely laminated, medium grey mudstone | -27.34 | 0.57 | -13.65 | 4.56 | 0.91 | 0.11 | 0.13 | 1.37 | 0.55 | 0.54 |
| BR131 | 169.99 | finely laminated, medium grey mudstone | -26.74 | 0.56 | -13.92 | 4.68 | 0.76 | 0.10 | 0.13 | 1.81 | 0.60 | 0.65 |
| BR130 | 168.99 | finely laminated, medium grey mudstone | -26.67 | 0.64 | -13.25 | 4.56 | 0.86 | 0.11 | 0.14 | 0.57 | 0.37 | 0.34 |

| | | | | | | | | | | | | |
|---|---|---|---|---|---|---|---|---|---|---|---|---|
| BR129 | 167.49 | finely laminated, medium grey mudstone | -27.23 | 0.58 | -14.71 | 4.41 | 0.90 | 0.11 | 0.14 | 1.12 | 0.51 | 0.49 |
| BR128 | 166.39 | finely laminated, dark grey mudstone | -28.31 | 0.59 | -11.53 | 4.07 | 0.66 | 0.09 | 0.11 | 0.90 | 0.44 | 0.51 |
| BR127 | 165.29 | finely laminated, dark grey mudstone | -27.11 | 0.69 | -13.51 | 4.71 | 0.81 | 0.11 | 0.14 | 1.39 | 0.52 | 0.57 |
| BR126 | 164.19 | finely laminated, dark grey mudstone | -27.03 | 0.57 | -12.38 | 4.59 | 0.90 | 0.14 | 0.17 | 0.94 | 0.47 | 0.44 |
| BR125 | 163.09 | finely laminated, dark grey mudstone | -27.34 | 0.60 | -10.72 | 4.58 | 1.03 | 0.10 | 0.12 | 1.43 | 0.59 | 0.53 |
| BR124 | 161.99 | finely laminated, dark grey mudstone | -27.10 | 0.65 | -12.22 | 4.22 | 0.74 | 0.09 | 0.11 | 0.94 | 0.45 | 0.50 |
| BR123 | 160.89 | finely laminated, dark grey mudstone | -28.84 | 0.78 | -14.11 | 4.55 | 0.85 | 0.11 | 0.14 | 0.97 | 0.46 | 0.47 |
| BR122 | 159.89 | finely laminated, dark grey mudstone | -27.34 | 0.80 | -15.10 | 4.60 | 0.87 | 0.11 | 0.14 | 1.32 | 0.53 | 0.54 |
| BR121 | 158.79 | dark grey mudsotne, some pyritic laminae | -28.12 | 0.80 | -11.98 | 4.62 | 0.70 | 0.09 | 0.12 | 1.10 | 0.43 | 0.55 |
| BR120 | 157.49 | dark grey mudstone, laminae indistinct | -27.98 | 0.64 | -8.42 | 4.38 | 0.92 | 0.12 | 0.15 | 0.84 | 0.46 | 0.41 |
| BR110 | 156.49 | finely laminated, dark grey mudstone | -27.93 | 0.72 | / | 4.57 | 0.62 | 0.10 | 0.09 | 1.29 | 0.46 | 0.61 |
| BR109 | 154.89 | finely laminated, dark grey mudstone | -28.84 | 0.78 | -14.12 | 4.60 | 0.60 | 0.08 | 0.09 | 1.29 | 0.45 | 0.62 |
| BR108 | 152.69 | finely laminated, dark grey mudstone | -27.68 | 0.78 | -13.16 | 4.64 | 0.62 | 0.08 | 0.10 | 1.21 | 0.43 | 0.60 |
| BR107 | 150.49 | finely laminated, dark grey mudstone | -29.03 | 0.79 | -12.22 | 4.72 | 0.59 | 0.09 | 0.10 | 1.31 | 0.44 | 0.63 |
| BR106 | 148.84 | finely laminated, dark grey mudstone | -28.65 | 0.60 | -13.48 | 4.55 | 0.06 | 0.60 | 0.06 | 1.11 | 0.40 | 0.60 |
| BR105 | 147.14 | finely laminated, dark grey mudstone | -27.15 | 0.67 | -12.82 | 4.42 | 0.57 | 0.07 | 0.08 | 1.30 | 0.46 | 0.64 |
| BR104 | 145.84 | finely laminated, dark grey mudstone | -28.51 | 0.76 | -12.93 | 4.78 | 0.52 | 0.04 | 0.07 | 1.32 | 0.41 | 0.68 |
| BR103 | 144.74 | finely laminated, dark grey mudstone | -27.46 | 0.64 | -12.65 | 4.54 | 0.47 | 0.04 | 0.07 | 2.15 | 0.60 | 0.79 |
| BR102 | 143.09 | finely laminated, dark grey mudstone | -27.06 | 0.75 | -13.40 | 4.75 | 0.42 | 0.16 | 0.07 | 1.26 | 0.40 | 0.66 |
| BR101 | 141.29 | finely laminated, dark grey mudstone | -28.52 | 0.78 | -14.25 | 4.62 | 0.55 | 0.05 | 0.07 | 1.21 | 0.41 | 0.64 |
| BR100 | 139.79 | finely laminated, dark grey mudstone | -27.67 | 0.72 | -12.65 | 4.64 | 0.57 | 0.07 | 0.07 | 0.95 | 0.36 | 0.57 |
| BR99 | 138.59 | finely laminated, dark grey mudstone | -27.14 | 0.88 | -12.82 | 4.87 | 0.62 | 0.05 | 0.07 | 0.59 | 0.27 | 0.44 |
| BR98 | 137.29 | finely laminated, dark grey mudstone | -27.35 | 0.72 | -16.19 | 4.51 | 0.65 | 0.04 | 0.07 | 1.27 | 0.45 | 0.62 |
| BR97 | 136.69 | finely laminated, dark grey mudstone | -26.93 | 0.67 | / | 4.65 | 0.60 | 0.04 | 0.07 | 1.34 | 0.44 | 0.66 |
| BR96 | 135.19 | finely laminated, medium grey mudstone | -28.85 | 0.68 | -16.59 | 4.48 | 0.56 | 0.08 | 0.07 | 0.53 | 0.28 | 0.43 |
| BR95 | 133.69 | finely laminated, medium grey mudstone | -29.10 | 0.65 | -16.80 | 4.61 | 0.62 | 0.04 | 0.06 | 1.37 | 0.45 | 0.65 |

| | | | | | | | | | | | | |
|---|---|---|---|---|---|---|---|---|---|---|---|---|
| BR94 | 132.59 | finely laminated, medium grey mudstone | -27.05 | 0.70 | -17.04 | 4.45 | 0.54 | 0.05 | 0.07 | 1.11 | 0.40 | 0.63 |
| BR93 | 131.49 | finely laminated, medium grey mudstone | -26.69 | 0.61 | -16.23 | 4.41 | 0.59 | 0.05 | 0.07 | 1.24 | 0.44 | 0.63 |
| BR92 | 130.39 | finely laminated, medium grey mudstone | -27.05 | 0.60 | -15.86 | 4.49 | 0.65 | 0.07 | 0.07 | 0.76 | 0.35 | 0.49 |
| BR91 | 129.29 | finely laminated, medium grey mudstone | -27.09 | 0.56 | -17.30 | 4.64 | 0.63 | 0.07 | 0.07 | 1.19 | 0.42 | 0.61 |
| BR90 | 127.98 | finely laminated, medium grey mudstone | -27.19 | 0.60 | -18.65 | 4.67 | 0.64 | 0.04 | 0.08 | 1.18 | 0.42 | 0.61 |
| BR89 | 126.68 | finely laminated, medium grey mudstone | -28.35 | 0.36 | -15.73 | 4.65 | 0.46 | 0.04 | 0.08 | 0.98 | 0.34 | 0.63 |
| BR88 | 125.18 | medium grey mudstone | -27.60 | 0.24 | / | 5.17 | 0.44 | 0.05 | 0.15 | 0.02 | 0.13 | 0.02 |
| BR87 | 123.68 | medium grey mudstone | -28.17 | 0.17 | / | 5.62 | 0.47 | 0.05 | 0.16 | 0.01 | 0.12 | 0.01 |
| BR86 | 122.78 | medium grey mudstone | -27.77 | 0.17 | -15.80 | 5.89 | 0.37 | 0.05 | 0.15 | 0.04 | 0.10 | 0.07 |
| BR85 | 121.18 | medium grey mudstone | -27.52 | 0.16 | -23.63 | 4.02 | 0.33 | 0.03 | 0.15 | 0.04 | 0.14 | 0.07 |
| BR84 | 119.68 | finely laminated, medium grey mudstone | -28.55 | 0.50 | -19.80 | 4.40 | 0.49 | 0.05 | 0.09 | 0.74 | 0.31 | 0.54 |
| BR83 | 118.18 | finely laminated, medium grey mudstone, small burrows | -27.63 | 0.28 | -20.98 | 3.88 | 0.45 | 0.03 | 0.08 | 0.25 | 0.21 | 0.31 |
| BR82 | 117.23 | finely laminated, medium grey mudstone | -28.96 | 0.37 | / | 4.45 | 0.47 | 0.04 | 0.10 | 0.64 | 0.28 | 0.51 |
| BR81 | 116.13 | finely laminated, medium grey mudstone | -27.22 | 0.42 | -17.57 | 4.69 | 0.44 | 0.04 | 0.09 | 0.49 | 0.23 | 0.46 |
| BR80 | 115.03 | finely laminated, medium grey mudstone | -27.28 | 0.42 | -16.77 | 4.52 | 0.51 | 0.08 | 0.10 | 0.58 | 0.28 | 0.45 |
| BR79 | 113.93 | finely laminated, medium grey mudstone | -28.56 | 0.32 | -18.26 | 4.54 | 0.48 | 0.04 | 0.11 | 0.19 | 0.18 | 0.23 |
| BR78 | 112.83 | finely laminated, medium grey mudstone | -28.04 | 0.35 | -21.46 | 4.55 | 0.44 | 0.04 | 0.12 | 0.11 | 0.16 | 0.16 |
| BR77 | 111.93 | finely laminated, medium grey mudstone | -28.38 | 0.36 | -17.54 | 4.84 | 0.40 | 0.04 | 0.13 | 0.38 | 0.20 | 0.41 |
| BR76 | 111.13 | medium grey mudstone with burrows | -28.83 | 0.22 | -13.54 | 4.92 | 0.38 | 0.05 | 0.14 | 0.06 | 0.13 | 0.10 |
| BR75 | 110.13 | finely laminated, medium grey mudstone | -28.64 | 0.26 | -19.24 | 5.02 | 0.43 | 0.04 | 0.14 | 0.14 | 0.15 | 0.19 |
| BR74 | 109.43 | finely laminated, medium grey mudstone | -27.43 | 0.33 | / | 4.96 | 0.37 | 0.04 | 0.14 | 0.19 | 0.15 | 0.26 |
| BR73 | 108.53 | light grey mudstone with concretions | -27.47 | 0.22 | -20.33 | 4.98 | 0.45 | 0.04 | 0.14 | 0.01 | 0.13 | 0.02 |
| BR72 | 107.93 | finely laminated, medium grey mudstone | -28.73 | 0.33 | -17.68 | 4.81 | 0.53 | 0.06 | 0.12 | 0.29 | 0.21 | 0.29 |
| BR71 | 106.93 | finely laminated, medium grey mudstone | -28.36 | 0.29 | -14.56 | 4.51 | 0.51 | 0.04 | 0.12 | 0.17 | 0.19 | 0.20 |
| BR69 | 105.03 | light grey mudstone | -28.40 | 0.12 | -24.56 | 2.45 | 0.79 | 0.02 | 0.08 | 0.03 | 0.38 | 0.03 |

| | | | | | | | | | | | | | |
|---|---|---|---|---|---|---|---|---|---|---|---|---|---|
| BR68 | 104.18 | finely laminated, medium grey mudstone | -27.60 | 0.34 | -25.18 | 4.74 | 0.50 | 0.04 | 0.13 | 0.16 | 0.17 | 0.19 |
| BR67 | 103.68 | light grey mudstone | -27.72 | 0.18 | / | 5.11 | 0.43 | 0.08 | 0.20 | 0.03 | 0.14 | 0.04 |
| BR66 | 102.88 | finely laminated, medium grey mudstone | -27.76 | 0.32 | -17.82 | 5.01 | 0.48 | 0.07 | 0.15 | 0.08 | 0.16 | 0.11 |
| BR65 | 102.18 | light grey mudstone | -27.51 | 0.19 | / | 4.94 | 0.54 | 0.04 | 0.15 | 0.21 | 0.19 | 0.23 |
| BR64 | 101.98 | finely laminated, medium grey mudstone | -27.58 | 0.37 | -17.10 | 4.98 | 0.43 | 0.04 | 0.13 | 0.34 | 0.19 | 0.36 |
| BR63 | 101.38 | light grey mudstone | / | 0.27 | -23.35 | 5.23 | 0.43 | 0.05 | 0.17 | 0.11 | 0.14 | 0.15 |
| BR62 | 100.83 | finely laminated, medium grey mudstone | -27.58 | 0.41 | -13.42 | 5.07 | 0.43 | 0.04 | 0.16 | 0.26 | 0.18 | 0.29 |
| BR61 | 100.13 | light grey mudstone | -28.53 | 0.21 | / | 5.66 | 0.35 | 0.06 | 0.19 | 0.02 | 0.11 | 0.03 |
| BR60 | 99.43 | light grey mudstone | -27.59 | 0.22 | -22.12 | 5.22 | 0.57 | 0.06 | 0.17 | 0.04 | 0.16 | 0.04 |
| BR59 | 98.78 | light grey mudstone | -27.56 | 0.11 | -27.14 | 5.40 | 0.50 | 0.04 | 0.17 | 0.05 | 0.14 | 0.06 |
| BR58 | 98.23 | medium grey, laminated mudstone with small burrows | -27.35 | 0.21 | -17.64 | 5.23 | 0.37 | 0.04 | 0.15 | 0.19 | 0.14 | 0.25 |
| BR57 | 97.63 | medium grey, laminated mudstone with small burrows | -28.02 | 0.30 | -27.46 | 4.91 | 0.41 | 0.04 | 0.15 | 0.04 | 0.13 | 0.06 |
| BR56 | 97.18 | medium grey, laminated mudstone | -27.43 | 0.29 | -25.14 | 5.33 | 0.33 | 0.04 | 0.16 | 0.12 | 0.12 | 0.18 |
| BR55 | 97.13 | medium grey mudstone | -27.63 | 0.15 | -24.11 | 5.92 | 0.73 | 0.05 | 0.20 | 0.11 | 0.18 | 0.11 |
| BR54 | 96.23 | dark, laminated mudstone | -27.93 | 0.31 | -23.55 | 5.30 | 0.40 | 0.05 | 0.17 | 0.07 | 0.13 | 0.10 |
| BR53 | 95.83 | dark, laminated mudstone | -27.19 | 0.38 | -23.16 | 5.13 | 0.48 | 0.04 | 0.17 | 0.21 | 0.17 | 0.24 |
| BR52 | 95.43 | dark, laminated mudstone | -28.25 | 0.32 | -24.85 | 5.27 | 0.46 | 0.05 | 0.16 | 0.01 | 0.13 | 0.02 |
| BR51 | 93.63 | medium grey, laminated mudstone with small burrows | -27.20 | 0.29 | / | 5.48 | 0.52 | 0.05 | 0.19 | 0.21 | 0.18 | 0.22 |
| BR50 | 91.63 | medium grey, laminated mudstone with small burrows | -27.38 | 0.15 | / | 5.59 | 0.52 | 0.04 | 0.21 | 0.08 | 0.15 | 0.09 |
| BR49 | 90.43 | medium grey mudstone | -27.38 | 0.12 | / | 6.10 | 0.51 | 0.04 | 0.21 | 0.05 | 0.13 | 0.07 |
| BR48 | 88.43 | medium grey mudstone | -27.25 | 0.18 | -29.10 | 5.17 | 0.46 | 0.05 | 0.18 | 0.04 | 0.14 | 0.06 |
| BR47 | 86.43 | dark grey mudstone | -28.47 | 0.37 | -31.24 | 4.54 | 0.40 | 0.06 | 0.15 | 0.11 | 0.16 | 0.15 |

| | | | | | | | | | | | | | |
|---|---|---|---|---|---|---|---|---|---|---|---|---|---|
| BR46 | 84.43 | medium grey mudstone | -27.43 | 0.13 | / | 6.08 | 0.21 | 0.03 | 0.14 | 0.00 | 0.06 | 0.01 |
| BR45 | 82.43 | laminated medium grey mudstone | -28.05 | 0.17 | / | 4.46 | 0.36 | 0.03 | 0.13 | 0.01 | 0.12 | 0.02 |
| BR44 | 80.37 | finely laminated black shale | -27.27 | 0.21 | / | 4.79 | 0.24 | 0.03 | 0.17 | 0.02 | 0.09 | 0.03 |
| BR43 | 78.72 | light grey mudstone | -27.68 | 0.10 | / | 5.02 | 0.28 | 0.03 | 0.17 | 0.06 | 0.11 | 0.11 |
| BR44 | 76.72 | light grey mudstone | -27.01 | 0.12 | / | 5.78 | 0.23 | 0.03 | 0.15 | 0.01 | 0.07 | 0.02 |
| BR41 | 74.62 | light grey mudstone | -27.01 | 0.10 | / | 6.10 | 0.21 | 0.08 | 0.17 | 0.01 | 0.08 | 0.01 |
| BR40 | 72.52 | light grey mudstone | -28.27 | 0.10 | / | 5.74 | 0.21 | 0.02 | 0.15 | 0.01 | 0.07 | 0.02 |
| BR39 | 70.42 | light grey mudstone | -27.26 | 0.14 | / | 6.38 | 0.21 | 0.03 | 0.16 | 0.00 | 0.06 | 0.01 |
| BR38 | 68.03 | light grey mudstone | -28.32 | 0.12 | / | 5.90 | 0.37 | 0.04 | 0.17 | 0.01 | 0.10 | 0.01 |
| BR37 | 65.99 | finely laminated black shale | -27.30 | 0.25 | / | 3.72 | 0.08 | 0.27 | 0.12 | 0.00 | 0.12 | 0.01 |
| BR36 | 63.86 | medium grey mudstone | -27.75 | 0.11 | / | 5.31 | 0.25 | 0.03 | 0.13 | 0.01 | 0.08 | 0.01 |
| BR34 | 61.00 | light grey mudstone | -28.83 | 0.12 | / | 5.83 | 0.58 | 0.03 | 0.13 | 0.00 | 0.13 | 0.01 |
| BR33 | 59.00 | light grey mudstone | -26.31 | 0.21 | / | 5.63 | 0.23 | 0.00 | 0.14 | 0.01 | 0.07 | 0.02 |
| BR32 | 57.00 | light grey mudstone | -28.21 | 0.10 | / | 6.17 | 0.24 | 0.06 | 0.15 | 0.00 | 0.07 | 0.01 |
| BR31 | 55.00 | light grey mudstone | -27.23 | 0.05 | / | 5.20 | 0.20 | 0.04 | 0.09 | 0.00 | 0.06 | 0.01 |
| BR30 | 53.00 | light grey mudstone | -28.59 | 0.06 | / | 4.74 | 0.07 | 0.31 | 0.14 | 0.00 | 0.11 | 0.01 |
| BR29 | 50.80 | light creamy mudstone | -28.23 | 0.04 | -28.78 | 3.06 | 0.42 | 0.04 | 0.04 | 0.01 | 0.17 | 0.01 |
| BR28 | 48.90 | red mudstone | -26.98 | 0.11 | / | 5.66 | 0.08 | 1.39 | 0.32 | 0.00 | 0.32 | 0.00 |
| BR27 | 46.80 | red mudstone | -26.91 | 0.12 | / | 5.37 | 0.09 | 0.03 | 0.37 | 0.01 | 0.09 | 0.01 |
| BR26 | 44.80 | red mudstone | -27.30 | 0.12 | / | 6.15 | 0.12 | 1.28 | 0.29 | 0.00 | 0.28 | 0.00 |
| BR25 | 42.80 | red mudstone | -27.25 | 0.10 | / | 5.68 | 0.00 | 1.24 | 0.30 | 0.00 | 0.27 | 0.00 |
| BR24 | 40.70 | light grey mudstone | -27.26 | 0.09 | / | 3.42 | 0.12 | 0.04 | 0.05 | 0.00 | 0.06 | 0.01 |
| BR23 | 40.20 | red mudstone | -27.03 | 0.11 | / | 5.64 | 0.04 | 1.81 | 0.27 | 0.00 | 0.38 | 0.00 |
| BR22 | 38.20 | red mudstone | -27.20 | 0.13 | / | 5.53 | 0.09 | 1.21 | 0.30 | 0.00 | 0.29 | 0.00 |
| BR21 | 36.20 | red mudstone | -27.32 | 0.11 | / | 5.66 | 0.13 | 1.49 | 0.34 | 0.01 | 0.35 | 0.00 |
| BR20 | 34.10 | red mudstone | -27.45 | 0.11 | / | 6.25 | 0.19 | 0.95 | 0.26 | 0.00 | 0.22 | 0.00 |

| | | | | | | | | | | | | |
|---|---|---|---|---|---|---|---|---|---|---|---|---|
| BR19 | 32.10 | red mudstone | -27.40 | 0.09 | / | 5.78 | 0.01 | 1.07 | 0.31 | 0.01 | 0.24 | 0.00 |
| BR18 | 30.10 | red mudstone | -27.29 | 0.11 | / | 5.83 | 0.00 | 1.99 | 0.36 | 0.01 | 0.41 | 0.00 |
| BR17 | 28.10 | red mudstone | -27.61 | 0.13 | / | 6.10 | 0.04 | 1.25 | 0.30 | 0.01 | 0.26 | 0.00 |
| BR16 | 26.10 | red mudstone | -27.53 | 0.05 | / | 5.65 | 0.09 | 0.81 | 0.24 | 0.01 | 0.20 | 0.00 |
| BR15 | 24.10 | light grey mudstone | -27.44 | 0.08 | / | 5.33 | 0.13 | 0.02 | 0.11 | 0.01 | 0.05 | 0.02 |
| BR14 | 22.10 | light grey mudstone | -27.44 | 0.11 | / | 5.28 | 0.05 | 0.09 | 0.12 | 0.00 | 0.05 | 0.01 |
| BR13 | 20.10 | light grey mudstone | -27.37 | 0.40 | / | 6.25 | 0.01 | 0.12 | 0.14 | 0.00 | 0.04 | 0.02 |
| BR12 | 18.10 | light grey mudstone | -27.51 | 0.08 | / | 5.46 | 0.21 | 0.02 | 0.18 | 0.00 | 0.08 | 0.01 |
| BR11 | 15.10 | light grey mudstone | -27.45 | 0.10 | / | 5.51 | 0.24 | 0.03 | 0.19 | 0.00 | 0.08 | 0.00 |
| BR10 | 13.10 | light grey mudstone | -27.28 | 0.09 | / | 5.85 | 0.24 | 0.02 | 0.19 | 0.00 | 0.08 | 0.01 |
| BR9 | 11.60 | light grey mudstone | -27.62 | 0.11 | -25.38 | 5.38 | 0.19 | 0.04 | 0.37 | 0.01 | 0.11 | 0.01 |
| BR8 | 10.10 | light grey mudstone | -27.87 | 0.10 | / | 5.98 | 0.25 | 0.04 | 0.24 | 0.00 | 0.09 | 0.00 |
| BR7 | 8.60 | light grey mudstone | -27.68 | 0.13 | / | 4.93 | 0.45 | 0.03 | 0.24 | 0.04 | 0.15 | 0.05 |
| BR6 | 7.10 | light grey mudstone | -27.69 | 0.10 | -25.33 | 4.59 | 0.19 | 0.03 | 0.13 | 0.01 | 0.08 | 0.02 |
| BR5 | 5.60 | light grey mudstone | -27.77 | 0.15 | / | 5.02 | 0.31 | 0.03 | 0.18 | 0.01 | 0.10 | 0.02 |
| BR4 | 3.50 | light grey mudstone | -27.78 | 0.10 | / | 6.04 | 0.18 | 0.03 | 0.22 | 0.00 | 0.07 | 0.00 |
| BR3 | 3.00 | light grey mudstone | -27.71 | 0.16 | / | 6.17 | 0.29 | 0.03 | 0.19 | 0.01 | 0.08 | 0.01 |
| BR2 | 1.50 | grey-green mudstone | -28.19 | 0.10 | / | 5.83 | 0.27 | 0.03 | 0.24 | 0.03 | 0.10 | 0.05 |
| BR1 | 0.00 | grey-green mudstone | -28.40 | 0.12 | / | 5.88 | 0.25 | 0.05 | 0.17 | 0.01 | 0.08 | 0.01 |

***Table S2. Major and trace element compositions, and CIA values, for Banwy River samples, east Wales, UK.***

| Name | Height (m) | Lithofacies Description | Al (wt%) | Ca (wt%) | Na (wt%) | K (wt%) | U (ppm) | Mo (ppm) | Re (ppb) | CIA |
|---|---|---|---|---|---|---|---|---|---|---|
| BR148 | 188.59 | finely laminated, medium grey mudstone | 9.03 | 1.60 | 1.12 | 3.17 | 3.93 | 5.31 | 7.97 | 60.88 |
| BR147 | 187.39 | finely laminated, medium grey mudstone | 8.94 | 2.15 | 0.10 | 3.13 | 3.84 | 5.73 | 8.91 | 62.67 |
| BR146 | 186.39 | finely laminated, medium grey mudstone | 8.96 | 2.12 | 0.10 | 3.12 | 3.53 | 2.55 | 5.73 | 62.96 |
| BR145 | 185.39 | finely laminated, medium grey mudstone | 8.54 | 2.47 | 1.04 | 3.02 | 3.17 | 2.69 | 5.21 | 55.77 |
| BR144 | 184.29 | finely laminated, medium grey mudstone | 8.79 | 1.88 | 1.07 | 3.19 | 4.82 | 2.92 | 5.3 | 58.93 |
| BR143 | 183.29 | finely laminated, medium grey mudstone | 8.71 | 2.18 | 1.12 | 3.06 | 3.3 | 2.43 | 5.56 | 57.31 |
| BR142 | 182.19 | finely laminated, medium grey mudstone | 8.63 | 2.04 | 1.12 | 2.97 | 3.42 | 3.24 | 5.82 | 58.07 |
| BR141 | 181.19 | finely laminated, medium grey mudstone | 8.43 | 1.91 | 0.89 | 2.78 | 3.35 | 2.51 | 5.82 | 59.87 |
| BR140 | 180.09 | finely laminated, medium grey mudstone | 8.65 | 2.59 | 1.14 | 3.05 | 3.22 | 2.78 | 5.51 | 55.07 |
| BR139 | 178.99 | finely laminated, medium grey mudstone | 8.24 | 3.85 | 0.84 | 2.76 | 2.81 | 2.95 | 5.13 | 72.49 |
| BR138 | 177.89 | finely laminated, medium grey mudstone | 8.59 | 3.54 | 1.12 | 2.99 | 2.98 | 2.25 | 4.26 | 50.96 |
| BR137 | 176.69 | finely laminated, medium grey mudstone | 8.88 | 3.12 | 1.07 | 3.16 | 2.75 | 2.59 | 3.52 | 53.25 |
| BR136 | 175.59 | finely laminated, medium grey mudstone | 8.61 | 3.56 | 1.10 | 2.98 | 3.04 | 2.34 | 4.73 | 51.01 |
| BR135 | 174.49 | finely laminated, medium grey mudstone | 8.56 | 3.51 | 1.12 | 2.93 | 3.07 | 2.6 | 4.63 | 51.1 |
| BR134 | 173.39 | finely laminated, medium grey mudstone | 8.99 | 2.85 | 1.14 | 3.17 | 3.48 | 1.75 | 5.62 | 54.5 |
| BR133 | 172.19 | finely laminated, medium grey mudstone | 8.60 | 3.90 | 1.03 | 2.98 | 2.87 | 2.67 | 4.2 | 49.84 |
| BR132 | 171.09 | finely laminated, medium grey mudstone | 8.36 | 3.55 | 0.84 | 2.87 | 3.15 | 4.65 | 4.8 | 51.53 |
| BR131 | 169.99 | finely laminated, medium grey mudstone | 8.76 | 2.43 | 0.92 | 2.94 | 3.13 | 4.02 | 5.54 | 57.37 |
| BR130 | 168.99 | finely laminated, medium grey mudstone | 9.13 | 2.65 | 1.09 | 3.28 | 3.15 | 4.16 | 5.12 | 55.63 |
| BR129 | 167.49 | finely laminated, medium grey mudstone | 8.74 | 3.13 | 1.10 | 3.03 | 2.96 | 2.75 | 4.55 | 53.06 |
| BR128 | 166.39 | finely laminated, dark grey mudstone | 9.46 | 2.47 | 1.04 | 3.45 | 3.19 | 2.57 | 4.25 | 57.1 |
| BR127 | 165.29 | finely laminated, dark grey mudstone | 9.06 | 2.08 | 1.11 | 3.13 | 3.17 | 5.28 | 5.63 | 58.65 |

| | | | | | | | | | | |
|---|---|---|---|---|---|---|---|---|---|---|
| BR126 | 164.19 | finely laminated, dark grey mudstone | 8.37 | 1.07 | 0.99 | 2.80 | 2.88 | 1.8 | 4.39 | 64.26 |
| BR125 | 163.09 | finely laminated, dark grey mudstone | 8.61 | 2.40 | 0.90 | 3.18 | 2.68 | 3.78 | 3.94 | 56.51 |
| BR124 | 161.99 | finely laminated, dark grey mudstone | 8.78 | 2.24 | 0.98 | 3.20 | 3.13 | 1.91 | 4.17 | 57.31 |
| BR123 | 160.89 | finely laminated, dark grey mudstone | 8.92 | 1.80 | 1.05 | 3.06 | 3.22 | 2.89 | 5.53 | 60.11 |
| BR122 | 159.89 | finely laminated, dark grey mudstone | 9.12 | 1.48 | 1.02 | 3.30 | 3.34 | 5.18 | 5.7 | 61.82 |
| BR121 | 158.79 | dark grey mudstone, some pyritic laminae | 9.36 | 1.56 | 0.77 | 3.29 | 3.11 | 3.9 | 5.38 | 63.3 |
| BR120 | 157.49 | dark grey mudstone, laminae indistinct | 9.09 | 2.59 | 0.92 | 3.26 | 3.14 | 2.23 | 3.52 | 56.63 |
| BR110 | 156.49 | finely laminated, dark grey mudstone | 8.71 | 3.07 | 0.72 | 3.11 | 2.51 | 2.88 | 3.52 | 54.51 |
| BR109 | 154.89 | finely laminated, dark grey mudstone | 9.09 | 1.85 | 0.79 | 3.11 | 3.45 | 2.94 | 4.68 | 61.4 |
| BR108 | 152.69 | finely laminated, dark grey mudstone | 8.60 | 2.37 | 0.84 | 2.86 | 2.75 | 2.99 | 3.76 | 57.79 |
| BR107 | 150.49 | finely laminated, dark grey mudstone | 8.69 | 2.71 | 0.91 | 2.85 | 2.93 | 3.99 | 4.16 | 56.08 |
| BR106 | 148.84 | finely laminated, dark grey mudstone | 8.42 | 3.42 | 0.77 | 3.04 | 2.14 | 1.48 | 3.89 | 52.07 |
| BR105 | 147.14 | finely laminated, dark grey mudstone | 8.62 | 3.43 | 0.78 | 2.93 | 2.13 | 1.54 | 4.31 | 52.86 |
| BR104 | 145.84 | finely laminated, dark grey mudstone | 9.05 | 2.05 | 0.84 | 3.04 | 3.39 | 3.62 | 4.43 | 60.2 |
| BR103 | 144.74 | finely laminated, dark grey mudstone | 9.03 | 2.88 | 0.82 | 3.08 | 2.5 | 2.31 | 3.53 | 55.88 |
| BR102 | 143.09 | finely laminated, dark grey mudstone | 9.20 | 1.28 | 0.86 | 3.07 | 2.98 | 4.15 | 5.13 | 64.85 |
| BR101 | 141.29 | finely laminated, dark grey mudstone | 8.73 | 3.01 | 0.86 | 2.88 | 2.54 | 2.75 | 4.33 | 54.85 |
| BR100 | 139.79 | finely laminated, dark grey mudstone | 8.82 | 3.03 | 0.90 | 2.96 | 2.47 | 2.61 | 3.89 | 54.66 |
| BR99 | 138.59 | finely laminated, dark grey mudstone | 8.94 | 3.81 | 0.89 | 2.99 | 2.44 | 2.76 | 3.77 | 51.65 |
| BR98 | 137.29 | finely laminated, dark grey mudstone | 8.43 | 3.96 | 0.82 | 2.82 | 2.37 | 2.56 | 3.67 | 50.18 |
| BR97 | 136.69 | finely laminated, dark grey mudstone | 8.53 | 3.48 | 0.86 | 2.82 | 2.6 | 2.91 | 4.23 | 52.35 |
| BR96 | 135.19 | finely laminated, medium grey mudstone | 8.60 | 3.01 | 0.91 | 2.82 | 2.63 | 2.3 | 3.86 | 54.46 |
| BR95 | 133.69 | finely laminated, medium grey mudstone | 8.47 | 3.54 | 0.88 | 2.79 | 2.62 | 3.45 | 3.86 | 51.92 |
| BR94 | 132.59 | finely laminated, medium grey mudstone | 8.72 | 3.24 | 0.90 | 2.86 | 2.51 | 2.46 | 3.54 | 53.66 |
| BR93 | 131.49 | finely laminated, medium grey mudstone | 8.28 | 4.39 | 0.86 | 2.73 | 2.39 | 2.77 | 2.87 | 48.11 |
| BR92 | 130.39 | finely laminated, medium grey mudstone | 7.99 | 4.52 | 0.87 | 2.57 | 2.46 | 3.46 | 3.02 | 47.04 |

| | | | | | | | | | | |
|---|---|---|---|---|---|---|---|---|---|---|
| BR91 | 129.29 | finely laminated, medium grey mudstone | 8.64 | 4.09 | 0.86 | 2.94 | 2.38 | 2.7 | 2.2 | 49.81 |
| BR90 | 127.98 | finely laminated, medium grey mudstone | 8.45 | 3.44 | 0.83 | 2.74 | 2.36 | 2.84 | 2.34 | 52.55 |
| BR89 | 126.68 | finely laminated, medium grey mudstone | 8.89 | 2.62 | 0.91 | 2.88 | 2.42 | 0.57 | 2.25 | 56.93 |
| BR88 | 125.18 | medium grey mudstone | 8.94 | 2.92 | 1.09 | 2.70 | 2.19 | 0.18 | 1.45 | 55.38 |
| BR87 | 123.68 | medium grey mudstone | 8.64 | 2.87 | 0.88 | 2.35 | 2.27 | 0.21 | 1.61 | 56.65 |
| BR86 | 122.78 | medium grey mudstone | 8.77 | 2.37 | 0.91 | 2.31 | 2.24 | 0.17 | 1.53 | 59.75 |
| BR85 | 121.18 | medium grey mudstone | 8.83 | 2.96 | 0.98 | 2.75 | 3.57 | 0.26 | 3.03 | 58.04 |
| BR84 | 119.68 | finely laminated, medium grey mudstone | 9.05 | 2.70 | 0.94 | 2.94 | 3.04 | 2.14 | 3.85 | 56.67 |
| BR83 | 118.18 | finely laminated, medium grey mudstone, small burrows | 8.93 | 3.03 | 0.91 | 2.92 | 2.69 | 0.72 | 1.99 | 55 |
| BR82 | 117.23 | finely laminated, medium grey mudstone | 9.10 | 2.66 | 1.00 | 2.95 | 2.86 | 1.23 | 2.81 | 56.74 |
| BR81 | 116.13 | finely laminated, medium grey mudstone | 9.14 | 2.62 | 0.81 | 2.79 | 2.86 | 1.72 | 2.84 | 58.27 |
| BR80 | 115.03 | finely laminated, medium grey mudstone | 9.43 | 1.62 | 0.81 | 2.90 | 2.79 | 0.99 | 3.08 | 64.17 |
| BR79 | 113.93 | finely laminated, medium grey mudstone | 9.24 | 2.80 | 1.01 | 3.08 | 2.67 | 0.29 | 1.99 | 56.04 |
| BR78 | 112.83 | finely laminated, medium grey mudstone | 8.94 | 2.46 | 0.81 | 2.58 | 2.74 | 0.24 | 2.14 | 59.15 |
| BR77 | 111.93 | finely laminated, medium grey mudstone | 9.55 | 1.24 | 0.87 | 2.88 | 2.55 | 0.3 | 2.13 | 66.48 |
| BR76 | 111.13 | medium grey mudstone with burrows | 8.91 | 1.85 | 0.88 | 2.48 | 2.35 | 0.21 | 1.57 | 62.45 |
| BR75 | 110.13 | finely laminated, medium grey mudstone | 9.04 | 2.55 | 0.86 | 2.57 | 2.42 | 0.22 | 1.55 | 58.81 |
| BR74 | 109.43 | finely laminated, medium grey mudstone | 9.89 | 1.33 | 0.88 | 2.95 | 2.59 | 0.18 | 1.69 | 66.35 |
| BR73 | 108.53 | light grey mudstone with concretions | 9.11 | 1.73 | 0.84 | 2.59 | 2.35 | 0.18 | 1.43 | 63.6 |
| BR72 | 107.93 | finely laminated, medium grey mudstone | 8.95 | 2.91 | 0.79 | 2.73 | 2.48 | 0.22 | 1.73 | 56.61 |
| BR71 | 106.93 | finely laminated, medium grey mudstone | 9.34 | 2.14 | 0.77 | 2.96 | 2.23 | 1.29 | 1.55 | 61 |
| BR69 | 105.03 | light grey mudstone | 3.73 | 5.15 | / | 1.02 | 1.36 | 0.13 | 1.22 | / |
| BR68 | 104.18 | finely laminated, medium grey mudstone | 9.18 | 1.96 | 0.79 | 2.83 | 2.72 | 0.22 | 2.22 | 61.83 |
| BR67 | 103.68 | light grey mudstone | 9.00 | 1.60 | 0.81 | 2.65 | 2.35 | 0.14 | 1.65 | 64.88 |
| BR66 | 102.88 | finely laminated, medium grey mudstone | 9.18 | 1.62 | 0.88 | 2.77 | 2.39 | 0.34 | 1.72 | 63.62 |
| BR65 | 102.18 | light grey mudstone | 9.67 | 1.40 | 0.87 | 3.03 | 2.72 | 0.89 | 2.22 | 65.17 |

| | | | | | | | | | | |
|---|---|---|---|---|---|---|---|---|---|---|
| BR64 | 101.98 | finely laminated, medium grey mudstone | 9.29 | 1.80 | 0.89 | 2.85 | 2.5 | 0.6 | 1.59 | 62.49 |
| BR63 | 101.38 | light grey mudstone | 9.60 | 1.42 | 0.88 | 2.91 | 2.62 | 0.22 | 1.38 | 65.39 |
| BR62 | 100.83 | finely laminated, medium grey mudstone | 9.52 | 1.21 | 1.03 | 3.09 | 2.73 | 0.26 | 2.64 | 64.98 |
| BR61 | 100.13 | light grey mudstone | 9.67 | 0.98 | 1.02 | 2.87 | 2.17 | 0.2 | 1.65 | 67.75 |
| BR60 | 99.43 | light grey mudstone | 9.29 | 1.91 | 0.84 | 2.90 | 2.35 | 0.12 | 1.35 | 61.98 |
| BR59 | 98.78 | light grey mudstone | 9.29 | 1.71 | 0.85 | 2.84 | 2.25 | 0.16 | 1.31 | 63.35 |
| BR58 | 98.23 | medium grey, laminated mudstone with small burrows | 9.77 | 1.11 | 0.88 | 3.11 | 2.27 | 0.22 | 1.19 | 66.91 |
| BR57 | 97.63 | medium grey, laminated mudstone with small burrows | 9.36 | 1.29 | 0.96 | 2.88 | 2.2 | 0.42 | 2.27 | 65.27 |
| BR56 | 97.18 | medium grey, laminated mudstone | 9.92 | 1.01 | 0.94 | 3.11 | 2.37 | 0.36 | 1.53 | 67.59 |
| BR55 | 97.13 | medium grey mudstone | 8.83 | 2.65 | 0.72 | 2.64 | 2.08 | 0.25 | 1.71 | 58.52 |
| BR54 | 96.23 | dark, laminated mudstone | 9.74 | 1.21 | 0.82 | 3.11 | 2.51 | 2.66 | 3.21 | 66.56 |
| BR53 | 95.83 | dark, laminated mudstone | 9.60 | 1.28 | 0.85 | 3.03 | 2.32 | 1.55 | 3.48 | 65.79 |
| BR52 | 95.43 | dark, laminated mudstone | 9.46 | 1.39 | 0.92 | 3.06 | 2.48 | 1.47 | 3.35 | 64.42 |
| BR51 | 93.63 | medium grey, laminated mudstone with small burrows | 9.57 | 1.68 | 0.82 | 2.99 | 2.56 | 0.81 | 1.88 | 63.82 |
| BR50 | 91.63 | medium grey, laminated mudstone with small burrows | 9.08 | 1.45 | 0.86 | 2.70 | 2.16 | 0.26 | 1.79 | 64.9 |
| BR49 | 90.43 | medium grey mudstone | 9.48 | 1.53 | 0.79 | 2.81 | 1.95 | 0.16 | 1.41 | 65.46 |
| BR48 | 88.43 | medium grey mudstone | 9.70 | 1.42 | 1.11 | 2.99 | 2.45 | 0.86 | 1.27 | 64.18 |
| BR47 | 86.43 | dark grey mudstone | 9.79 | 1.19 | 0.89 | 3.19 | 3.54 | 1.33 | 1.9 | 66.12 |
| BR46 | 84.43 | medium grey mudstone | 10.03 | 0.75 | 0.99 | 3.09 | 2.02 | 0.16 | 1.5 | 73.62 |
| BR45 | 82.43 | laminated medium grey mudstone | 9.80 | 0.93 | 0.94 | 3.12 | 3.26 | 0.33 | 1.61 | 67.81 |
| BR44 | 80.37 | finely laminated black shale | 9.99 | 0.66 | 1.04 | 3.05 | 2.31 | 0.29 | 1.17 | 67.48 |
| BR43 | 78.72 | light grey mudstone | 10.03 | 0.27 | 0.73 | 3.57 | 2.37 | 0.48 | 1.17 | 69.16 |
| BR44 | 76.72 | light grey mudstone | 9.95 | 0.69 | 0.87 | 2.89 | 2.01 | 0.12 | 1.09 | 69.95 |
| BR41 | 74.62 | light grey mudstone | 10.04 | 0.36 | 0.90 | 3.12 | 2.02 | 0.15 | 1.66 | 68.89 |
| BR40 | 72.52 | light grey mudstone | 10.62 | 0.25 | 0.85 | 3.39 | 2.11 | 0.14 | 1.43 | 69.67 |
| BR39 | 70.42 | light grey mudstone | 10.17 | 0.52 | 0.73 | 2.83 | 1.9 | 0.12 | 0.92 | 72.21 |

| | | | | | | | | | | |
|---|---|---|---|---|---|---|---|---|---|---|
| BR38 | 68.03 | light grey mudstone | 9.21 | 0.83 | 0.83 | 2.55 | 1.71 | 0.14 | 1.22 | 69.95 |
| BR37 | 65.99 | finely laminated black shale | 10.63 | 0.20 | 0.96 | 3.59 | 2.8 | 0.2 | 2.14 | 67.86 |
| BR36 | 63.86 | medium grey mudstone | 9.55 | 0.61 | 1.00 | 2.72 | 1.71 | 0.19 | 1.32 | 68.16 |
| BR34 | 61.00 | light grey mudstone | 9.80 | 0.74 | 0.82 | 2.95 | 1.79 | 0.16 | 1.36 | 69.95 |
| BR33 | 59.00 | light grey mudstone | 10.22 | 0.48 | 0.76 | 3.08 | 1.72 | 0.2 | 0.75 | 70.99 |
| BR32 | 57.00 | light grey mudstone | 10.49 | 0.40 | 0.69 | 3.03 | 1.74 | 0.11 | 1.3 | 72.57 |
| BR31 | 55.00 | light grey mudstone | 10.65 | 0.48 | 0.82 | 3.23 | 1.98 | 0.11 | 1.23 | 70.67 |
| BR30 | 53.00 | light grey mudstone | 12.02 | 0.19 | 0.81 | 4.03 | 2.34 | 0.21 | 1.34 | 70.52 |
| BR29 | 50.80 | light creamy mudstone | 10.53 | 1.45 | 0.84 | 3.76 | 3.55 | 0.22 | 1.24 | 64.73 |
| BR28 | 48.90 | red mudstone | 10.80 | 0.39 | 0.79 | 3.81 | 1.99 | 0.32 | 1.32 | 69.23 |
| BR27 | 46.80 | red mudstone | 10.45 | 0.43 | 0.77 | 3.72 | 2.03 | 0.36 | 0.97 | 69.05 |
| BR26 | 44.80 | red mudstone | 11.20 | 0.20 | 1.03 | 3.48 | 1.99 | 0.46 | 1.43 | 68.63 |
| BR25 | 42.80 | red mudstone | 10.98 | 0.46 | 0.79 | 3.74 | 2.28 | 0.49 | 0.86 | 69.77 |
| BR24 | 40.70 | light grey mudstone | 10.39 | 0.24 | 0.85 | 3.20 | 2.32 | 0.18 | 1.13 | 69.85 |
| BR23 | 40.20 | red mudstone | 12.13 | 0.21 | 0.66 | 4.18 | 1.69 | 0.43 | 1.15 | 71.71 |
| BR22 | 38.20 | red mudstone | 10.22 | 0.28 | 0.98 | 3.54 | 2.14 | 0.46 | 1.25 | 66.97 |
| BR21 | 36.20 | red mudstone | 10.87 | 0.18 | 0.71 | 3.63 | 2.38 | 0.45 | 1.14 | 70.77 |
| BR20 | 34.10 | red mudstone | 12.33 | 0.25 | 0.83 | 3.99 | 2.36 | 0.27 | 1.56 | 70.99 |
| BR19 | 32.10 | red mudstone | 11.04 | 0.31 | 0.70 | 3.79 | 2.42 | 0.43 | 1.01 | 70.72 |
| BR18 | 30.10 | red mudstone | 11.07 | 0.20 | 0.64 | 3.75 | 1.98 | 0.36 | 0.86 | 71.56 |
| BR17 | 28.10 | red mudstone | 11.36 | 0.21 | 0.67 | 3.97 | 2.29 | 0.43 | 1.01 | 70.92 |
| BR16 | 26.10 | red mudstone | 11.67 | 0.20 | 0.59 | 3.88 | 2.05 | 0.29 | 1.15 | 72.65 |
| BR15 | 24.10 | light grey mudstone | 11.16 | 0.26 | / | 3.34 | 2.29 | 0.18 | 0.9 | / |
| BR14 | 22.10 | light grey mudstone | 10.78 | 0.21 | 1.30 | 3.28 | 1.92 | 0.23 | 1.21 | 65.89 |
| BR13 | 20.10 | light grey mudstone | 11.95 | 0.24 | / | 3.43 | 1.94 | 0.14 | 0.75 | / |
| BR12 | 18.10 | light grey mudstone | 11.61 | 0.26 | 0.84 | 3.39 | 2.27 | 0.16 | 1.01 | 71.63 |

| | | | | | | | | | | |
|---|---|---|---|---|---|---|---|---|---|---|
| BR11 | 15.10 | light grey mudstone | 11.51 | 0.21 | 0.75 | 3.29 | 1.96 | 0.13 | 1.15 | 72.81 |
| BR10 | 13.10 | light grey mudstone | 11.66 | 0.53 | 0.91 | 4.38 | 2.33 | 0.46 | 1.39 | 67.86 |
| BR9 | 11.60 | light grey mudstone | 11.33 | 0.25 | 0.68 | 3.48 | 2.11 | 0.36 | 0.87 | 72.54 |
| BR8 | 10.10 | light grey mudstone | 10.27 | 0.49 | 1.14 | 3.19 | 1.87 | 0.13 | 1.57 | 66.6 |
| BR7 | 8.60 | light grey mudstone | 10.77 | 0.26 | 0.81 | 3.51 | 2.06 | 0.37 | 1.53 | 70 |
| BR6 | 7.10 | light grey mudstone | 11.29 | 0.19 | 0.68 | 3.45 | 2.27 | 0.12 | 1.28 | 72.53 |
| BR5 | 5.60 | light grey mudstone | 11.91 | 0.25 | 0.81 | 3.86 | 2.24 | 0.59 | 0.97 | 70.88 |
| BR4 | 3.50 | light grey mudstone | 11.59 | 0.20 | 0.61 | 3.61 | 2.19 | 0.17 | 1.36 | 73.25 |
| BR3 | 3.00 | light grey mudstone | 11.88 | 0.20 | 0.94 | 3.72 | 2.29 | 0.12 | 1.3 | 70.06 |
| BR2 | 1.50 | grey-green mudstone | 11.01 | 0.28 | 0.67 | 3.28 | 2.32 | 0.1 | 1.62 | 72.85 |
| BR1 | 0.00 | grey-green mudstone | 10.27 | 0.22 | 0.71 | 3.33 | 2.04 | 0.13 | 1.15 | 70.77 |

*Table S3. Organic C and pyrite S isotopes, and Fe speciation contents, in Ashgill Beck samples, Lake District, UK.*

| Name | Height (m) | Lithofacies Description | $\delta^{13}C_{org}$ (‰) | TOC (wt%) | $\delta^{34}S_{py}$ (‰) | $Fe_T$ (wt%) | $Fe_{carb}$ (wt%) | $Fe_{ox}$ (wt%) | $Fe_{mag}$ (wt%) | $Fe_{py}$ (wt%) | $Fe_{HR}/Fe_T$ | $Fe_{py}/Fe_{HR}$ |
|---|---|---|---|---|---|---|---|---|---|---|---|---|
| AG50 | 64.50 | finely laminated, medium grey mudstone | -28.09 | 0.35 | -21.88 | 4.20 | 0.52 | 0.07 | 0.04 | 0.96 | 0.38 | 0.61 |
| AG49 | 62.70 | finely laminated, medium grey mudstone | -27.85 | 0.35 | -21.54 | 4.25 | 0.51 | 0.04 | 0.05 | 0.93 | 0.36 | 0.60 |
| AG48 | 61.20 | finely laminated, medium grey mudstone | -27.79 | 0.38 | -22.17 | 4.27 | 0.45 | 0.20 | 0.07 | 0.68 | 0.33 | 0.49 |
| AG47 | 59.20 | finely laminated, medium grey mudstone | -27.79 | 0.41 | -21.66 | 4.41 | 0.64 | 0.45 | 0.05 | 0.65 | 0.40 | 0.36 |
| AG46 | 58.00 | finely laminated, medium grey mudstone | -28.55 | 0.39 | -22.37 | 4.68 | 0.50 | 0.04 | 0.03 | 1.18 | 0.37 | 0.67 |
| AG45 | 56.10 | finely laminated, medium grey mudstone | -27.72 | 0.39 | -22.19 | 4.37 | 0.53 | 0.13 | 0.05 | 0.89 | 0.36 | 0.56 |
| AG44 | 54.70 | finely laminated, medium grey mudstone | -28.03 | 0.36 | -20.67 | 4.30 | 0.42 | 0.04 | 0.05 | 1.01 | 0.35 | 0.66 |
| AG43 | 53.20 | finely laminated, medium grey mudstone | -27.91 | 0.36 | -20.86 | 4.32 | 0.31 | 0.24 | 0.03 | 0.59 | 0.27 | 0.50 |
| AG42 | 51.40 | finely laminated, medium grey mudstone | -27.49 | 0.52 | -16.30 | 3.73 | 0.32 | 0.50 | 0.10 | 0.06 | 0.26 | 0.06 |
| AG41 | 49.80 | finely laminated, medium grey mudstone | -27.42 | 0.48 | -20.73 | 4.54 | 0.41 | 0.07 | 0.07 | 0.95 | 0.33 | 0.64 |
| AG40 | 48.25 | finely laminated, medium grey mudstone | -27.56 | 0.44 | -21.24 | 4.69 | 0.47 | 0.32 | 0.06 | 0.83 | 0.36 | 0.49 |
| AG39 | 46.80 | finely laminated, medium grey mudstone | -27.45 | 0.44 | -20.00 | 4.42 | 0.45 | 0.05 | 0.07 | 1.02 | 0.36 | 0.64 |
| AG38 | 44.10 | finely laminated, medium grey mudstone | -27.84 | 0.43 | -20.71 | 4.43 | 0.41 | 0.05 | 0.04 | 0.95 | 0.33 | 0.65 |
| AG37 | 42.00 | finely laminated, medium grey mudstone | -27.60 | 0.47 | -21.49 | 4.48 | 0.45 | 0.22 | 0.06 | 0.54 | 0.28 | 0.43 |
| AG36 | 40.20 | finely laminated, medium grey mudstone | -27.37 | 0.49 | -18.98 | 4.44 | 0.48 | 0.16 | 0.06 | 0.87 | 0.35 | 0.56 |
| AG35 | 38.80 | very finely laminated, dark grey mudstone | -27.09 | 0.58 | -18.81 | 4.31 | 0.39 | 0.16 | 0.06 | 0.80 | 0.33 | 0.56 |
| AG34 | 37.30 | very finely laminated, dark grey mudstone | -27.50 | 0.52 | -21.04 | 4.73 | 0.49 | 0.26 | 0.05 | 0.92 | 0.36 | 0.54 |
| AG33 | 35.50 | very finely laminated, dark grey mudstone | -27.27 | 0.51 | -20.40 | 4.60 | 0.42 | 0.28 | 0.07 | 0.74 | 0.33 | 0.49 |
| AG32 | 34.20 | very finely laminated, dark grey mudstone | -26.50 | 0.73 | -19.08 | 4.29 | 0.49 | 0.30 | 0.04 | 1.00 | 0.42 | 0.55 |
| AG31 | 32.90 | very finely laminated, dark grey mudstone | -26.47 | 0.56 | -15.45 | 4.61 | 0.51 | 0.09 | 0.05 | 1.05 | 0.37 | 0.62 |
| AG30 | 30.40 | very finely laminated, dark grey mudstone | -26.56 | 0.75 | -17.06 | 5.08 | 0.43 | 0.48 | 0.05 | 1.15 | 0.42 | 0.55 |
| AG29 | 29.30 | very finely laminated, dark grey mudstone | -26.26 | 0.69 | -14.21 | 4.43 | 0.34 | 0.57 | 0.07 | 0.50 | 0.33 | 0.34 |

| | | | | | | | | | | | | |
|---|---|---|---|---|---|---|---|---|---|---|---|---|
| AG28 | 28.25 | very finely laminated, dark grey mudstone | -26.28 | 0.64 | -18.04 | 4.84 | 0.43 | 0.33 | 0.05 | 0.94 | 0.36 | 0.53 |
| AG27 | 27.30 | very finely laminated, dark grey mudstone | -26.58 | 0.60 | -15.68 | 4.72 | 0.52 | 0.38 | 0.07 | 1.03 | 0.43 | 0.51 |
| AG26 | 25.87 | very finely laminated, dark grey mudstone | -26.15 | 0.54 | -16.82 | 4.76 | 0.41 | 0.22 | 0.06 | 0.73 | 0.30 | 0.51 |
| AG25 | 24.70 | very finely laminated, dark grey mudstone | -26.53 | 0.74 | -18.57 | 4.73 | 0.45 | 0.20 | 0.05 | 1.14 | 0.39 | 0.62 |
| AG24 | 23.80 | very finely laminated, dark grey mudstone | -26.16 | 0.64 | -18.72 | 4.81 | 0.47 | 0.79 | 0.06 | 0.59 | 0.40 | 0.31 |
| AG23 | 22.60 | very finely laminated, dark grey mudstone | -25.88 | 0.99 | -15.28 | 4.84 | 0.30 | 0.78 | 0.07 | 0.55 | 0.35 | 0.33 |
| AG22 | 21.50 | very finely laminated, dark grey mudstone | -26.04 | 0.71 | -13.91 | 4.49 | 0.43 | 0.38 | 0.03 | 1.27 | 0.47 | 0.60 |
| AG21 | 20.51 | very finely laminated, dark grey mudstone | -26.22 | 0.63 | -13.24 | 4.36 | 0.52 | 0.13 | 0.06 | 1.00 | 0.39 | 0.59 |
| AG20 | 19.40 | very finely laminated, dark grey mudstone | -26.17 | 0.83 | -14.11 | 4.58 | 0.45 | 0.41 | 0.03 | 0.95 | 0.40 | 0.52 |
| AG19 | 18.20 | very finely laminated, dark grey mudstone | -26.36 | 0.60 | -16.19 | 4.60 | 0.45 | 0.23 | 0.05 | 0.94 | 0.36 | 0.56 |
| AG18 | 16.80 | very finely laminated, dark grey mudstone | -26.54 | 0.67 | -16.77 | 4.69 | 0.47 | 0.30 | 0.07 | 0.80 | 0.35 | 0.49 |
| AG17 | 15.30 | very finely laminated, dark grey mudstone | -26.23 | 0.56 | -16.17 | 4.65 | 0.42 | 0.23 | 0.07 | 0.95 | 0.36 | 0.57 |
| AG16 | 14.30 | very finely laminated, dark grey mudstone | -26.54 | 0.73 | -17.52 | 4.70 | 0.48 | 0.36 | 0.06 | 0.93 | 0.39 | 0.51 |
| AG15 | 13.50 | very finely laminated, dark grey mudstone | -26.29 | 0.74 | -16.32 | 4.90 | 0.36 | 0.65 | 0.14 | 0.42 | 0.32 | 0.27 |
| AG14 | 12.30 | very finely laminated, dark grey mudstone | -26.25 | 0.73 | -14.55 | 4.51 | 0.44 | 0.54 | 0.06 | 0.60 | 0.36 | 0.37 |
| AG13 | 11.20 | very finely laminated, dark grey mudstone | -26.22 | 0.63 | -13.74 | 4.23 | 0.45 | 0.28 | 0.06 | 0.54 | 0.31 | 0.41 |
| AG12 | 10.10 | very finely laminated, dark grey mudstone | -26.86 | 0.54 | -17.35 | 4.07 | 0.48 | 0.17 | 0.07 | 0.84 | 0.38 | 0.54 |
| AG11 | 8.90 | very finely laminated, dark grey mudstone | -26.55 | 0.54 | -18.44 | 4.26 | 0.39 | 0.14 | 0.03 | 0.92 | 0.35 | 0.62 |
| AG10 | 7.62 | very finely laminated, dark grey mudstone | -26.55 | 0.48 | -17.60 | 4.30 | 0.48 | 0.06 | 0.06 | 0.87 | 0.34 | 0.59 |
| AG9 | 6.13 | very finely laminated, dark grey mudstone | -26.84 | 0.53 | -17.20 | 4.23 | 0.49 | 0.32 | 0.07 | 0.67 | 0.37 | 0.43 |
| AG8 | 4.86 | very finely laminated, dark grey mudstone | -27.12 | 0.57 | -15.55 | 4.63 | 0.52 | 0.31 | 0.06 | 0.79 | 0.36 | 0.47 |
| AG7 | 4.36 | very finely laminated, dark grey mudstone | -27.28 | 0.46 | -20.45 | 4.83 | 0.42 | 0.07 | 0.06 | 1.17 | 0.35 | 0.68 |
| AG6 | 4.10 | very finely laminated, dark grey mudstone | -27.16 | 0.43 | -20.29 | 4.77 | 0.24 | 0.05 | 0.06 | 1.13 | 0.31 | 0.76 |
| AG5 | 4.00 | very finely laminated, dark grey mudstone | -27.10 | 0.41 | -22.24 | 4.59 | 0.24 | 0.03 | 0.07 | 1.11 | 0.32 | 0.77 |
| AG4 | 3.25 | light grey mudstone | -27.62 | 0.12 | / | 4.52 | 0.30 | 0.20 | 0.16 | 0.00 | 0.15 | 0.00 |
| AG3 | 2.63 | light grey mudstone | -27.30 | 0.08 | -29.20 | 5.53 | 0.22 | 0.10 | 0.09 | 0.05 | 0.08 | 0.11 |

| | | | | | | | | | | | | |
|---|---|---|---|---|---|---|---|---|---|---|---|---|
| AG2 | 1.65 | light grey mudstone | -27.63 | 0.25 | / | 4.86 | 0.24 | 0.08 | 0.10 | 0.00 | 0.09 | 0.01 |
| AG1 | 0.00 | light grey mudstone | -27.79 | 0.15 | -29.10 | 5.08 | 0.18 | 0.03 | 0.09 | 0.00 | 0.06 | 0.01 |

*Table S4. Major and trace element compositions of Ashgill Beck samples, Lake District, UK.*

| Name | Height (m) | Lithofacies Description | Al (wt%) | Ca (wt%) | Na (wt%) | K (wt%) | U (ppm) | Mo (ppm) | Re (ppb) |
|---|---|---|---|---|---|---|---|---|---|
| AG50 | 64.50 | finely laminated, medium grey mudstone | 7.66 | 3.40 | 1.13 | 2.60 | 3.67 | 5.50 | 7.36 |
| AG49 | 62.70 | finely laminated, medium grey mudstone | 8.15 | 2.75 | 1.15 | 2.82 | 3.71 | 5.32 | 7.27 |
| AG48 | 61.20 | finely laminated, medium grey mudstone | 8.43 | 0.99 | 1.13 | 2.92 | 3.80 | 7.08 | 8.02 |
| AG47 | 59.20 | finely laminated, medium grey mudstone | 8.43 | 0.84 | 1.10 | 2.87 | 3.67 | 9.45 | 8.30 |
| AG46 | 58.00 | finely laminated, medium grey mudstone | 8.93 | 0.44 | 1.14 | 3.07 | 3.95 | 5.97 | 7.63 |
| AG45 | 56.10 | finely laminated, medium grey mudstone | 8.24 | 2.01 | 1.10 | 2.78 | 3.71 | 6.35 | 8.28 |
| AG44 | 54.70 | finely laminated, medium grey mudstone | 8.23 | 3.28 | 1.06 | 2.77 | 3.46 | 5.98 | 7.36 |
| AG43 | 53.20 | finely laminated, medium grey mudstone | 9.12 | 0.19 | 1.03 | 3.05 | 3.63 | 4.72 | 7.29 |
| AG42 | 51.40 | finely laminated, medium grey mudstone | 9.53 | 0.19 | 1.25 | 3.27 | 3.65 | 1.41 | 12.35 |
| AG41 | 49.80 | finely laminated, medium grey mudstone | 9.23 | 0.22 | 1.19 | 3.05 | 3.91 | 5.00 | 7.30 |
| AG40 | 48.25 | finely laminated, medium grey mudstone | 8.61 | 1.14 | 1.13 | 2.84 | 3.32 | 4.66 | 7.53 |
| AG39 | 46.80 | finely laminated, medium grey mudstone | 9.15 | 0.72 | 1.16 | 3.12 | 3.76 | 6.04 | 8.02 |
| AG38 | 44.10 | finely laminated, medium grey mudstone | 9.13 | 1.50 | 1.18 | 2.97 | 3.75 | 5.46 | 7.20 |
| AG37 | 42.00 | finely laminated, medium grey mudstone | 8.70 | 0.80 | 1.14 | 2.84 | 3.34 | 5.07 | 6.99 |
| AG36 | 40.20 | finely laminated, medium grey mudstone | 8.91 | 1.52 | 1.19 | 2.97 | 3.76 | 5.42 | 7.41 |
| AG35 | 38.80 | very finely laminated, dark grey mudstone | 9.14 | 2.60 | 1.12 | 3.09 | 2.38 | 1.01 | 2.54 |
| AG34 | 37.30 | very finely laminated, dark grey mudstone | 8.55 | 1.11 | 1.18 | 2.77 | 3.50 | 5.44 | 8.16 |
| AG33 | 35.50 | very finely laminated, dark grey mudstone | 9.25 | 0.19 | 1.19 | 2.99 | 3.84 | 6.08 | 8.34 |
| AG32 | 34.20 | very finely laminated, dark grey mudstone | 7.97 | 3.10 | 0.97 | 2.68 | 2.82 | 2.00 | 4.99 |
| AG31 | 32.90 | very finely laminated, dark grey mudstone | 8.34 | 1.23 | 1.07 | 2.69 | 2.55 | 1.97 | 5.71 |
| AG30 | 30.40 | very finely laminated, dark grey mudstone | 8.86 | 0.52 | 1.15 | 2.85 | 2.95 | 4.20 | 7.67 |
| AG29 | 29.30 | very finely laminated, dark grey mudstone | 8.66 | 1.60 | 1.00 | 2.93 | 2.78 | 2.08 | 4.33 |

| | | | | | | | | | |
|---|---|---|---|---|---|---|---|---|---|
| AG28 | 28.25 | very finely laminated, dark grey mudstone | 8.55 | 1.23 | 1.11 | 2.73 | 2.85 | 2.63 | 6.45 |
| AG27 | 27.30 | very finely laminated, dark grey mudstone | 8.06 | 2.74 | 1.04 | 2.61 | 2.80 | 3.49 | 6.53 |
| AG26 | 25.87 | very finely laminated, dark grey mudstone | 9.17 | 2.44 | 1.24 | 3.00 | 3.18 | 4.75 | 8.00 |
| AG25 | 24.70 | very finely laminated, dark grey mudstone | 8.63 | 0.88 | 1.09 | 2.79 | 3.40 | 4.96 | 8.63 |
| AG24 | 23.80 | very finely laminated, dark grey mudstone | 8.73 | 2.37 | 1.01 | 2.97 | 3.11 | 4.44 | 6.76 |
| AG23 | 22.60 | very finely laminated, dark grey mudstone | 9.29 | 0.15 | 1.02 | 3.14 | 3.02 | 2.95 | 4.96 |
| AG22 | 21.50 | very finely laminated, dark grey mudstone | 8.51 | 2.05 | 0.97 | 3.05 | 2.81 | 2.88 | 3.86 |
| AG21 | 20.51 | very finely laminated, dark grey mudstone | 7.86 | 4.25 | 0.84 | 2.70 | 2.58 | 2.43 | 3.74 |
| AG20 | 19.40 | very finely laminated, dark grey mudstone | 8.33 | 0.74 | 1.00 | 2.79 | 2.88 | 2.73 | 4.41 |
| AG19 | 18.20 | very finely laminated, dark grey mudstone | 8.37 | 2.06 | 1.01 | 2.81 | 2.90 | 2.66 | 4.71 |
| AG18 | 16.80 | very finely laminated, dark grey mudstone | 8.62 | 1.68 | 0.98 | 2.82 | 2.70 | 2.32 | 4.96 |
| AG17 | 15.30 | very finely laminated, dark grey mudstone | 8.49 | 2.04 | 0.98 | 2.88 | 2.76 | 3.30 | 5.24 |
| AG16 | 14.30 | very finely laminated, dark grey mudstone | 8.44 | 1.15 | 1.02 | 2.81 | 2.88 | 3.56 | 5.35 |
| AG15 | 13.50 | very finely laminated, dark grey mudstone | 8.99 | 0.17 | 1.05 | 3.04 | 2.77 | 2.68 | 4.38 |
| AG14 | 12.30 | very finely laminated, dark grey mudstone | 8.48 | 1.33 | 0.92 | 2.95 | 2.28 | 1.90 | 3.06 |
| AG13 | 11.20 | very finely laminated, dark grey mudstone | 8.10 | 3.19 | 0.94 | 2.84 | 2.24 | 1.91 | 2.53 |
| AG12 | 10.10 | very finely laminated, dark grey mudstone | 8.16 | 3.34 | 0.92 | 2.88 | 2.63 | 2.09 | 3.57 |
| AG11 | 8.90 | very finely laminated, dark grey mudstone | 7.83 | 3.93 | 0.89 | 2.74 | 2.82 | 2.53 | 3.36 |
| AG10 | 7.62 | very finely laminated, dark grey mudstone | 7.95 | 3.98 | 0.89 | 2.75 | 2.39 | 2.07 | 3.09 |
| AG9 | 6.13 | very finely laminated, dark grey mudstone | 7.41 | 3.46 | 0.82 | 2.65 | 2.22 | 1.71 | 2.54 |
| AG8 | 4.86 | very finely laminated, dark grey mudstone | 8.06 | 2.76 | 0.64 | 2.98 | 2.38 | 3.08 | 2.02 |
| AG7 | 4.36 | very finely laminated, dark grey mudstone | 8.40 | 0.87 | 0.78 | 3.05 | 3.02 | 3.13 | 3.69 |
| AG6 | 4.10 | very finely laminated, dark grey mudstone | 8.60 | 0.59 | 0.83 | 3.12 | 3.05 | 1.34 | 3.03 |
| AG5 | 4.00 | very finely laminated, dark grey mudstone | 9.84 | 0.77 | 1.10 | 3.41 | 3.20 | 0.38 | 1.61 |
| AG4 | 3.25 | light grey mudstone | 8.90 | 0.42 | 0.89 | 3.41 | 3.04 | 1.19 | 3.08 |
| AG3 | 2.63 | light grey mudstone | 8.88 | 0.18 | 0.98 | 2.83 | 2.03 | 0.15 | 1.47 |

| | | | | | | | | | |
|---|---|---|---|---|---|---|---|---|---|
| AG2 | 1.65 | light grey mudstone | 8.79 | 0.76 | 1.05 | 2.94 | 2.45 | 0.62 | 1.65 |
| AG1 | 0.00 | light grey mudstone | 9.40 | 2.22 | 1.02 | 3.18 | 1.88 | 0.05 | 1.18 |

***Table S5. Detailed geochemical information for U and Mo isotope samples in the Banwy River section and Ashgill Beck section, UK.***

| Name | Section | Depth | TOC (wt%) | $Fe_{HR}/Fe_T$ | $Fe_{py}/Fe_{HR}$ | Re (ppb) | U (ppm) | Mo (ppm) | $\delta^{238}U$ (‰) | 2 s.e. (‰) | $\delta^{98}Mo$ (‰) | 2 s.e. (‰) | Redox interpretation |
|---|---|---|---|---|---|---|---|---|---|---|---|---|---|
| BR145 | BR | 185.39 | 0.69 | 0.48 | 0.47 | 5.21 | 3.17 | 2.69 | -0.35 | 0.07 | 0.45 | 0.03 | ferruginous |
| BR141 | BR | 181.19 | 0.59 | 0.43 | 0.47 | 5.82 | 3.35 | 2.51 | -0.30 | 0.06 | 0.10 | 0.03 | ferruginous |
| BR137 | BR | 176.69 | 0.76 | 0.49 | 0.49 | 3.52 | 2.75 | 2.59 | -0.32 | 0.06 | -1.29 | 0.07 | ferruginous |
| BR135 | BR | 174.49 | 0.61 | 0.52 | 0.52 | 4.63 | 3.07 | 2.60 | -0.32 | 0.06 | 0.44 | 0.03 | ferruginous |
| BR132 | BR | 171.09 | 0.57 | 0.55 | 0.54 | 4.80 | 3.15 | 4.65 | -0.36 | 0.06 | 0.95 | 0.04 | ferruginous |
| BR128 | BR | 166.39 | 0.59 | 0.44 | 0.51 | 4.25 | 3.19 | 2.57 | -0.26 | 0.06 | 0.08 | 0.04 | ferruginous |
| BR124 | BR | 161.99 | 0.65 | 0.45 | 0.50 | 4.17 | 3.13 | 1.91 | -0.32 | 0.07 | 0.26 | 0.04 | ferruginous |
| BR122 | BR | 159.89 | 0.80 | 0.53 | 0.54 | 5.70 | 3.34 | 5.18 | -0.23 | 0.06 | 0.50 | 0.03 | weakly euxinic |
| BR110 | BR | 156.49 | 0.72 | 0.46 | 0.61 | 3.52 | 2.51 | 2.88 | -0.33 | 0.06 | 0.76 | 0.03 | weakly euxinic |
| BR107 | BR | 150.49 | 0.79 | 0.44 | 0.63 | 4.16 | 2.93 | 3.99 | -0.28 | 0.05 | 0.48 | 0.03 | weakly euxinic |
| BR106 | BR | 148.84 | 0.60 | 0.40 | 0.60 | 3.89 | 2.14 | 1.48 | -0.30 | 0.06 | -0.21 | 0.02 | weakly euxinic |
| BR104 | BR | 145.84 | 0.76 | 0.41 | 0.68 | 4.43 | 3.39 | 3.62 | -0.30 | 0.05 | 0.40 | 0.04 | weakly euxinic |
| BR103 | BR | 144.74 | 0.64 | 0.60 | 0.79 | 3.53 | 2.50 | 2.31 | -0.31 | 0.07 | 0.14 | 0.05 | weakly euxinic |
| BR101 | BR | 141.29 | 0.78 | 0.41 | 0.64 | 4.33 | 2.54 | 2.75 | -0.25 | 0.06 | 0.13 | 0.04 | weakly euxinic |
| BR98 | BR | 137.29 | 0.72 | 0.45 | 0.62 | 3.67 | 2.37 | 2.56 | -0.33 | 0.07 | 0.90 | 0.05 | weakly euxinic |
| BR95 | BR | 133.69 | 0.65 | 0.45 | 0.65 | 3.86 | 2.62 | 3.45 | -0.23 | 0.08 | 0.42 | 0.03 | weakly euxinic |
| BR90 | BR | 127.98 | 0.60 | 0.42 | 0.61 | 2.34 | 2.36 | 2.84 | -0.29 | 0.06 | 0.08 | 0.04 | weakly euxinic |
| BR71 | BR | 106.93 | 0.29 | 0.19 | 0.20 | 1.55 | 2.23 | 1.29 | -0.30 | 0.06 | 0.60 | 0.04 | ferruginous |
| BR53 | BR | 95.83 | 0.38 | 0.17 | 0.24 | 3.48 | 2.32 | 1.55 | -0.28 | 0.05 | 0.45 | 0.03 | ferruginous |
| BR43 | BR | 78.72 | 0.10 | 0.11 | 0.11 | 1.17 | 2.37 | 0.48 | -0.36 | 0.06 | / | / | dysoxic |
| BR38 | BR | 68.03 | 0.12 | 0.10 | 0.01 | 1.22 | 1.71 | 0.14 | -0.35 | 0.06 | / | / | dysoxic |
| BR25 | BR | 42.8 | 0.10 | 0.27 | 0.00 | 0.86 | 2.28 | 0.49 | -0.34 | 0.07 | -1.52 | 0.04 | oxic |

| | | | | | | | | | | | | | |
|---|---|---|---|---|---|---|---|---|---|---|---|---|---|
| BR22 | BR | 38.2 | 0.13 | 0.29 | 0.00 | 1.25 | 2.14 | 0.46 | -0.33 | 0.07 | -1.54 | 0.05 | oxic |
| BR17 | BR | 28.1 | 0.13 | 0.26 | 0.00 | 1.01 | 2.29 | 0.43 | -0.40 | 0.07 | -1.44 | 0.06 | oxic |
| BR12 | BR | 18.1 | 0.08 | 0.08 | 0.01 | 1.01 | 2.27 | 0.16 | -0.34 | 0.06 | / | / | dysoxic |
| BR9 | BR | 11.6 | 0.11 | 0.11 | 0.01 | 0.87 | 2.11 | 0.36 | -0.31 | 0.06 | -2.39 | 0.04 | dysoxic |
| BR6 | BR | 7.1 | 0.10 | 0.08 | 0.02 | 1.28 | 2.27 | 0.12 | -0.31 | 0.07 | / | / | dysoxic |
| AG50 | AB | 64.5 | 0.35 | 0.38 | 0.61 | 7.36 | 3.67 | 5.50 | -0.18 | 0.05 | 0.24 | 0.08 | weakly euxinic |
| AG48 | AB | 61.2 | 0.38 | 0.33 | 0.49 | 8.02 | 3.80 | 7.08 | -0.20 | 0.05 | 0.49 | 0.08 | ferruginous |
| AG42 | AB | 51.4 | 0.52 | 0.26 | 0.06 | 12.35 | 3.65 | 1.41 | -0.19 | 0.05 | -0.61 | 0.08 | ferruginous |
| AG37 | AB | 42 | 0.47 | 0.28 | 0.43 | 6.99 | 3.34 | 5.07 | -0.30 | 0.05 | 0.63 | 0.04 | ferruginous |
| AG33 | AB | 35.5 | 0.51 | 0.33 | 0.49 | 8.34 | 3.84 | 6.08 | -0.24 | 0.06 | 0.61 | 0.04 | ferruginous |
| AG30 | AB | 30.4 | 0.75 | 0.42 | 0.55 | 7.67 | 2.95 | 4.20 | -0.35 | 0.06 | 0.86 | 0.03 | weakly euxinic |
| AG28 | AB | 28.25 | 0.64 | 0.36 | 0.53 | 6.45 | 2.85 | 2.63 | -0.29 | 0.05 | 0.47 | 0.03 | ferruginous |
| AG25 | AB | 24.7 | 0.74 | 0.39 | 0.62 | 8.63 | 3.40 | 4.96 | -0.25 | 0.06 | 0.39 | 0.03 | weakly euxinic |
| AG21 | AB | 20.51 | 0.63 | 0.39 | 0.59 | 3.74 | 2.58 | 2.43 | -0.34 | 0.05 | 0.52 | 0.04 | weakly euxinic |
| AG18 | AB | 16.8 | 0.67 | 0.35 | 0.49 | 4.96 | 2.70 | 2.32 | -0.25 | 0.05 | 0.33 | 0.04 | ferruginous |
| AG16 | AB | 14.3 | 0.73 | 0.39 | 0.51 | 5.35 | 2.88 | 3.56 | -0.21 | 0.07 | 0.60 | 0.05 | weakly euxinic |
| AG9 | AB | 6.13 | 0.53 | 0.37 | 0.43 | 2.54 | 2.22 | 1.71 | -0.32 | 0.05 | -0.20 | 0.05 | ferruginous |
| AG4 | AB | 3.25 | 0.12 | 0.15 | 0.00 | 3.08 | 3.04 | 1.19 | -0.32 | 0.06 | / | 0.12 | ferruginous |
| AG2 | AB | 1.65 | 0.25 | 0.09 | 0.01 | 1.65 | 2.45 | 0.62 | -0.25 | 0.07 | 0.21 | 0.07 | ferruginous |

## *Table S6. Parameters used in the U-Mo mass balance model.*

| Parameter | U | Mo | Reference |
|---|---|---|---|
| $A_0$ | $3.6 \times 10^{14}$ m$^2$ | | *(144)* |
| $A_{euxinic0}$ | 0.0011 | | *(46)* |
| $A_{ferruginous0}$ | 0.0192 | | *(46)* |
| $A_{(dys)oxic0}$ | 1-$A_{euxinic0}$-$A_{ferruginous0}$ | | Mass balance |
| [Me] | $1.9 \times 10^{13}$ mol | $1.35 \times 10^{14}$ mol | *(144, 108)* |
| $\delta_{SW}$ | -0.39‰ | 2.34‰ | *(68, 108)* |
| $F_{river}$ | (27.5 – 56.5) × $10^6$ mol yr$^{-1}$ | (18 - 30) × $10^7$ mol yr$^{-1}$ | *(145, 146)* |
| $\delta_{river}$ | -0.24 ~ -0.34‰ | 0.5 ~ 0.9‰ | *(49, 108)* |
| $F_{euxinic0}$ | 5 × $10^6$ mol yr$^{-1}$ | 4.6 × $10^7$ mol yr$^{-1}$ | *(108)* |
| $F_{ferruginous0}$ | 15 × $10^6$ mol yr$^{-1}$ | 15.5 × $10^7$ mol yr$^{-1}$ | *(108)* |
| $F_{(dys)oxic0}$ | 22 × $10^6$ mol yr$^{-1}$ | 10.9 × $10^7$ mol yr$^{-1}$ | *(108)* |
| $\Delta_{euxinic}$ | 0.4 ~ 0.85‰ | 0 to –0.5‰ | *(49, 108)* |
| $\Delta_{ferruginous}$ | 0.1 ~ 0.4‰ | –2 to –0.5‰ | *(147, 149)* |
| $\Delta_{oxic}$ | -0.2 ~ 0‰ | –3 to –2 ‰ | *(108)* |

## REFERENCES


1. M. Calner, "Silurian global events—At the tipping point of climate change" in *Mass Extinction*, A. M. T. Elewa, Ed. (Springer, Berlin/Heidelberg, 2008), pp. 21–57.

2. R. A. Cooper, P. M. Sadler, A. Munnecke, J. S. Crampton, Graptoloid evolutionary rates track Ordovician–Silurian global climate change. *Geol. Mag.* **151**, 349–364 (2013).

3. Y. Wang, P. B. Wignall, Y. Xiong, D. K. Loydell, J. Peakall, J. H. Baas, B. J. W. Mills, S. W. Poulton, Marine redox dynamics and biotic response to the mid-Silurian Ireviken Extinction Event in a mid-shelf setting. *J. Geol. Soc.* **181**, jgs2023–155 (2024).

4. B. D. Cramer, C. E. Brett, M. J. Melchin, P. Männik, M. A. Kleffner, P. I. McLaughlin, D. K. Loydell, A. Munnecke, L. Jeppsson, C. Corradini, F. R. Brunton, M. R. Saltzman, Testing the limits of Paleozoic chronostratigraphic correlation via high-resolution (<500 k.y.) integrated conodont, graptolite, and carbon isotope ($\delta^{13}C_{carb}$) biochemostratigraphy across the Llandovery–Wenlock (Silurian) boundary: Is a unified Phanerozoic time scale achievable? *Geol. Soc. Am. Bull* **122**, 1700–1716 (2010).

5. O. Lehnert, P. Männik, M. M. Joachimski, M. Calner, J. Frýda, Palaeoclimate perturbations before the Sheinwoodian glaciation: A trigger for extinctions during the 'Ireviken Event'. *Palaeogeogr. Palaeoclimatol. Palaeoecol.* **296**, 320–331 (2010).

6. B. D. Cramer, T. R. A. Vandenbroucke, G. A. Ludvigson, High-resolution event stratigraphy (HiRES) and the quantification of stratigraphic uncertainty: Silurian examples of the quest for precision in stratigraphy. *Earth Sci. Rev.* **141**, 136–153 (2015).

7. E. J. Biebesheimer, B. D. Cramer, M. Calner, B. A. Barnett, S. C. Oborny, A. M. Bancroft, Asynchronous $\delta^{13}C_{carb}$ and $\delta^{13}C_{org}$ records during the onset of the Mulde (Silurian) positive carbon isotope excursion from the Altajme core, Gotland, Sweden. *Chem. Geol.* **576**, 120256 (2021).

8. J. Frýda, O. Lehnert, M. M. Joachimski, P. Männik, M. Kubajko, M. Mergl, J. Farkaš, B. Frýdová, The Mid-Ludfordian (late Silurian) glaciation: A link with global changes in ocean chemistry and ecosystem overturns. *Earth Sci. Rev.* **220**, 103652 (2021).

9. L. Jeppsson, An oceanic model for lithological and faunal changes tested on the Silurian record. *J. Geol. Soc.* **147**, 663–674 (1990).

10. T. M. Lenton, T. W. Dahl, S. J. Daines, B. J. W. Mills, K. Ozaki, M. R. Saltzman, P. Porada, Earliest land plants created modern levels of atmospheric oxygen. *Proc. Natl. Acad. Sci. U.S.A.* **113**, 9704–9709 (2016).

11. A. J. Krause, B. J. W. Mills, S. Zhang, N. J. Planavsky, T. M. Lenton, S. W. Poulton, C. T. Reinhard, X. Wang, A. Weiss, S. J. Daines, Stepwise oxygenation of the Paleozoic atmosphere. *Nat. Commun.* **9**, 4081 (2018).

12. S. A. Young, A. Kleinberg, J. D. Owens, Geochemical evidence for expansion of marine euxinia during an early Silurian (Llandovery–Wenlock boundary) mass extinction. *Earth Planet. Sci. Lett.* **513**, 187–196 (2019).

13. E. R. Hartke, B. D. Cramer, M. Calner, M. J. Melchin, B. A. Barnett, S. C. Oborny, E. A. Johnson, S. A. Young, Decoupling $\delta^{13}C_{carb}$ and $\delta^{13}C_{org}$ at the onset of the Ireviken carbon isotope excursion: $\Delta^{13}C$ and organic carbon burial ($f_{org}$) during a Silurian oceanic anoxic event. *Glob. Planet. Change* **196**, 103373 (2021).

14. J. Frieling, T. A. Mather, I. M. Fendley, H. C. Jenkyns, Z. Zhao, T. W. Dahl, B. A. Bergquist, K. Cheng, A. T. Nielsen, A. J. Dickson, No evidence for a volcanic trigger for late Cambrian carbon-cycle perturbations. *Geology* **52**, 12–16 (2024).

15. T. Reershemius, N. J. Planavsky, What controls the duration and intensity of ocean anoxic events in the Paleozoic and the Mesozoic? *Earth Sci. Rev.* **221**, 103787 (2021).

16. J. A. Trotter, I. S. Williams, C. R. Barnes, P. Männik, A. Simpson, New conodont $\delta^{18}O$ records of Silurian climate change: Implications for environmental and biological events. *Palaeogeogr. Palaeoclimatol. Palaeoecol.* **443**, 34–48 (2016).

17. A. Munnecke, C. Samtleben, T. Bickert, The Ireviken Event in the lower Silurian of Gotland, Sweden—Relation to similar Palaeozoic and Proterozoic events. *Palaeogeogr. Palaeoclimatol. Palaeoecol.* **195**, 99–124 (2003).

18. B. D. Cramer, M. A. Kleffner, C. E. Brett, P. I. McLaughlin, L. Jeppsson, A. Munnecke, C. Samtleben, P. Männik, M. J. Melchin, C. Corradini, F. R. Brunton, M. R. Saltzman, Paleobiogeography, high-resolution stratigraphy, and the future of Paleozoic biostratigraphy: Fine-scale diachroneity of the Wenlock (Silurian) conodont *Kockelella walliseri*. *Palaeogeogr. Palaeoclimatol. Palaeoecol.* **294**, 232–241 (2010).

19. L. Jeppsson, R. J. Aldridge, K. J. Dorning, Wenlock (Silurian) oceanic episodes and events. *J. Geol. Soc. London* **152**, 487–498 (1995).

20. D. K. Loydell, Early Silurian sea-level changes. *Geol. Mag.* **135**, 447–471 (1998).

21. D. Gelsthorpe, Microplankton changes through the early Silurian Ireviken extinction event on Gotland, Sweden. *Rev. Palaeobot. Palynol.* **130**, 89–103 (2004).

22. D. K. Loydell, R. Cave, The Llandovery–Wenlock boundary and related stratigraphy in eastern mid-Wales with special reference to the Banwy River section. *Newsl. Stratigr.* **34**, 39–64 (1996).

23. R. B. Rickards, "Northern England" in *A Global Standard for the Silurian System*, C. H. Holland, M. G. Bassett, Eds. (Geological Series 9, National Museum of Wales, 1989), pp. 116–131.

24. S. W. Poulton, D. E. Canfield, Ferruginous conditions: A dominant feature of the ocean through Earth's history. *Elements* **7**, 107–112 (2011).

25. S. Li, P. B. Wignall, S. W. Poulton, Co-application of rhenium, vanadium, uranium and molybdenum as paleo-redox proxies: Insight from modern and ancient environments. *Chem. Geol.* **674**, 122565 (2025).

26. R. B. Rickards, On some highest Llandovery red beds and graptolite assemblages in Britain and Eire. *Geol. Mag.* **110**, 70–72 (1973).

27. M. W. Hounslow, K. T. Ratcliffe, S. E. Harris, J. Nawrocki, K. Wójcik, P. Montgomery, N. H. Woodcock, The Telychian (early Silurian) oxygenation event in northern Europe: A

geochemical and magnetic perspective. *Palaeogeogr. Palaeoclimatol. Palaeoecol.* **567**, 110277 (2021).

28. E. J. Matheson, P. K. Pufahl, A. Voinot, J. B. Murphy, D. M. Fitzgerald, Ironstone as a proxy of Paleozoic ocean oxygenation. *Earth Planet. Sci. Lett.* **594**, 117715 (2022).

29. S. W. Poulton, *The Iron Speciation Paleoredox Proxy* (Cambridge Univ. Press, 2021).

30. M. J. Melchin, P. M. Sadler, B. D. Cramer, "The Silurian period" in *Geologic Time Scale 2020*, F. M. Gradstein, J. G. Ogg, M. D. Schmitz, G. M. Ogg, Eds. (Elsevier, 2020), pp. 695–732.

31. J. Rong, Y. Wang, X. Zhang, Tracking shallow marine red beds through geological time as exemplified by the lower Telychian (Silurian) in the Upper Yangtze Region, South China. *Sci. China Earth Sci.* **55**, 699–713 (2012).

32. D. E. Canfield, The geochemistry of river particulates from the continental USA: Major elements. *Geochim. Cosmochim. Acta* **61**, 3349–3365 (1997).

33. S. W. Poulton, R. Raiswell, The low-temperature geochemical cycle of iron: From continental fluxes to marine sediment deposition. *Am. J. Sci.* **302**, 774–805 (2002).

34. B. Kendall, T. Komiya, T. W. Lyons, S. M. Bates, G. W. Gordon, S. J. Romaniello, G. Jiang, R. A. Creaser, S. Xiao, K. McFadden, M. Ristroph, C. T. Reinhard, C. J. Thompson, C. K. Junium, C. Siebert, P. Fralick, B. A. Wing, A. D. Anbar, Uranium and molybdenum isotope evidence for an episode of widespread ocean oxygenation during the late Ediacaran Period. *Geochim. Cosmochim. Acta* **156**, 173–193 (2015).

35. R. G. Stockey, D. B. Cole, N. J. Planavsky, D. K. Loydell, J. Frýda, E. A. Sperling, Persistent global marine euxinia in the early Silurian. *Nat. Commun.* **11**, 1804 (2020).

36. A. J. Dickson, M.-L. Bagard, J. A. R. Katchinoff, M. Davies, S. W. Poulton, A. S. Cohen, Isotopic constraints on ocean redox at the end of the Eocene. *Earth Planet. Sci. Lett.* **562**, 116814 (2021).

37. A. Kunert, S. W. Poulton, D. E. Canfield, P. W. Fralick, G. J. Gilleaudeau, B. Kendall, Controls on uranium isotope fractionation in the late Paleoproterozoic ocean. *Earth Planet. Sci. Lett.* **666**, 119498 (2025).

38. R. F. Anderson, A. P. LeHuray, M. Q. Fleisher, J. W. Murray, Uranium deposition in Saanich Inlet sediments, vancouver island. *Geochim. Cosmochim. Acta* **53**, 2205–2213 (1989).

39. T. Goldberg, C. Archer, D. Vance, S. W. Poulton, Mo isotope fractionation during adsorption to Fe (oxyhydr)oxides. *Geochim. Cosmochim. Acta* **73**, 6502–6516 (2009).

40. T. Goldberg, C. Archer, D. Vance, B. Thamdrup, A. McAnena, S. W. Poulton, Controls on Mo isotope fractionations in a Mn-rich anoxic marine sediment, Gullmar Fjord, Sweden. *Chem. Geol.* **296**, 73–82 (2012).

41. J. Luo, X. Long, F. T. Bowyer, B. J. W. Mills, J. Li, Y. Xiong, X. Zhu, K. Zhang, S. W. Poulton, Pulsed oxygenation events drove progressive oxygenation of the early Mesoproterozoic ocean. *Earth Planet. Sci. Lett.* **559**, 116754 (2021).

42. N. Neubert, T. F. Nägler, M. E. Böttcher, Sulfidity controls molybdenum isotope fractionation into euxinic sediments: Evidence from the modern Black Sea. *Geology* **36**, 775–778 (2008).

43. T. Nägler, N. Neubert, M. Böttcher, O. Dellwig, B. Schnetger, Molybdenum isotope fractionation in pelagic euxinia: Evidence from the modern Black and Baltic Seas. *Chem. Geol.* **289**, 1–11 (2011).

44. C. T. Reinhard, N. J. Planavsky, L. J. Robbins, C. A. Partin, B. C. Gill, S. V. Lalonde, A. Bekker, K. O. Konhauser, T. W. Lyons, Proterozoic ocean redox and biogeochemical stasis. *Proc. Natl. Acad. Sci. U.S.A.* **110**, 5357–5362 (2013).

45. Y. Song, B. J. W. Mills, F. T. Bowyer, M. B. Andersen, F. Ossa Ossa, A. J. Dickson, J. Harvey, S. Zhang, X. Wang, H. Wang, D. E. Canfield, G. A. Shields, S. W. Poulton, Tracking the spatial extent of redox variability in the mid-Proterozoic ocean. *Geology* **53**, 785–789 (2025).

46. R. L. Rutledge, G. J. Gilleaudeau, M. N. Remírez, A. J. Kaufman, T. W. Lyons, S. Bates, T. J. Algeo, Productivity and organic carbon loading control uranium isotope behavior in ancient reducing settings: Implications for the paleoredox proxy. *Geochim. Cosmochim. Acta* **368**, 197–213 (2024).

47. M. B. Andersen, C. H. Stirling, S. Weyer, Uranium isotope fractionation. *Rev. Mineral. Geochem.* **82**, 799–850 (2017).

48. M. N. Remírez, G. J. Gilleaudeau, X. Gan, M. A. Kipp, F. L. H. Tissot, A. J. Kaufman, M. Parente, Carbonate uranium isotopes record global expansion of marine anoxia during the Toarcian Oceanic Anoxic Event. *Proc. Natl. Acad. Sci. U.S.A.* **121**, e2406032121 (2024).

49. M. J. Costello, A. Cheung, N. De Hauwere, Surface area and the seabed area, volume, depth, slope, and topographic variation for the world's seas, oceans, and countries. *Environ. Sci. Technol.* **44**, 8821–8828 (2010).

50. E. R. Haxen, N. H. Schovsbo, A. T. Nielsen, S. Richoz, D. K. Loydell, N. R. Posth, D. E. Canfield, E. U. Hammarlund, "Hypoxic" Silurian oceans suggest early animals thrived in a low-$O_2$ world. *Earth Planet. Sci. Lett.* **622**, 118416 (2023).

51. D. K. Loydell, J. Frýda, Carbon isotope stratigraphy of the upper Telychian and lower Sheinwoodian (Llandovery–Wenlock, Silurian) of the Banwy River section. *Wales. Geol. Mag.* **144**, 1015–1019 (2007).

52. H. C. Jenkyns, Geochemistry of oceanic anoxic events. *Geochem. Geophys. Geosyst.* **11**, Q03004 (2010).

53. D. C. Ray, E. Jarochowska, H. E. Hughes, A. S. Richardson, A. T. Thomas, Glacio-eustatic sea-level changes and their magnitude in the Telychian–Sheinwoodian (Silurian) of England and Wales. *J. Geol. Soc.* **182**, jgs2024–292 (2025).

54. Z. Qiu, C. Zou, B. J. W. Mills, Y. Xiong, H. Tao, W. Lu, X. Liu, S. Xiao, S. W. Poulton, A nutrient control on expanded anoxia and global cooling during the Late Ordovician mass extinction. *Commun. Earth Environ.* **3**, 34 (2022).

55. R. Guilbaud, S. W. Poulton, J. Thompson, K. F. Husband, M. Zhu, Y. Zhou, G. A. Shields, T. M. Lenton, Phosphorus-limited conditions in the early Neoproterozoic ocean maintained low levels of atmospheric oxygen. *Nat. Geosci.* **13**, 296–301 (2020).

56. J.-X. Fan, S.-Z. Shen, D. H. Erwin, P. M. Sadler, N. M. Leod, Q.-M. Cheng, X.-D. Hou, J. Yang, X.-D. Wang, Y. Wang, H. Zhang, X. Chen, G.-X. Li, Y.-C. Zhang, Y.-K. Shi, D.-X. Yuan, Q. Chen, L.-N. Zhang, C. Li, Y.-Y. Zhao, A high-resolution summary of Cambrian to Early Triassic marine invertebrate biodiversity. *Science* **367**, 272–277 (2020).

57. A. Hallam, P. B. Wignall, Mass extinctions and sea-level changes. *Earth Sci. Rev.* **48**, 217–250 (1999).

58. S. W. Poulton, D. E. Canfield, Development of a sequential extraction procedure for iron: Implications for iron partitioning in continentally derived particulates. *Chem. Geol.* **214**, 209–221 (2005).

59. D. E. Canfield, R. Raiswell, J. T. Westrich, C. M. Reaves, R. A. Berner, The use of chromium reduction in the analysis of reduced inorganic sulfur in sediments and shales. *Chem. Geol.* **54**, 149–155 (1986).

60. L. J. Alcott, A. J. Krause, E. U. Hammarlund, C. J. Bjerrum, F. Scholz, Y. Xiong, A. J. Hobson, L. Neve, B. J. W. Mills, C. März, B. Schnetger, A. Bekker, S. W. Poulton, Development of iron speciation reference materials for palaeoredox analysis. *Geostand. Geoanal. Res.* **44**, 581–591 (2020).

61. C. R. Pearce, A. S. Cohen, I. J. Parkinson, Quantitative separation of molybdenum and rhenium from geological materials for isotopic determination by MC-ICP-MS. *Geostand. Geoanal. Res.* **33**, 219–229 (2009).

62. A. J. Dickson, H. C. Jenkyns, D. Porcelli, S. van den Boorn, E. Idiz, Basin-scale controls on the molybdenum-isotope composition of seawater during Oceanic Anoxic Event 2 (Late Cretaceous). *Geochim. Cosmochim. Acta* **178**, 291–306 (2016).

63. C. H. Stirling, M. B. Andersen, E. K. Potter, A. N. Halliday, Low-temperature isotopic fractionation of uranium. *Earth Planet. Sci. Lett.* **264**, 208–225 (2007).

64. T. Goldberg, G. Gordon, G. Izon, C. Archer, C. R. Pearce, J. McManus, A. D. Anbar, M. Rehkämper, Resolution of inter-laboratory discrepancies in Mo isotope data: An intercalibration. *J. Anal. At. Spectrom* **28**, 724–735 (2013).

65. M. Sun, C. Archer, D. Vance, New methods for the chemical isolation and stable isotope measurement of multiple transition metals, with application to the Earth sciences. *Geostand. Geoanal. Res.* **45**, 643–658 (2021).

66. F. L. H. Tissot, N. Dauphas, Uranium isotopic compositions of the crust and ocean: Age corrections, U budget and global extent of modern anoxia. *Geochim. Cosmochim. Acta* **167**, 113–143 (2015).

67. T. H. Torsvik, L. R. M. Cocks, *Earth History and Palaeogeography* (Cambridge Univ. Press, 2017).

68. B. D. Cramer, I. Jarvis, "Carbon isotope stratigraphy" in *Geologic Time Scale 2020* (Elsevier, 2020), pp. 309–343.

69. L. Jeppsson, A new latest Telychian, Sheinwoodian and early Homerian (Early Silurian) standard conodont zonation. *Trans. R. Soc. Edinb. Earth Sci.* **88**, 91–114 (1997).

70. R. J. Aldridge, L. Jeppsson, K. J. Dorning, Early Silurian oceanic episodes and events. *J. Geol. Soc.* **150**, 501–513 (1993).

71. D. K. Loydell, Early Silurian positive $\delta^{13}C$ excursions and their relationship to glaciations, sea-level changes and extinction events. *Geol. J.* **42**, 531–546 (2007).

72. C. V. Rose, W. W. Fischer, S. Finnegan, D. A. Fike, Records of carbon and sulfur cycling during the Silurian Ireviken Event in Gotland, Sweden. *Geochim. Cosmochim. Acta* **246**, 299–316 (2019).

73. D. Kaljo, T. Martma, B. E. E. Neuman, K. Rønning, "Carbon isotope dating of several uppermost Ordovician and lower Silurian sections in the Oslo Region, Norway," in *WOGOGOB-2004: 8th Meeting on the Working Group on the Ordovician Geology of Baltoscandia–Conference Materials: Abstracts and Field Guidebook*, O. Hints, L. Ainsaar, Eds. (Tartu Univ. Press, 2004), pp. 51–52.

74. U. Brand, K. Azmy, J. Veizer, Evaluation of the Salinic I tectonic, Cancañiri glacial and Ireviken biotic events: Biochemostratigraphy of the Lower Silurian succession in the Niagara Gorge area, Canada and U.S.A. *Palaeogeogr. Palaeoclimatol. Palaeoecol.* **241**, 192–213 (2006).

75. M. R. Saltzman, Silurian $\delta^{13}C$ stratigraphy: A view from North America. *Geology* **29**, 671–674 (2001).

76. D. Kaljo, T. Martma, Application of carbon isotope stratigraphy to dating the Baltic Silurian rocks. *GFF* **128**, 123–129 (2006).

77. H. E. Hughes, D. C. Ray, The carbon isotope and sequence stratigraphic record of the Sheinwoodian and lower Homerian stages (Silurian) of the Midland Platform, UK. *Palaeogeogr. Palaeoclimatol. Palaeoecol.* **445**, 97–114 (2016).

78. D. K. Loydell, J. Frýda, A. Butcher, R. F. Loveridge, A new high-resolution $\delta^{13}C_{carb}$ isotope curve through the lower Wenlock Series of Buttington Quarry, Wales. *GFF* **136**, 172–174 (2014).

79. D. K. Loydell, R. R. Large, Biotic, geochemical and environmental changes through the early Sheinwoodian (Wenlock, Silurian) carbon isotope excursion (ESCIE), Buttington Quarry, Wales. *Palaeogeogr. Palaeoclimatol. Palaeoecol.* **514**, 305–325 (2019).

80. S. M. McLennan, Relationships between the trace element composition of sedimentary rocks and upper continental crust. *Geochem. Geophys. Geosyst.* **2**, 2000GC000109 (2001).

81. K. Chen, R. J. Walker, R. L. Rudnick, S. Gao, R. M. Gaschnig, I. S. Puchtel, M. Tang, Z.-C. Hu, Platinum-group element abundances and Re–Os isotopic systematics of the upper

continental crust through time: Evidence from glacial diamictites. *Geochim. Cosmochim. Acta* **191**, 1–16 (2016).

82. L. R. Kump, M. A. Arthur, Interpreting carbon-isotope excursions: Carbonates and organic matter. *Chem. Geol.* **161**, 181–198 (1999).

83. R. B. Rickards, N. H. Woodcock, Stratigraphical revision of the Windermere Supergroup (Late Ordovician–Silurian) in the southern Howgill Fells, NW England. *Proc. Yorks. Geol. Soc.* **55**, 263–285 (2005).

84. V. Pasquier, D. A. Fike, S. Revillon, I. Halevy, A global reassessment of the controls on iron speciation in modern sediments and sedimentary rocks: A dominant role for diagenesis. *Geochim. Cosmochim. Acta* **335**, 211–230 (2022).

85. R. Raiswell, D. E. Canfield, Sources of iron for pyrite formation in marine sediments. *Am. J. Sci.* **298**, 219–245 (1998).

86. R. Raiswell, D. S. Hardisty, T. W. Lyons, D. E. Canfield, J. D. Owens, N. J. Planavsky, S. W. Poulton, C. T. Reinhard, The iron paleoredox proxies: A guide to the pitfalls, problems and proper practice. *Am. J. Sci.* **318**, 491–526 (2018).

87. R. Raiswell, R. Newton P. B. Wignall., An indicator of water-column anoxia: Resolution of biofacies variations in the Kimmeridge Clay (Upper Jurassic, U.K.). *J. Sediment. Res.* **71**, 286–294 (2001).

88. M. O. Clarkson, S. W. Poulton, R. G. R. A. Wood, Assessing the utility of Fe/Al and Fe-speciation to record water-column redox conditions in carbonate-rich sediments. *Chem. Geol.* **382**, 111–122 (2014).

89. T. F. Anderson, R. Raiswell, Sources and mechanisms for the enrichment of highly reactive iron in euxinic Black Sea sediments. *Am. J. Sci.* **304**, 203–233 (2004).

90. C. März, S. W. Poulton, B. Beckmann, T. Küster, T. Wagner, S. Kasten, Redox sensitivity of P cycling during marine black shale formation: Dynamics of sulfidic and anoxic, non-sulfidic bottom waters. *Geochim. Cosmochim. Acta* **72**, 3703–3717 (2008).

91. A. Benkovitz, A. Matthews, N. Teutsch, S. W. Poulton, M. Bar-Matthews, A. Almogi-Labin, Tracing water-column euxinia in Eastern Mediterranean Sapropels S5 and S7. *Chem. Geol.* **545**, 119627 (2020).

92. J. L. Morford, W. R. Martin, R. François, C. M. Carney, A model for uranium, rhenium, and molybdenum diagenesis in marine sediments based on results from coastal locations. *Geochim. Cosmochim. Acta* **73**, 2938–2960 (2009).

93. S. E. Calvert, T. F. Pedersen, Geochemistry of recent oxic and anoxic marine sediments: Implications for the geological record. *Mar. Geol.* **113**, 67–88 (1993).

94. Y. Zheng, R. F. Anderson, A. van Geen, J. Kuwabara, Authigenic molybdenum formation in marine sediments: A link to pore-water sulfide in the Santa Barbara Basin. *Geochim. Cosmochim. Acta* **64**, 4165–4178 (2000).

95. G. R. Helz, C. V. Miller, J. M. Charnock, J. F. W. Mosselmans, R. A. D. Pattrick, C. D. Garner, D. J. Vaughan, Mechanism of molybdenum removal from the sea and its concentration in black shales: EXAFS evidence. *Geochim. Cosmochim. Acta* **60**, 3631–3642 (1996).

96. Y. Zheng, R. F. Anderson, A. van Geen, M. Q. Fleisher, Preservation of particulate non-lithogenic uranium in marine sediments. *Geochim. Cosmochim. Acta* **66**, 3085–3092 (2002).

97. J. Crusius, S. Calvert, T. Pedersen, D. Sage, Rhenium and molybdenum enrichments in sediments as indicators of oxic, suboxic and sulfidic conditions of deposition. *Earth Planet. Sci. Lett.* **145**, 65–78 (1996).

98. T. J. Algeo, C. Li, Redox classification and calibration of redox thresholds in sedimentary systems. *Geochim. Cosmochim. Acta* **287**, 8–26 (2020).

99. Y. Wang, P. B. Wignall, J. Peakall, J. H. Baas, S. W. Poulton, Softgrounds: Substrates controlled by sediment gravity flows and the evolution of deep-water trace fossils. *Geol. Soc. Lond. Spec. Publ.* **556**, 121–140 (2025).

100. N. Tribovillard, T. J. Algeo, F. Baudin, A. Riboulleau, Analysis of marine environmental conditions based on molybdenum–uranium covariation—Applications to Mesozoic paleoceanography. *Chem. Geol.* **324-325**, 46–58 (2012).

101. J. L. Morford, S. Emerson, The geochemistry of redox-sensitive trace metals in sediments. *Geochim. Cosmochim. Acta* **63**, 1735–1750 (1999).

102. T. He, P. B. Wignall, R. J. Newton, J. W. Atkinson, J. F. J. Keeling, Y. Xiong, S. W. Poulton, Extensive marine anoxia in the European epicontinental sea during the end-Triassic mass extinction. *Glob. Planet. Change* **210**, 103771 (2022).

103. S. Li, P. B. Wignall, Y. Xiong, S. W. Poulton, Calibration of redox thresholds in black shale: Insight from a stratified Mississippian basin with warm saline bottom waters. *Geol. Soc. Am. Bull.* **136**, 1266–1286 (2023).

104. A. R. Voegelin, T. Pettke, N. D. Greber, B. von Niederhäusern, T. F. Nägler, Magma differentiation fractionates Mo isotope ratios: Evidence from the Kos Plateau Tuff (Aegean Arc). *Lithos* **190-191**, 440–448 (2014).

105. K. V. Lau, L. G. Hancock, S. Severmann, A. Kuzminov, D. B. Cole, R. J. Behl, N. J. Planavsky, T. W. Lyons, Variable local basin hydrography and productivity control the uranium isotope paleoredox proxy in anoxic black shales. *Geochim. Cosmochim. Acta* **317**, 433–456 (2022).

106. D. B. Cole, N. J. Planavsky, M. Longley, P. Böning, D. Wilkes, X. Wang, E. D. Swanner, C. Wittkop, D. K. Loydell, V. Busigny, A. Knudsen, E. A. Sperling, Uranium isotope fractionation in non-sulfidic anoxic settings and the global uranium isotope mass balance. *Global Biogeochem. Cycles* **34**, e2020GB006649 (2020).

107. B. Kendall, G. A. Brennecka, L. E. Wasylenki, A. D. Anbar, G. W. Gordon, J. Wang, W. Zheng, S. J. Romaniello, D. J. Over, Y. Bennett, L. Xing, A. Kunert, C. Boyes, J. Liu, Inverse correlation between the molybdenum and uranium isotope compositions of Upper Devonian black shales caused by changes in local depositional conditions rather than global ocean redox variations. *Geochim. Cosmochim. Acta* **287**, 141–164 (2020).

108. B. Kendall, T. W. Dahl, A. D. Anbar, The stable isotope geochemistry of molybdenum. *Rev. Mineral. Geochem.* **82**, 683–732 (2017).

109. M. B. Andersen, A. Matthews, M. Bar-Matthews, D. Vance, Rapid onset of ocean anoxia shown by high U and low Mo isotope compositions of sapropel S1. *Geochem. Perspect. Lett.* **15**, 10–14 (2020).

110. X. Lu, T. W. Dahl, W. Zheng, S. Wang, B. Kendall, Estimating ancient seawater isotope compositions and global ocean redox conditions by coupling the molybdenum and uranium isotope systems of euxinic organic-rich mudrocks. *Geochim. Cosmochim. Acta* **290**, 76–103 (2020).

111. J. Barling, G. L. Arnold, A. D. Anbar, Natural mass-dependent variations in the isotopic composition of molybdenum. *Earth Planet. Sci. Lett.* **193**, 447–457 (2001).

112. C. Siebert, T. F. Nägler, F. von Blanckenburg, J. D. Kramers, Molybdenum isotope records as a potential new proxy for paleoceanography. *Earth Planet. Sci. Lett.* **211**, 159–171 (2003).

113. Y. Nakagawa, S. Takano, M. L. Firdaus, K. Norisuye, T. Hirata, D. Vance, Y. Sohrin, The molybdenum isotopic composition of the modern ocean. *Geochem. J.* **46**, 131–141 (2012).

114. J. McManus, T. F. Nägler, C. Siebert, C. G. Wheat, D. E. Hammond, Oceanic molybdenum isotope fractionation: Diagenesis and hydrothermal ridge-flank alteration. *Geochem. Geophys. Geosyst.* **3**, 1078 (2002).

115. R. L. Poulson, C. Siebert, J. McManus, W. M. Berelson, Authigenic molybdenum isotope signatures in marine sediments. *Geology* **34**, 617–620 (2006).

116. C. Siebert, J. McManus, A. Bice, R. Poulson, W. M. Berelson, Molybdenum isotope signatures in continental margin marine sediments. *Earth Planet. Sci. Lett.* **241**, 723–733 (2006).

117. R. L. Poulson Brucker, J. McManus, S. Severmann, W. M. Berelson, Molybdenum behavior during early diagenesis: Insights from Mo isotopes. *Geochem. Geophys. Geosyst.* **10**, doi.org/10.1029/2008GC002180 (2009).

118. S. Weyer, A. D. Anbar, A. Gerdes, G. W. Gordon, T. J. Algeo, E. A. Boyle, Natural fractionation of 238U/235U. *Geochim. Cosmochim. Acta* **72**, 345–359 (2008).

119. C. Holmden, M. Amini, R. François, Uranium isotope fractionation in Saanich Inlet: A modern analog study of a paleoredox tracer. *Geochim. Cosmochim. Acta* **153**, 202–215 (2015).

120. A. Brüske, S. Weyer, M. Y. Zhao, N. J. Planavsky, A. Wegwerth, N. Neubert, O. Dellwig, K. V. Lau, T. W. Lyons, Correlated molybdenum and uranium isotope signatures in modern anoxic sediments: Implications for their use as paleo-redox proxy. *Geochim. Cosmochim. Acta* **270**, 449–474 (2020).

121. G.-Y. Wei, N. J. Planavsky, L. G. Tarhan, T. He, D. Wang, G. A. Shields, W. Wei, H.-F. Ling, Highly dynamic marine redox state through the Cambrian explosion highlighted by authigenic δ238U records. *Earth Planet. Sci. Lett.* **544**, 116361 (2020).

122. M. B. Andersen, D. Vance, J. L. Morford, E. Bura-Nakić, S. F. M. Breitenbach, L. Och, Closing in on the marine $^{238}U/^{235}U$ budget. *Chem. Geol.* **420**, 11–22 (2016).

123. G. A. Brennecka, L. E. Wasylenki, J. R. Bargar, S. Weyer, A. D. Anbar, Uranium isotope fractionation during adsorption to Mn-oxyhydroxides. *Environ. Sci. Technol.* **45**, 1370–1375 (2011).

124. K. T. Goto, A. D. Anbar, G. W. Gordon, S. J. Romaniello, G. Shimoda, Y. Takaya, A. Tokumaru, T. Nozaki, K. Suzuki, S. Machida, T. Hanyu, A. Usui, Uranium isotope systematics of ferromanganese crusts in the Pacific Ocean: Implications for the marine 238U/235U isotope system. *Geochim. Cosmochim. Acta* **146**, 43–58 (2014).

125. T. W. Dahl, E. U. Hammarlund, A. D. Anbar, D. P. G. Bond, B. C. Gill, G. W. Gordon, A. H. Knoll, A. T. Nielsen, N. H. Schovsbo, D. E. Canfield, Devonian rise in atmospheric oxygen

correlated to the radiations of terrestrial plants and large predatory fish. *Proc. Natl. Acad. Sci. U.S.A.* **107**, 17911–17915 (2010).

126. X. Chen, H.-F. Ling, D. Vance, G. A. Shields-Zhou, M. Zhu, S. W. Poulton, L. M. Och, S.-Y. Jiang, D. Li, L. Cremonese, C. Archer, Rise to modern levels of ocean oxygenation coincided with the Cambrian radiation of animals. *Nat. Commun.* **6**, 7142 (2015).

127. M. O. Clarkson, T. M. Lenton, M. B. Andersen, M.-L. Bagard, A. J. Dickson, D. Vance, Upper limits on the extent of seafloor anoxia during the PETM from uranium isotopes. *Nat. Commun.* **12**, 399 (2021).

128. Y. Grahn, M. V. Caputo, Early Silurian glaciations in Brazil. *Palaeogeogr. Palaeoclimatol. Palaeoecol.* **99**, 9–15 (1992).

129. M. V. Caputo, "Ordovician–Silurian glaciations and global sea-level changes" in *Silurian Cycles: Linkages of Dynamic Stratigraphy with Atmospheric, Oceanic and Tectonic Changes*, E. Landing, M. E. Johnson, Eds. (Bulletin 491, New York State Museum, 1998), pp. 15–25.

130. E. Díaz-Martínez, Y. Grahn, Early Silurian glaciation along the western margin of Gondwana (Peru, Bolivia and northern Argentina): Palaeogeographic and geodynamic setting. *Palaeogeogr. Palaeoclimatol. Palaeoecol.* **245**, 62–81 (2007).

131. K. Azmy, J. Veizer, B. Wenzel, M. G. Bassett, P. Copper, Early Silurian strontium isotope stratigraphy. *Geol. Soc. Am. Bull.* **111**, 475–483 (1999).

132. J. C. Gouldey, M. R. Saltzman, S. A. Young, D. Kaljo, Strontium and carbon isotope stratigraphy of the Llandovery (Early Silurian): Implications for tectonics and weathering. *Palaeogeogr. Palaeoclimatol. Palaeoecol.* **296**, 264–275 (2010).

133. A. D. Sproson, P. A. E. Pogge von Strandmann, D. Selby, E. Jarochowska, J. Frýda, J. Hladil, D. K. Loydell, L. Slavík, M. Calner, G. Maier, A. Munnecke, T. M. Lenton, Osmium and lithium isotope evidence for weathering feedbacks linked to orbitally paced organic carbon burial and Silurian glaciations. *Earth Planet. Sci. Lett.* **577**, 117260 (2022).

134. H. W. Nesbitt, G. M. Young, Early Proterozoic climates and plate motions inferred from major element chemistry of lutites. *Nature* **299**, 715–717 (1982).

135. C. M. Fedo, H. W. Nesbitt, G. M. Young, Unraveling the effects of potassium metasomatism in sedimentary rocks and paleosols, with implications for paleoweathering conditions and provenance. *Geology* **23**, 921–924 (1995).

136. S. M. McLennan, Weathering and global denudation. *J. Geol.* **101**, 295–303 (1993).

137. N. D. Sheldon, N. J. Tabor, Quantitative paleoenvironmental and paleoclimatic reconstruction using paleosols. *Earth Sci. Rev.* **95**, 1–52 (2009).

138. J. Chen, I. P. Montañez, S. Zhang, T. T. Isson, S. I. Macarewich, N. J. Planavsky, F. Zhang, S. Rauzi, K. Daviau, L. Yao, Y.-P. Qi, Y. Wang, J.-X. Fan, C. J. Poulsen, A. D. Anbar, S.-Z. Shen, X.-D. Wang, Marine anoxia linked to abrupt global warming during Earth's penultimate icehouse. *Proc. Natl. Acad. Sci. U.S.A.* **119**, e2115231119 (2022).

139. F. M. Monteiro, R. D. Pancost, A. Ridgwell, Y. Donnadieu, Nutrients as the dominant control on the spread of anoxia and euxinia across the Cenomanian–Turonian oceanic anoxic event (OAE2): Model–data comparison. *Paleoceanogr. Paleoclimatol.* **27**, doi.org/10.1029/2012PA002351 (2012).

140. E. A. Sperling, M. J. Melchin, T. Fraser, R. G. Stockey, U. C. Farrell, L. Bhajan, T. N. Brunoir, D. B. Cole, B. C. Gill, A. Lenz, D. K. Loydell, J. Malinowski, A. J. Miller, S. Plaza-Torres, B. Bock, A. D. Rooney, S. A. Tecklenburg, J. M. Vogel, N. J. Planavsky, J. V. Strauss, A long-term record of early to mid-Paleozoic marine redox change. *Sci. Adv.* **7**, eabf4382 (2021).

141. R. G. Stockey, D. B. Cole, U. C. Farrell, H. Agić, T. H. Boag, J. J. Brocks, D. E. Canfield, M. Cheng, P. W. Crockford, H. Cui, T. W. Dahl, M. Del Mouro, K. Dewing, S. Q. Dornbos, J. F. Emmings, R. R. Gaines, T. M. Gibson, B. C. Gill, G. J. Gilleaudeau, K. Goldberg, R. Guilbaud, G. Halverson, E. U. Hammarlund, K. Hantsoo, M. A. Henderson, M. S. W. Hodgskiss, A. J. M. Jarrett, D. T. Johnston, P. Kabanov, J. Kimmig, A. H. Knoll, M. Kunzmann, M. A. LeRoy, C. Li, D. K. Loydell, F. A. Macdonald, J. M. Magnall, N. T.

Mills, L. M. Och, B. O'Connell, A. Pagès, S. E. Peters, S. M. Porter, S. W. Poulton, S. R. Ritzer, A. D. Rooney, S. Schoepfer, E. F. Smith, J. V. Strauss, G. J. Uhlein, T. White, R. A. Wood, C. R. Woltz, I. Yurchenko, N. J. Planavsky, E. A. Sperling, Sustained increases in atmospheric oxygen and marine productivity in the Neoproterozoic and Palaeozoic eras. *Nat. Geosci.* **17**, 667–674 (2024).

142. S. W. Poulton, S. Henkel, C. März, H. Urquhart, S. Flögel, S. Kasten, A continental-weathering control on orbitally driven redox–nutrient cycling during Cretaceous Oceanic Anoxic Event 2. *Geology* **43**, 963–966 (2015).

143. M. Schobben, W. J. Foster, A. R. N. Sleveland, V. Zuchuat, H. H. Svensen, S. Planke, D. P. G. Bond, F. Marcelis, R. J. Newton, P. B. Wignall, S. W. Poulton, A nutrient control on marine anoxia during the end-Permian mass extinction. *Nat. Geosci.* **13**, 640–646 (2020).

144. C. E. Barnes, J. K. Cochran, Uranium removal in oceanic sediments and the oceanic U balance. *Earth Planet. Sci. Lett.* **97**, 94–101 (1990).

145. R. M. Dunk, R. A. Mills, W. J. Jenkins, A reevaluation of the oceanic uranium budget for the Holocene. *Chem. Geol.* **190**, 45–67 (2002).

146. C. Scott, T. W. Lyons, A. Bekker, Y. Shen, S. W. Poulton, X. Chu, A. D. Anbar, Tracing the stepwise oxygenation of the Proterozoic ocean. *Nature* **452**, 456–459 (2008).

147. G. J. Gilleaudeau, S. J. Romaniello, G. Luo, A. J. Kaufman, F. Zhang, R. M. Klaebe, L. C. Kah, K. Azmy, J. K. Bartley, W. Zheng, A. H. Knoll, A. D. Anbar, Uranium isotope evidence for limited euxinia in mid-Proterozoic oceans. *Earth Planet. Sci. Lett.* **521**, 150–157 (2019).

148. G.-Y. Wei, N. J. Planavsky, T. He, F. Zhang, R. G. Stockey, D. B. Cole, Y.-B. Lin, H.-F. Ling, Global marine redox evolution from the late Neoproterozoic to the early Paleozoic constrained by the integration of Mo and U isotope records. *Earth Sci. Rev.* **214**, 103506 (2021).
